# Fibrin scaffolds for angiogenesis in soft tissue models: a systematic review

**DOI:** 10.1016/j.bioactmat.2025.10.019

**Published:** 2025-12-04

**Authors:** Carla Verónica Fuenteslópez, Simge Bahcevanci, Viorica Patrulea, Hua Ye

**Affiliations:** a Institute of Biomedical Engineering, Department of Engineering Science, University of Oxford, Old Road Campus Research Building, Roosevelt Drive, Oxford, OX3 7DQ, United Kingdom; b Institute of Pharmaceutical Sciences of Western Switzerland, University of Geneva, 1211 Geneva, Switzerland; c School of Pharmaceutical Sciences, University of Geneva, 1211 Geneva, Switzerland

**Keywords:** Fibrin scaffolds, Soft tissue regeneration, Microvascular network formation, Endothelial cell migration, Angiogenesis, Scaffold design optimisation

## Abstract

**Background:**

Fibrin is a biocompatible, angiogenic biomaterial widely used in soft tissue engineering, with outcomes influenced by scaffold formulation and design.

**Aim:**

This systematic review evaluates how fibrin scaffold composition and design affect angiogenesis in *in vitro* and *in vivo* soft tissue models.

**Methods:**

PubMed, Scopus, and OVID were searched on 28/Oct/2024. A two‐step screening process by three independent researchers identified original studies on fibrin scaffolds assessing endothelial formation and/or migration. Studies without experimental data or focused solely on grafts were excluded. Data on scaffold composition, manufactured objects, cell‐embedding strategies, angiogenic outcomes, and a subset of muscle‐specific studies were narratively synthesised. Risk of bias (RoB) and study quality were assessed using SYRCLE's RoB tool and a modified CAMARADES checklist.

**Results & discussion:**

The 81 studies highlight the impact of scaffold composition on angiogenic outcomes, with human‐derived fibrinogen and pre‐embedding cells consistently supporting successful outcomes. While tube and network formation outcomes typically aligned, endothelial migration exhibited different patterns. Thrombin often contributed positively, but crosslinker effects were less clear. Muscle‐focused studies mainly used hydrogels and often included non‐endothelial cells.

**Conclusions:**

Fibrin scaffolds are highly relevant for soft tissue engineering, with outcomes influenced by formulation, fibrinogen source, and cell embedding. However, experimental design variability and lack of standardised reporting hinder reproducibility and clinical translation. To support future research, a minimum information checklist was created to promote consistent reporting, while aggregated success rates across design parameters could guide scaffold design.

**Registration & funding:**

PROSPERO [CRD42025612994] and OSF [10.17605/osf.io/nvfdj]. No funding body was directly involved.

## Highlights

• Human fibrinogen & cell pre‐embedding are the most critical factors for angiogenesis.
• Tube & network formation outcomes often align but may differ from endothelial migration.
• Crosslinkers, such as *CaCl*_*2*_, improve scaffold stability but may impair angiogenesis.
• Scaffold types vary widely in muscle studies, showing no consensus on optimal design.
• Lack of standardised reporting limits reproducibility & clinical translation.

## Graphical abstract

**Figure:**
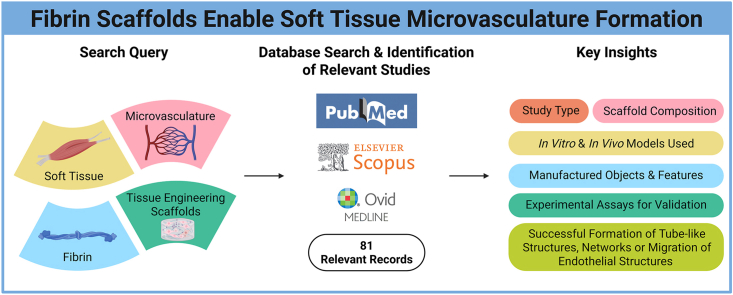

## 1 Introduction

Tissue engineering is a multidisciplinary field focused on replacing, repairing, and regenerating damaged tissue, ultimately aiming to reduce or eliminate the need for donor organs in modern medicine [1]. It integrates three main components: scaffolds, cells, and biological factors. Scaffolds are three‐dimensional (3D) matrices that provide structural support to cells, facilitating essential functions such as cell attachment, migration, and proliferation [2]. Scaffolds can take various forms, including pre‐made porous structures, decellularised extracellular matrix (ECM), or hydrogels [3,4]. A key aspect of scaffolds is that they can be customised to better replicate the natural tissue, enabling their use for both soft and hard tissue engineering applications [3].

Soft tissues comprise the non‐skeletal, supportive structures of the body; for instance, skin, blood and lymph vessels, adipose tissue, connective tissue, and peripheral nerves [5]. They also include musculoskeletal tissues, such as cartilage, ligaments, tendons, muscles, and connective tissues [6]. The specific design criteria for scaffolds, such as mechanical strength, depend on the tissue of interest, which dictates the choice of materials [7].

Both synthetic and natural polymers are widely used to create soft tissue scaffolds. Natural polymers (*e.g.,* collagen, fibrin, chitosan, and hyaluronic acid) typically offer superior biocompatibility but have limited processability compared to synthetic polymers. To achieve clinical success and enable physiologically relevant models for pre‐clinical research, scaffolds must exhibit specific biological, mechanical, and structural properties [8]. This includes being biocompatible and non‐toxic to prevent inducing host immune responses or reactions [9] and having suitable mechanical properties to withstand mechanical stress. Additionally, scaffolds should exhibit degradability profiles similar to those of the natural tissue they are mimicking while maintaining physical integrity and stability [10].

Porosity is another critical factor in scaffold design supporting cellular functions, especially when pores are interconnected, as they facilitate waste removal, as well as nutrient and oxygen exchange [11]. For larger tissues, vascularisation is essential to ensure adequate nutrient and oxygen delivery and metabolic waste removal [12]. Relevantly, the main challenge in tissue engineering is the insufficient vascularisation of the constructs, which limits cell survival, tissue integration [13] and, in turn, restricts the size of functional artificial tissues, as the absence of microvasculature prevents essential nutrient and oxygen delivery throughout the tissue. The increasing prevalence of soft tissue injuries and defects, coupled with the limited effectiveness of current reconstructive techniques, highlights the need for a comprehensive assessment of the angiogenic potential of fibrin‐based scaffolds in promoting angiogenesis, including microvascular formation in soft tissues [14,15].

Vascular network development can occur through vasculogenesis (the *de novo* formation of blood vessels from early endothelial cells (ECs) or angiogenesis (the sprouting of new blood vessels from pre‐existing ones) [12]. For adequate perfusion and cellular viability, tissue‐engineered scaffolds must maintain cells within a diffusion limit within 200 μm from a functional capillary [16]. Insufficient vascularisation in these products can lead to ischaemia‐induced necrosis and, later on, implant failure. Therefore, various strategies, such as pre‐vascularising constructs or incorporating growth factors, are being explored to improve construct viability [17].

One strategy for designing pro‐angiogenic scaffolds involves the use of proteins such as fibrin, due to their cell‐binding sites that specifically facilitate EC adhesion [17]. Fibrin, a highly angiogenic plasma protein, is formed through the enzymatic conversion of fibrinogen by thrombin [18]. This polymerisation process is further influenced by other biological co‐factors such as calcium ions (*e.g.,* Ca^2+^) and factor XIIIa, which stabilise fibrin *via* covalent crosslinking [19]. Due to its crucial role in wound healing, tissue remodelling, haemostasis, and angiogenesis, fibrin provides a bioactive matrix that facilitates cell attachment, migration [20], and growth factor binding through specific receptors [21]. Consequently, fibrin is considered to be a promising biomaterial for tissue engineering applications, offering excellent biocompatibility, biodegradability, and the capacity to support endothelial network formation [22,23]. Recent studies have highlighted the potential of fibrin‐based scaffolds in soft tissue engineering applications, including skin [24], cartilage [25], and nerve tissues [26]. Moreover, these scaffolds have been associated with enhanced collagen fibre formation [24], support for cell differentiation [25], and accelerated tissue healing [24,26].

In comparison to other biopolymers, fibrin stands out for its exceptional properties, especially its pro-angiogenic potential [27]. While collagen I is the major constituent of the ECM, it does not possess the intrinsic angiogenic properties that make fibrin particularly effective in promoting vascular formation [28]. Studies report that fibrin is more effective in inducing angiogenesis, both *in vitro* [29] and *in vivo,* and that the two may act in synergy [30]. In contrast, gelatine, another component of ECM, poses challenges due to its poor thermostability, as it shifts from solid to gel with temperature changes. As such, it requires either physical or chemical crosslinking, which often leads to poor reproducibility and limited translation from *in vitro* studies to therapeutic applications [31].

Fibrin has also been used in emerging technologies such as 3D bioprinting, opening avenues for exciting applications. Its popularity as a bioink component is not only due to its biological properties, but also from its favourable viscoelastic characteristics and the ability to be readily crosslinked with thrombin before, during, or after printing [32,33]. Although fibrin is inherently soft and exhibits low mechanical strength, its fibrinogen concentration can be adjusted to enhance its mechanical properties [32]. Fibrin is also uniquely extensible [34], with individual fibres capable of stretching up to 200 times their original length [35]. It has also been used to create organ-on-a-chip models of vascularised tissues, demonstrating the strong potential of fibrin for developing physiologically relevant and translational systems [36].

Currently, commercial fibrin‐based products are limited to fibrin sealants, which are biodegradable materials that form physiologically stable clots for wound healing applications [37]. These sealants have demonstrated clinical success in enhancing wound healing, reducing bleeding, shortening post‐operative recovery and pain, and reducing the length of hospitalisation [38]. Despite fibrin's remarkable potential, fibrin has yet to achieve commercial use as scaffolds in tissue engineering, unlike the widely available collagen‐based alternatives. This is largely due to the inherent material limitations of fibrin, including its low mechanical strength, rapid enzymatic degradation, dense microstructure in gel form, and high shrinkage rate, which pose significant challenges for scaffold development [24,38]. Variations in scaffold design, composition, and fabrication techniques can significantly change the mechanical, biological, and angiogenic properties of fibrin scaffolds, thereby impacting microvascular network formation [39,40].

While existing systematic reviews (SRs) have focused on broader aspects of tissue engineering, a focused analysis of the evidence concerning the formulation and application of fibrin‐based scaffolds in soft tissue vascularisation remains lacking. A comprehensive understanding of how these factors influence tube formation, network formation, or EC migration is crucial for creating models that enhance soft tissue regeneration.

This SR aims to address this need by critically examining the current literature on fibrin‐based soft tissue scaffolds for tissue engineering, with a specific focus on their capacity to support endothelial network formation. The primary objective of this SR is to assess the efficacy of fibrin or fibrinogen‐based scaffolds, jointly referred to from here on as ‘fibrin scaffolds’, in promoting angiogenesis, including microvascular network formation, in soft tissues. This will be achieved by comparing studies that evaluated fibrin scaffolds (intervention) against control groups lacking such scaffolds (comparator), where the primary outcome is the formation of new blood vessels (outcome) in various soft tissue types (population).

We hypothesise that fibrin scaffolds facilitate angiogenesis, including microvascular network formation, and enhance EC migration in soft tissue regeneration, contributing to improved angiogenesis in both *in vitro* and *in vivo* studies. Accordingly, this SR seeks to answer the following research question: ‘How do variations in fibrin scaffold design and composition affect endothelial formation and migration processes in soft tissue models, in both *in vitro* and *in vivo* settings?’. By evaluating these factors, this review aims to deliver impactful insights into the design principles of fibrin‐based scaffolds, advancing their application in promoting vascularisation and improving outcomes in soft tissue regeneration.

## 2 Methodology

### 2.1 Eligibility criteria & study design

The eligibility criteria for this SR were carefully defined to ensure the inclusion of relevant studies and the exclusion of those that did not meet the predefined parameters. The study characteristics used to determine eligibility aligned with a modified Population, Intervention, Comparison, and Outcome (PICO) framework, as outlined below.

**Population:** Eligible studies included those employing *in vitro*, *ex vivo*, and/or *in vivo* models (male or female animals) of (micro)vasculature in the context of soft tissue regeneration. The inclusion criteria encompassed studies that incorporated clinical, simulation or modelling data in the same article, as long as experimental data from *in vitro*, *ex vivo* or *in vivo* models were generated. Studies were excluded if they were exclusively clinical studies, simulations (*e.g.,* computational), or modelling (*e.g.,* mathematical) that did not report any experimental data on *in vitro*, *ex vivo* or *in vivo* models of (micro)vascularised soft tissue.

**Intervention/Exposure:** The primary intervention of interest was the application of fibrin‐based soft tissue scaffolds to achieve (a) tube or tube‐like structure formation, (b) network or interconnected tube formation, and/or (c) EC migration. From here onwards, these three outcomes will collectively be referred to as ‘endothelial formation and migration processes (EFMPs)’. Specifically, the inclusion criteria encompassed:

• Tissue engineering scaffolds/constructs containing fibrin (or fibrinogen) in their formulation, either alone or combined with other materials, in any concentration or composition.
• Scaffolds either pre‐embedded with cells or acellular.
• Any type of manufactured scaffold (*e.g.,* hydrogel, mesh).
• Fibrinogen from any source (*e.g.,* commercial) or species (*e.g.,* human, bovine).
• Publications reporting data on scaffold design (*e.g.,* formulation, manufactured object type).
• Publications reporting outcomes related to at least one of the three endothelial formation and migration processes (*i.e.,* tube formation, microvascular network formation, EC migration).
• Single or repeated use of the scaffold.
• Any study type (*e.g., in vitro*, *ex vivo*, *in vivo*).

The review excluded studies that only used autologous or allogeneic grafts or materials, including platelet‐rich fibrin (PRF), without the development of a tissue engineering scaffold.

**Comparators/Control:** Eligible control groups included:

• Non‐fibrin‐based scaffolds or controls using other materials (*e.g.,* collagen, gelatine, synthetic polymers).
• Non‐exposed control groups comprising *in vitro* or *in vivo* models where no scaffold was applied.
• Groups subjected to placebo or “sham” procedures.

No specific exclusion criteria were applied for comparators.

**Study Design:** The review included publications reporting *in vitro*, *ex vivo* and/or *in vivo* studies, either alone or alongside other study types. Excluded studies were those that did not report experimental data (*e.g.,* reviews, mathematical or computational simulations without experimental data). This SR was designed following Preferred Reporting Items for Systematic Reviews and Meta‐analysis (PRISMA) guidelines, and the protocol was registered with the Open Science Framework (OSF^2^) and the International Prospective Register of Systematic Reviews (PROSPERO^3^).

**Outcomes:** The primary outcome of interest was whether theEFMPs were successful or unsuccessful. Secondary outcomes included data on evaluations of EFMPs, including types of assays and techniques. Studies needed to report quantitative and/or qualitative data on these outcomes. Assessments could include measures such as EC migration distance, microvascular network area coverage, or dichotomous outcomes like successful versus failed network formation.

Studies were excluded if they did not measure or report outcomes related to angiogenesis, such as tube formation, microvascular network formation, and/or EC migration (*i.e.,* EFMPs) within fibrin‐based scaffolds in soft tissue models. Additionally, any study lacking quantitative or qualitative data on angiogenesis or endothelial network migration was excluded.

**Exclusion Criteria:** All records indexed in the selected databases until 28 October 2024 were considered. Exclusion criteria, in the order these were assessed, included:

1. Retracted publications.
2. Non‐peer‐reviewed publications.
3. Non‐original research articles (*e.g.,* reviews, editorials).
4. Articles not published in English.
5. Studies do not include experimental data and/or outcomes of EFMPs.
6. Studies do not contain fibrin‐based scaffolds.
7. Studies do not focus on soft tissue applications.
8. Studies with exclusive use of autologous or allogeneic grafts or materials.

### 2.2 Search strategy

A comprehensive and systematic search was conducted across three databases (PubMed,^4^ OVID MEDLINE^5^, and Scopus^6^) on 28 October 2024. The search combined four key elements: soft tissues; tissue engineering scaffolds; the use of fibrin or fibrinogen; and EFMPs (Figure S1), using tailored queries for each database to match their specific data extraction strategies. No restrictions were placed on publication date, and, when possible, filters were applied to narrow down the search results to original research articles only. The exact search terms and query structures used, as well as the exact number of results obtained on the date of the databases search, are available in Appendix I. MeSH terms were utilised in PubMed to improve recall and precision [41]. The search strategy was designed adhering to PRISMA guidelines and was reviewed by an expert subject librarian (Bodleian Library, University of Oxford) to independently validate the search.

### 2.3 Study selection process

A two‐stage screening process was employed to ensure a thorough and rigorous evaluation of all identified articles, consisting of (a) title & abstract, and (b) full‐text screening.

**Title** & **Abstract Screening (1**^st^ **Screening Round):** Three reviewers independently screened titles and abstracts to assess eligibility based on the inclusion criteria. Any disagreements were resolved through discussion until consensus was reached. Records marked as ‘maybe’ by at least one reviewer were also discussed collectively. The articles considered to be relevant by all reviewers proceeded to the second round for full‐text assessment.

**Full‐Text Screening (2**^nd^ **Screening Round):** In the second round, the three reviewers independently assessed the full text of potentially eligible studies. Articles were excluded if they met any of the exclusion criteria, applied in the order listed in Section 2.1. As in the first round, disagreements and records marked as ‘maybe’ were resolved through discussion until full agreement was achieved.

**Screening Procedure:** Screening was conducted independently and in parallel by three reviewers using Rayyan software, with blind mode enabled to minimise bias. The use of blind mode and independent assessment by three reviewers enhanced objectivity. Once all reviewers had made their inclusion or exclusion decisions, blind mode was disabled. Any conflicts or records marked as ‘maybe’ were resolved through joint discussion. Final decisions were recorded in Rayyan.

### 2.4 Methods for data extraction

A structured table specifying the planned data entities and their requirements was prepared in advance. All three reviewers participated in detailed discussions to agree on the definitions and requirements for each entity. The included studies were then divided among the reviewers, with each reviewer independently extracting data for their assigned articles and populating the table accordingly.

For each entry, the extracted data were subsequently validated by the other two reviewers. If issues arose regarding a specific entity, reviewers consulted each other to examine the relevant article or data point. Upon completion of data extraction, all reviewers collectively reviewed the finalised table to ensure consistency and accuracy.

Data were extracted manually by the research team without contacting original study investigators for clarification. For studies reporting multiple scaffolds, only data from those meeting the inclusion criteria were extracted; data related to excluded scaffold types were omitted accordingly. All reported results relevant to the predefined outcome domains were extracted from each study. Where multiple measurements were provided for the same outcome (*e.g.,* several assays for vascularisation), all were recorded as independent data entries. Time frames of outcome measurements (*e.g.,* hours, days, or weeks post‐intervention) were not extracted, as reporting across studies was inconsistent. Upon completion, the full dataset was reviewed collectively by all three reviewers to confirm completeness and internal consistency. Data was extracted on the following categories:

1. Study Design
  (a) Study type(s) reported in the publication (*e.g., in vitro*, *ex vivo*).
  (b) Source of fibrinogen (*e.g.,* commercial vs. extracted as part of the research; manufacturer; species and country of origin).
  (c) Soft tissue replicated or studied (*e.g.,* skeletal muscle, skin).
2. Model Used
  (a) For *in vitro* studies only: Cell line(s), including origin (*e.g.,* commercial, isolated) and species (*e.g.,* human, rat).
  (b) For *in vivo* studies only: *In vivo* model used, including *taxa*, species, and sex of specimen.
3. Intervention of Interest
  (a) Scaffold formulation (*e.g.,* fibrinogen concentration and source; types of coagulant(s) and/or crosslinker(s); and use of additional materials (*e.g.,* chemicals, growth factors, polymers).
  (b) Type & features of the manufactured object (*e.g.,* mesh, gel).
4. Primary Outcome
  (a) Overall success or failure of EFMPs.
5. Secondary Outcomes
  (a) Quantitative and/or qualitative evaluation of EFMPs:
  i. Continuous measures (*e.g.,* average vessel length, area covered by newly formed in or migrated vessels into the scaffold).
  j. Dichotomous assessments (*e.g.,* success or failure of EFMPs).
6. Bibliographical data (*e.g.,* author, year, PMID, DOI).

**Approach to Masking, Reliability, Reconciliation, and Justification:** Masking was not employed during the data extraction process. To ensure reliability and objectivity, a multi‐reviewer approach was adopted: each reviewer independently extracted data from their assigned studies, after which all three reviewers collectively reviewed the extraction table to ensure consistency and accuracy. Any discrepancies were resolved through group discussion, with each reviewer presenting their rationale until consensus was reached. This process was designed to maximise accuracy and efficiency in data extraction, ensuring that the review is based on reliable, transparent, and reproducible methods.

### 2.5 Strategy for data synthesis

The synthesis focused on key groupings, including variations in fibrin scaffold composition, manufactured object type used for the scaffolds, cell pre‐embedding or acellular scaffolds, and relevant study design characteristics. Studies were considered eligible for qualitative synthesis if they provided sufficient descriptive data on the intervention (*e.g.,* fibrin scaffold characteristics, fabrication method) and reported qualitative and/or quantitative data on the outcomes (*e.g.,* network formation). A meta‐analysis was not conducted due to the considerable heterogeneity in study designs, scaffold formulations, and outcomes.

**Synthesis Procedure Justification:** Given the diversity of methodologies and outcome measures, as well as the heterogeneity of studies, a structured narrative synthesis was employed to ensure a comprehensive and nuanced interpretation of the evidence. Extracted data were organised into summary tables, which were used to create figures to facilitate comparison across studies.

The synthesis was structured around the following two core outcomes: **(1) Intervention:** Characteristics of the intervention vehicles (*e.g.,* study type, manufactured object type); and **(2) Outcome:** Evidence of EFMPs. For each included study, an overview of the intervention and its outcomes was provided.

**Subgroup Analyses:** Data were analysed both individually and within one of the two following subgroups: **(1) Constructs:** Scaffolds embedded with cells, where the EFMPs were evaluated; and **(2) Scaffolds:** Acellular scaffolds usually implanted in *in vivo* models, where endothelial migration and subsequent tube, vessel or network formation were assessed. An additional subgroup was created, separately from the scaffold‐construct divide, compiling all muscle‐focused studies: cardiac, smooth, and skeletal.

**Data Presentation and Visualisation:** Summary tables and figures were used to present key findings, including:

1. PRISMA flow chart illustrating the study selection process.
2. Overview of the corpus (*e.g.,* number of records per year, soft tissue types, study types, cell lines, and *in vivo* models).
3. Fibrin source and scaffold composition paired with a successful or unsuccessful outcome of EFMPs.
4. Types of assays used to evaluate EFMPs.
5. Subgroup analysis focusing on muscle tissue studies.

**Synthesis Process and Reliability:** The synthesis was conducted collaboratively by three reviewers to ensure comprehensive and balanced conclusions. Any disagreements or uncertainties were resolved through discussion until consensus was achieved. Data were extracted and reported in the original units provided by study authors to preserve fidelity to source data and avoid unverifiable assumptions.

**Data Management and Sharing:** All synthesis results were presented within the manuscript. Additionally, the database compiling extracted data from included articles was made openly available *via* GitHub.

Additional Considerations:

1. **Missing Data:** Only data reported in the studies was included.
2 **Data Conversion & Validation:** Not applicable.
3 **Criteria for Conclusions:** Not applicable.
4. **Synthesist Blinding:** Not applicable.
5. **Sensitivity Analyses:** None were planned.
6 **Statistical Analyses:** Not applicable.
7. **Publication Bias Analyses:** Risk of Bias (RoB) was evaluated using SYRCLE's (Systematic Review Centre for Laboratory Animal Experimentation) RoB tool.
8. **Quality Assessment:** A modified version of the CAMARADES (Collaborative Approach to Meta‐Analysis and Review of Animal Data from Experimental Studies) checklist was used.

### 2.6 Risk of bias assessment

The RoB on studies focused on muscle research was assessed using SYRCLE's RoB tool, which was developed as an adaptation of the Cochrane RoB tool but specifically tailored to address sources of bias unique to *in vivo* intervention studies [[42], [43], [44]]. SYRCLE's RoB tool evaluates six types of bias (selection, performance, detection, attrition, reporting, and other biases) across ten domains; including sequence generation, allocation concealment, random housing, blinding, incomplete outcome data, and selective outcome reporting [42,45].

Three independent reviewers conducted the RoB assessment in parallel, with blind mode activated to minimise bias during initial evaluation. For each study, reviewers independently answered ten questions^7^ with ‘Yes’, ‘No’, or ‘Unclear’, strictly based on the information reported in each article. After completing their independent assessments using the SYRCLE tool, the blind was lifted and all reviewers convened to discuss and resolve any disagreements through group discussion, ensuring consensus for each checklist item.

Study investigators were not contacted for additional methodological information, and no automation tools were used in the RoB assessment. The results of the RoB assessment informed the interpretation of findings but were not used as a basis for excluding studies from the review.

### 2.7 Quality assessment

Study quality on articles focused on muscle tissue was evaluated using a modified CAMARADES checklist. The CAMARADES checklist is a reporting quality tool designed for preclinical *in vivo* studies, comprising up to ten items that assess methodological features such as randomisation, blinding, sample size calculation, and compliance with animal welfare regulations [[46], [47], [48]]. In this SR, the checklist [49] was adapted by removing two items “Blinded induction of ischaemia” and “Statement of control of temperature” as they were not applicable for this SR, resulting in an eight‐item assessment.^8^

Each article was evaluated across these eight criteria, with three reviewers independently assigning ‘Yes’, ‘No’, or ‘Unclear’ for each item. One point was awarded for each ‘Yes’ response, resulting in a maximum possible score of eight. No points were awarded for ‘No’ or ‘Unclear’ responses. Studies scoring fewer than four points were classified as having “poor methodological quality”, while those with four or more points were considered to have “good methodological quality”.

The quality assessment process mirrored the RoB procedure: three independent reviewers completed the CAMARADES checklist in parallel and, following independent evaluation, resolved any discrepancies through group discussion to reach consensus on each item. Study investigators were not contacted for clarification, and no automation tools were used in the quality assessment.

### 2.8 Software

The following tools were used in this SR: Rayyan [50] (online version) for blinded screening and organising studies; Mendeley (online version) for managing citations and references; Microsoft Excel (version 2503) for manual data extraction and analysis; GraphPad PRISM (version 10.4.2), *RAWGraphs* [51] (online version), and *BioRender* [agreement numbers: LU28EG2MJP, BQ28CLLTGW & HO28PQNBZQ] for creating figures and illustrations; Microsoft Word (version 2503) for note‐taking and manuscript writing; and Overleaf (online version) for manuscript preparation.

## 3 Results

### 3.1 Study selection

**Fig. 1:**
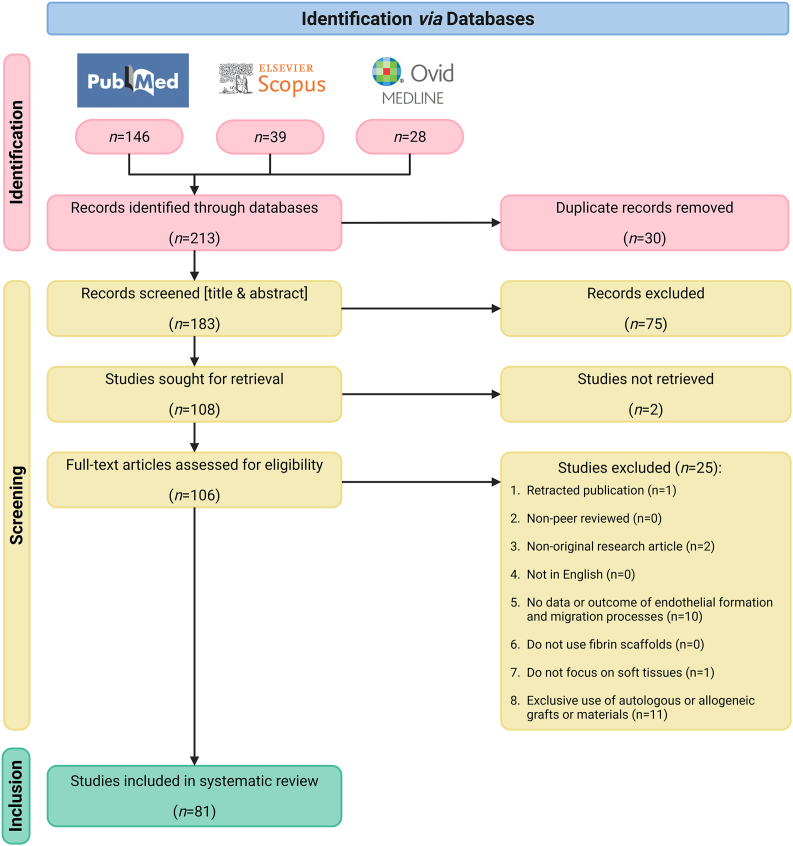
**PRISMA Flow Diagram of the Study Selection Process**, summarising the number of records identified, screened, excluded, and included at each stage of the systematic review. The diagram provides a detailed account of study progression through the phases of identification, eligibility assessment, and final inclusion.

The study selection process was conducted in accordance with PRISMA guidelines (Fig. 1), encompassing the identification, screening, and inclusion stages to ensure a transparent and reproducible review. The process began with the identification of 146 records from PubMed, 39 from Scopus, and 28 from OVID (Figure S1). After removing 30 duplicates, 183 records remained for screening by title and abstract (Figure S2A). At this stage, 75 records were excluded based on the predefined criteria and the remaining 108 articles were sought for retrieval for full‐text assessment (Figure S2B). Two articles could not be retrieved. Following a detailed evaluation based on the exclusion criteria, 25 articles were excluded (Table S1). The details of the 81 studies included in the final corpus are available in Table S2.

### 3.2 Overview of the corpus

**Fig. 2:**
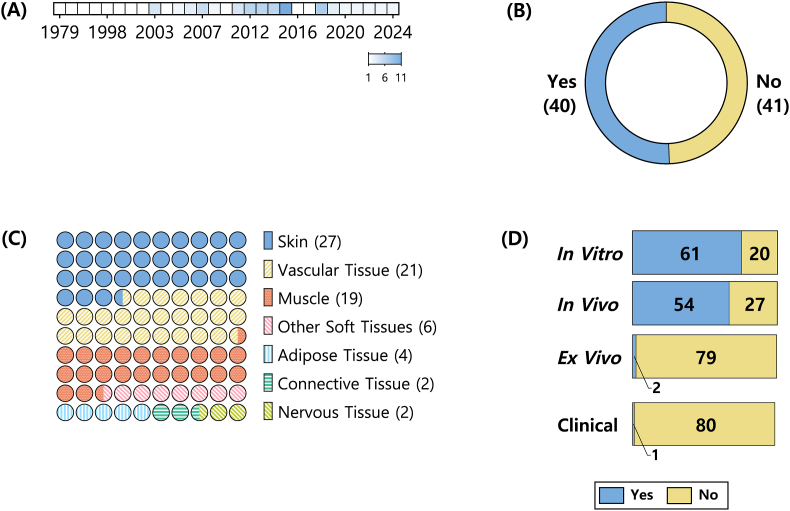
**Overview of the Corpus of Included Articles in the Systematic Review (n = 81)**, depicting (A) number of publications by year; (B) number of articles where the main aim of the study either included (49.38 %) or not (50.62 %) endothelial formation or migration processes (EFMPs); (C) distribution of soft tissue types studied; and (D) classification of studies by type, including *in vitro*, *in vivo*, *ex vivo*, and clinical studies.

The 81 articles [[52], [53], [54], [55], [56], [57], [58], [59], [60], [61], [62], [63], [64], [65], [66], [67], [68], [69], [70], [71], [72], [73], [74], [75], [76], [77], [78], [79], [80], [81], [82], [83], [84], [85], [86], [87], [88], [89], [90], [91], [92], [93], [94], [95], [96], [97], [98], [99], [100], [101], [102], [103], [104], [105], [106], [107], [108], [109], [110], [111], [112], [113], [114], [115], [116], [117], [118], [119], [120], [121], [122], [123], [124], [125], [126], [127], [128], [129], [130], [131], [132]] included in the final corpus provide an overview of the current literature on fibrin‐based soft tissue scaffolds for tissue engineering, with a particular emphasis on their capacity to support EFMPs. By analysing the corpus, we can observe a few trends, for instance publication frequency of scientific studies, study aims, soft tissue focus, and study types used (Fig. 2).

The publication dates span from 1979 to 2024 (Fig. 2A), with the database search conducted on 28 October 2024. Besides the article published in 1979, no articles were published until 1994. From then on, there is a slight increase in the trend, with notable peaks in 2015 (11 articles), as well as in 2018 (n = 7) and consistently between 2012 and 2014 (6 articles per year).

Focusing on the study aims (Figs. 2B), 40 articles identified endothelial formation and migration as a central aim, thus providing focused analyses into the role of fibrin scaffolds in promoting vascularisation. The remaining 41 studies reported network formation as a secondary outcome, often within broader investigations of soft tissue repair or regeneration.

The corpus covers a range of soft tissue targets (Fig. 2C). Skin was the most frequently studied tissue, with 27 articles, followed by vascular tissue, including both microvasculature and macrovasculature, in 21 studies. Muscle tissues, encompassing both cardiac and skeletal muscle, were the focus of 19 articles. Adipose tissue was addressed in four studies, connective tissue (including dental pulp) in two, and nervous tissue (including spinal cord) in another two. Six articles addressed soft tissue regeneration in general, without specifying a particular tissue type. This distribution highlights the broad range of fibrin scaffold applications across various soft tissue engineering contexts.

A variety of experimental approaches were employed across the included studies (Fig. 2D; Table S3). *In vitro* methods were used in 61 articles, while 54 studies included *in vivo* experiments. *Ex vivo* approaches were reported in two articles, and one study included clinical data. No studies in the corpus used computational modelling. Notably, 35 articles employed more than one study type (Dataset available at https://github.com/carla-fuenteslopez/soft-tissue-fibrin-sr), most commonly a combination of *in vitro* and *in vivo* methods. Two articles used three different study types, both of which included *ex vivo* models.

### 3.3 Fibrin scaffold composition: influence on successful endothelial formation and migration processes (EFMPs)

**Fig. 3:**
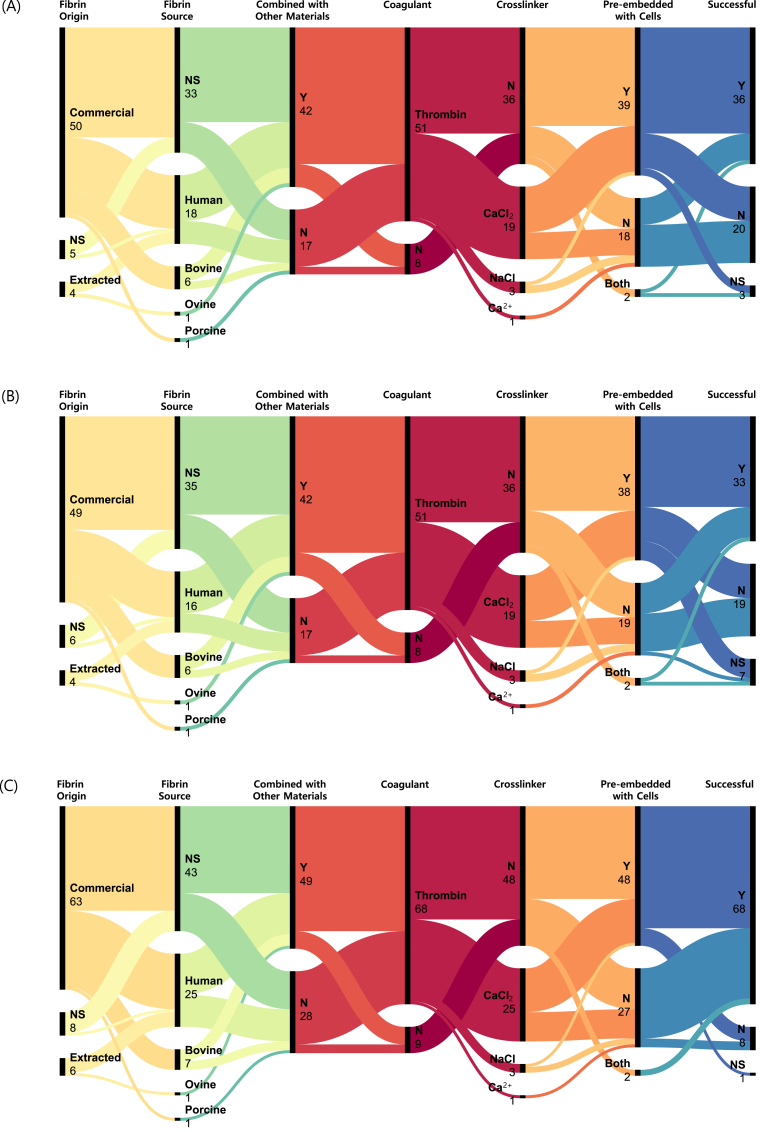
**Impact of ScaffoldFormulation on Angiogenic Outcomes**, illustrating the relationships between scaffold formulation parameters (fibrin origin, source, combination with other materials, cross-linker, coagulant, and cell pre-embedding) and the resulting successful endothelial formation and migration processes (EFMPs), by number of scaffolds. The impact of the scaffold formulation is split into: (A) tube formation, (B) networks, or (C) endothelial cell (EC) migration. Data were aggregated from the included studies, showing the frequency of each parameter and its association with successful angiogenic outcomes. N: No; NS: Not Specified; Y: Yes. Please refer to the Supplementary Materials (Tables S5, S6, S7, S8, S9 & S10 and Figure S3) for further details.

The composition and formulation of fibrin scaffolds in the included studies demonstrated a wide variety of approaches (Fig. 3; Table S4). The impact of each design parameter on the success of EFMPs was also evaluated (Figure S3).

Fibrin scaffolds have emerged as a widely investigated platform for tissue engineering, particularly in applications requiring vascularisation. A comprehensive evaluation of the included studies reveals nuanced relationships between fibrin origin, source material, combination with other materials, use of cross‐linking agents, coagulants, cell pre‐embedding, and the resulting success or failure of tube (Fig. 3A) or network (Fig. 3B) formation, or of endothelial migration (Fig. 3C).

The success of the fibrin scaffolds used in the studies included in this SR in promoting EFMPs was aggregated as a variable to facilitate the identification of the key parameters impacting the reported outcomes. This measure was calculated as the success rate by dividing the number of successful cases by the sum of successful cases and unsuccessful cases (Equation (1)). The number of scaffolds not used for that particular application or that did not report whether it was successful or not, were not included in the calculation. Success rate calculations only included those scaffolds that contained fibrin.

(Eq. 1) $$Success\;Rate\;\left(\%\right)=\frac{Successful\;Scaffolds\;\left(n\right)}{Successful\;Scaffolds\;\left(n\right)+Unsuccessful\;Scaffolds\;\left(n\right)}$$

#### 3.3.1 Fibrinogen, coagulants, and crosslinkers

Considerable variation was observed in the use and reporting of fibrinogen, coagulants, and crosslinkers for scaffold fabrication (Fig. 3 & Table S5), which may, in turn, influence the expected outcomes related to angiogenesis. For example, fibrinogen concentrations were reported using different units (*e.g.,* mg/ml, wt%, v/v% or μl) or used at unknown concentrations, with some studies specifying stock solution concentrations and others reporting final scaffold concentrations. In 12 cases, the stage at which the concentration applied was unclear, and 14 articles did not report fibrinogen concentrations at all. This inconsistency hinders direct comparison and reproducibility.

The majority of studies (72 of 81) reported the use of a coagulant, and all of them used thrombin. Nine studies did not explicitly state whether a coagulant was used. Crosslinking agents were used in 32 scaffolds, with four different crosslinkers used across the corpus. *CaCl*_*2*_ was the most common crosslinking agent, used in 27 scaffolds, always in conjunction with thrombin to enhance network stability. Other crosslinkers used were *NaCl* (n = 3), Ca^2+^ (n = 1), and *MgCl*_*2*_ (n = 1).

##### 3.3.1.1 Fibrinogen origin and source

The majority of studies that specified the origin of fibrinogen (74 scaffolds) employed commercially available fibrinogen (91.89 % of scaffolds), while extracted fibrin was significantly less used (8.11 %). In 8 scaffolds (9.76 %), the origin of the fibrinogen or fibrin was not specified.

Of the studies that reported the source (37 scaffolds; 45.12 %), human fibrinogen was most employed (75.68 % of scaffolds) followed by bovine (18.92 %), porcine and ovine (2.70 % each). A significant portion of scaffolds (54.88 %) did not report the fibrinogen source used.

When evaluating tube and network formation, scaffolds made with commercial or extracted fibrinogen showed similar success rates. For tube formation, 33 out of 48 scaffolds made with commercial fibrinogen were successful (68.75 %), compared to 2 out of 3 scaffolds with extracted fibrinogen (66.67 %). Likewise, network formation was successful in 29 of 43 commercial scaffolds (67.44 %) and 2 of 3 extracted scaffolds (66.67 %). However, a different trend emerged when assessing endothelial migration into the scaffolds. Success rates were higher with commercial fibrinogen (33 out of 62 scaffolds; 53.23 %) than with extracted fibrinogen (2 out of 6 scaffolds; 33.33 %).

As for the choice of fibrinogen origin, opting for human over bovine origin was associated with a higher success rate when evaluating tube (87.50 % vs. 66.67 %) and network formation (84.62 % vs. 66.67 %), but the reverse occurred when focusing on endothelial migration (91.67 % vs. 100 %). This is a small difference in migration success, especially considering that human fibrinogen was used 2–3 times more often than bovine. Due to limited data points, no meaningful conclusions can be withdrawn regarding ovine (n = 1; unsuccessful) and porcine (n = 1; successful) fibrinogen.

##### 3.3.1.2 Crosslinkers

A total of 31 scaffolds (37.81 %) reported the use of a crosslinker (*i.e.,* crosslinker reagents; agents that enable intermolecular or intramolecular interactions of two or more molecules by covalent, ionic, or hydrogen bonding) and specified the one used: *CaCl*_*2*_ (87.10 %), *NaCl* (9.68 %) and *Ca*^*2+*^ (3.23 %). *Ca*^*2+*^ is not integrated into the subset of *CaCl*_*2*_ and is treated separately in the analysis to distinguish the direct use of the active ionic form, as explicitly reported by the authors [88], from the use of *CaCl*_*2*_, the compound employed by most studies in this corpus.

For tube formation, the absence of a reported crosslinker showed the highest success rate (70.59 % success rate of 34 scaffolds), closely followed by the use of *CaCl*_*2*_ as a crosslinker (55.56 % of 18). For network formation, a similar trend was observed, with studies not reporting crosslinker use showing the highest success (67.74 % of 21), followed by *CaCl*_*2*_ (58.82 % of 17). However, in the context of endothelial migration, scaffolds incorporating *CaCl*_*2*_ achieved higher success rate (87.50 %, n = 24), closely comparable to those not reporting crosslinker use (89.58 %, n = 43). However, these findings are based on small and imbalanced group sizes, so the evidence should be interpreted with caution.

The two other crosslinkers, *NaCl* (used in 3 scaffolds) and *Ca*^*2+*^ (n = 1) were used in considerably fewer scaffolds. Given the limited use of *NaCl* and *Ca*^*2+*^, the associated success rates (*NaCl*: 33.33 % in tube and network formation and 100 % in migration; and *Ca*^*2+*^: 100 % in all three processes) should be interpreted with caution, as they may not be representative of broader trends.

##### 3.3.1.3 Coagulants

Regarding coagulants (*i.e.,* agents that cause a liquid to coagulate), 73 scaffolds (89.02 %) reported the use of a coagulant. All of them opted for thrombin, though at different concentrations; however, its impact on angiogenic outcomes requires nuanced consideration. For tube formation, thrombin use yielded a 65.31 % success rate (49 scaffolds), which was slightly higher than the 57.14 % reported for studies not reporting coagulant use (n = 7). Network formation demonstrated a similar trend, with thrombin showing a 64.44 % success rate (n = 45) versus 57.14 % for studies not reporting coagulant use (n = 7). In contrast, endothelial migration exhibited a lower success rate when using thrombin (88.06 % success rate in 67 scaffolds) compared to studies that did not report the use of coagulant (100 % success rate in 9 scaffolds).

#### 3.3.2 Inclusion of other materials in the formulation

Beyond fibrin, coagulants, and crosslinkers, many studies incorporated additional materials into the scaffold formulation (Fig. 3), which were classified into five categories: polymers (natural and synthetic); growth factors, peptides, amino acids, and enzymes (GF/P/AA/E); cell media and solutions; chemicals; and other materials.

GF/P/AA/E were frequently used in combination with several scaffolds incorporating multiple factors, whereas polymers, chemicals, and cell media were typically used individually (Figure S4A). Based on the frequency of use across the included studies (Figure S4B), GF/P/AA/E were the most common (34 articles), followed by polymers (n = 32), cell media and solutions (n = 16), other materials (n = 14), and chemicals (n = 4).

A total of 32 articles used at least one type of polymer (Table S6), with five articles using two and one article using three types. Overall, 19 different polymers were used in the corpus, with the most common being collagen (7 scaffolds), polyethylene oxide (PEO; n = 4) and alginate, fibronectin, heparin, polyethylene glycol (PEG), and succinimidyl glutarate‐modified PEG (n = 3 each).

GF/P/AA/E were incorporated in 34 articles, with 26 studies using two or more different types (Table S7). One scaffold even used 10 different GF/P/AA/E, while two others used five, and a further two used four. A total of 37 different GF/P/AA/E were used, with aprotinin (19 scaffolds), basic Fibroblast Growth Factor (bFGF; n = 11), Factor XIII (n = 9, including 3 scaffolds using Factor XIIIa), and Vascular Endothelial Growth Factor (VEGF; n = 8) being the most frequent.

Cell culture media and solutions were used in 16 scaffolds (Table S8), with Dulbecco's Modified Eagle Medium (DMEM), Foetal Bovine Serum (FBS), Medium 199 (M199), and *NaCl* being the most frequent (2 scaffolds each). In total, 12 different cell media and solutions were identified across the included studies. Only four scaffolds used chemicals (Table S9), specifically ε‐aminocaproic acid; tranexamic acid; trisodium citrate, and Triton X (each in 1 scaffold). Finally, 14 scaffolds incorporated materials classified as “other” (Table S10), which included *Cytodex* beads, *Fe*_*3*_*O*_*4*_ magnetic nanoparticles, and poly(lactic‐co‐glycolic acid) (PLGA) nanospheres.

Within the studies included in this SR, 52 scaffolds (63.41 %) used other materials in the scaffold formulation besides fibrin, coagulants, and crosslinkers. Frequently used materials in combination with fibrin were grouped into five overarching categories, including: growth factors such as VEGF or bFGF, and polymers like PEG. While the presence of these additional materials influenced EFMPs outcomes; the diversity of materials used, along with variability in their concentrations, and combinations, precludes definitive conclusions about the effects of individual additives.

The addition of other materials also played a role in determining success. Of the scaffolds that reported successful or unsuccessful outcomes, composite scaffolds were associated with lower success rates than the scaffolds not including additional materials besides fibrinogen, coagulants, and crosslinkers in their formulations. This occurred in the three scenarios: tube formation (61.54 % vs. 70.59 %); network formation (58.33 % vs. 75.00 %); and endothelial migration (85.42 % vs. 96.43 %). However, it is worth noting that there were also differences in the number of scaffolds used in each case, with composite scaffolds being double the amount of the other scaffolds, potentially introducing bias in comparative analyses.

#### 3.3.3 Cell pre‐embedded vs acellular scaffolds

Of the scaffolds analysed, 52 (65.82 %) were pre‐embedded with cells before experimental work, while the remaining 27 (34.18 %) were acellular (Fig. 3). In two cases, the same scaffold formulation was used independently as pre‐embedded and acellular, so each configuration was treated as an independent scaffold to ensure that the analysis captured the distinct impact of cell pre‐embedding on scaffold performance. Also, one scaffold was used with pre‐embedded cells to assess tube formation (not successful), and its acellular form for network formation and migration (successful in both cases).

Pre‐embedding cells into fibrin scaffolds demonstrated a generally positive impact on tube and network formation, with higher success rates of 76.32 % and 73.53 %, respectively, than when not pre‐embedded (success rates of 42.11 % and 47.37 %). In contrast, EC migration showed nearly identical success rates in both conditions, 89.80 % for pre‐embedded scaffolds and 89.66 % for acellular scaffolds.

Across the three EFMPs outcomes, the number of scaffolds incorporating pre‐embedded cells was consistently 1.7 to 2 times greater than the acellular scaffolds, with group sizes ranging from 19 to 49 scaffolds. These sample sizes support the reliability of conclusions for both pre‐embedded and acellular variations.

#### 3.3.4 Culture media supplementation

While scaffold formulation is critical, the cell culture environment also plays a significant role in the success of tissue‐engineered scaffolds. Most studies (72.84 %) used standard cell culture media without additional supplements. However, 22 publications reported the use of modified media (Table S11), with two studies testing two variations within the same study but using the same scaffold formulation. Supplements were typically added to stimulate EC growth and sprouting [107], enhance cell viability [91], prevent premature fibrin degradation [73,75,97,118], stimulate cell adhesion, migration and proliferation [84], and matrix remodelling by mimicking the *in vivo* environment and inducing vascularisation [115] and vessel formation [57,121,130].

Among the modified media formulations, the most common supplements were aprotinin (12 studies, concentrations: 0.15–200 U/ml), VEGF (n = 11, concentrations: 25–100 ng/ml or 0.1 %), and FGF, including variants such as bFGF and human recombinant‐bFGF, (n = 7, concentrations: 10–50 ng/ml or 0.4 %). Other additives included antibiotics (*e.g.,* penicillin, streptomycin), matrix modulators (*e.g.,* heparin, heparan), and sera (*e.g.,* foetal bovine/calf serum, human serum). As with other parameters, when reported, supplement concentrations varied considerably between studies.

### 3.4 Cell lines & *in vivo* models

#### 3.4.1 Cell lines

**Fig. 4:**
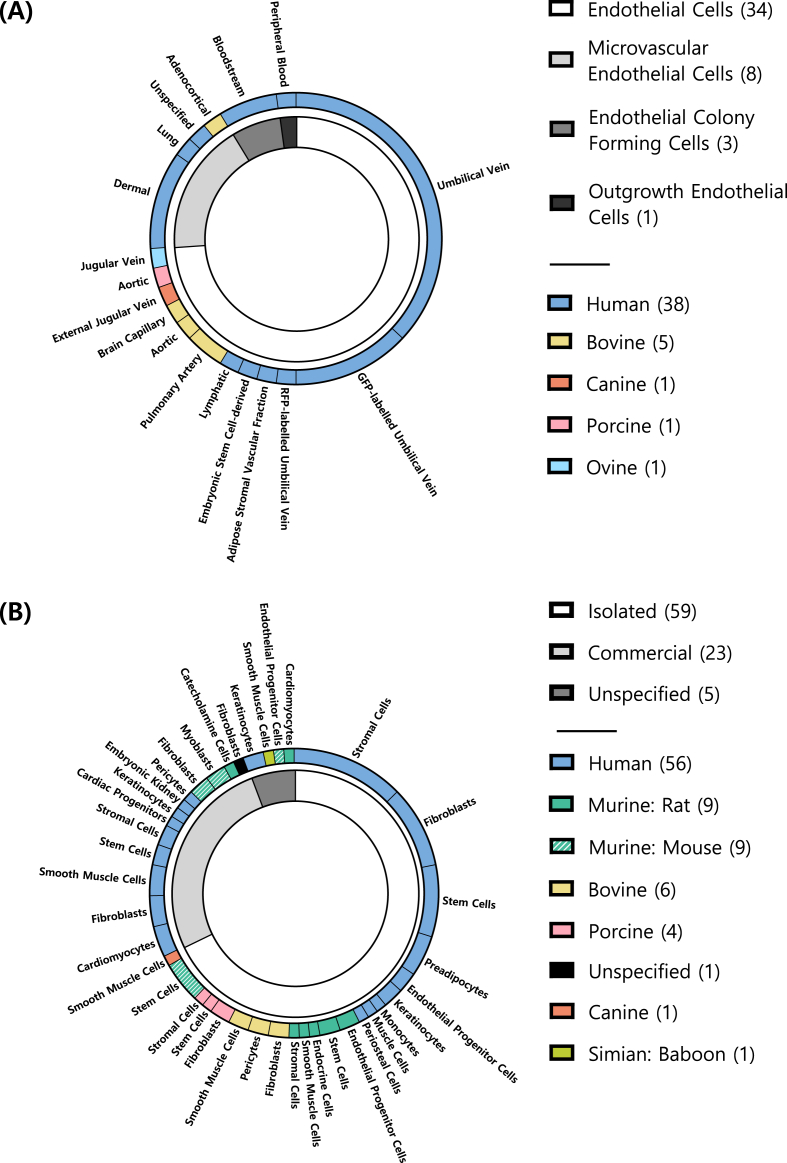
**Endothelial (EC) and Non-Endothelial Cell (Non-EC) Lines Used**, by number of scaffolds in which they were used. (A) ECs by type, species, and specific cell line name. (B) Non-EC lines used by source, species, and specific cell line name.

Cell line selection for tissue engineering scaffolds reflects the specific biological and experimental aims of each study. Here, we categorised the cell lines used in the 81 studies (Fig. 4) into two main groups: endothelial (Fig. 4A) and non‐endothelial (Fig. 4B). Of these studies, 40 (49.38 %) utilised either endothelial or non‐EC lines, 30 (37.04 %) incorporated both groups, and 11 studies (13.58 %) did not integrate any cell lines into the scaffolds. EC lines were employed in 39 studies (48.15 % of studies; 46 scaffolds), while non‐EC lines were used in 61 studies (75.31 % of studies; 87 scaffolds). The evaluation of endothelial formation and migration was dependent on the presence or absence of pre‐embedded ECs within the scaffold. When ECs were present, either alone or alongside non‐ECs, tube and network formation (including post‐differentiation) could be evaluated. This outcome may be attributed to the cells interacting with the matrix surrounding them, which not only provides physical support but also promotes cell adhesion, migration, and develops a functional network [133,134]. Conversely, when ECs were absent, only endothelial migration into the scaffold could occur. Notably, migration was also reported in some cases where ECs were pre‐embedded into the scaffolds.

Human‐derived cell lines dominated both groups, but sourcing strategies differed: endothelial lines were often obtained from commercial sources (20 of 46 scaffolds), whereas non‐ECs were mostly isolated from primary tissues as part of the study (57 of 87 scaffolds). Tissue origin also varied: EC lines commonly used umbilical vein (n = 42), while non‐ECs were often adipose‐derived (n = 21), reflecting distinct scaffold design priorities and biological targets across studies.

##### 3.4.1.1 Endothelial cell (EC) lines

A total of 22 different EC lines were used in 46 scaffolds across 40 studies (Fig. 4A). The EC lines used were classified into four groups: ECs (used in 34 scaffolds), microvascular ECs (n = 8), endothelial colony‐forming cells (n = 3), and outgrowth ECs (n = 1). Human‐derived cell lines were overwhelmingly dominant (n = 38), likely due to their greater translational relevance. The other cell lines, derived from bovine (n = 5), canine (n = 1), porcine (n = 1), and ovine (n = 1) origins, were used less frequently.

Commercial sources were common (20 scaffolds); while 18 scaffolds used isolated cells, five were gifts or derived from an existing cell line without specifying their origin (commercial or isolated), and three did not specify the cell source. Fluorescent tagging was rare: only six scaffolds used green fluorescent protein (GFP)‐expressing Human Umbilical Vein Endothelial Cells (HUVECs), and one opted for red fluorescent protein (RFP)‐expressing HUVECs, mostly for cell tracking purposes. Finally, most EC lines were derived from the umbilical vein (24 scaffolds), followed by dermal (n = 5), bloodstream (n = 3), aortic (n = 2), and pulmonary artery (n = 2). Other sources (n = 9) included brain capillary and external jugular vein tissue, while one scaffold did not specify the tissue of origin.

##### 3.4.1.2 Non‐endothelial cell (Non-EC) lines

Non‐endothelial lines were more diverse, encompassing 74 cell types and were used across 87 scaffolds (Fig. 4B), including human (56 scaffolds), murine (mouse, n = 9; rat, n = 9), bovine (n = 6), porcine (n = 4), simian (baboon, n = 1), and canine (n = 1) origins, with one study not specifying the species. Cell types included fibroblasts (n = 18), stem cells (n = 16), and stromal cells (n = 15), followed by keratinocytes (n = 5) and preadipocytes (n = 4). 29 other cell types were also used. Adipose tissue was the most frequent source (n = 21), subsequently, skin (n = 9) and bone marrow (n = 3). Other origins (n = 34) included skeletal muscle, alveolar tissue, cornea, and pancreas. The origin of 20 cell lines used was not specified.

Most non‐ECs were isolated from different tissues (59 scaffolds; including 2 transfected cell lines derived from isolated primary cells), with fewer being commercially sourced (n = 23; including 6 that were derived or differentiated from commercial sources. In five scaffolds, the cell source was not specified.

#### 3.4.2 *In vivo* models

**Fig. 5:**
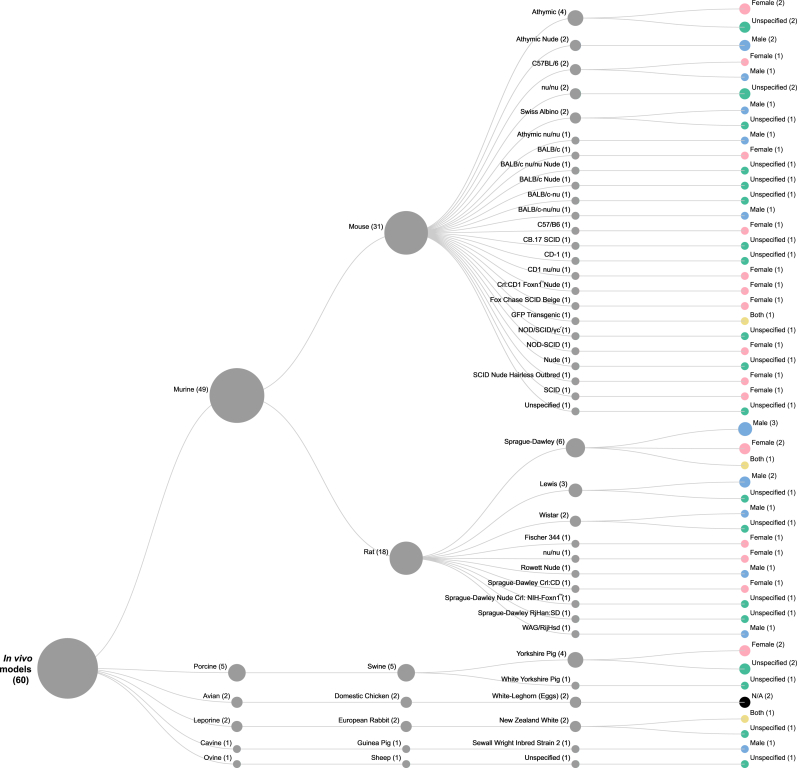
***In Vivo* Models Used**, by *taxa*, species, and sex of specimen, showing the number of times a model was used in the corpus.

Of the 54 studies employing *in vivo* models (Fig. 5), most (n = 50) utilised a single *in vivo* model. A few studies used multiple models: two studies used two models, one used three, and one used six different models. The most frequent *taxa* were murine models (49 in total), encompassing 31 mouse and 18 rat models. Mouse models included athymic (n = 7, including athymic nu/nu and athymic nude), Swiss Albino (n = 2), C57BL/6 (n = 2), and other 20 mouse strains. As for rat models, Sprague‐Dawley (n = 6), Lewis (n = 3), Wistar (n = 2), and a range of other strains (n = 7) were used. Other *taxa* included porcine models (n = 5, Yorkshire pigs), leporine models (n = 2, New Zealand White rabbits), an ovine model (n = 1, sheep, specific strain unspecified), and a cavine model (n = 1, guinea pig). Avian models (n = 2) consisted of White Leghorn chicken eggs.

The sex of the *in vivo* models used was reported on a per‐study basis: a single sex indicated exclusive use, ‘both’ meant no sex distinction was made, and ‘unspecified’ indicated that the sex was not reported. Sex preference varied by species; for example, among the mouse models (n = 31) used females more often (35.48 %) than males (19.35 %), with 3.23 % using both sexes and 41.94 % unspecified. In contrast, of the 18 rat models used, males were more common (44.44 %) than females (27.78 %), with 5.56 % using both sexes and 22.22 % unspecified.

### 3.5 Manufactured objects & features

**Fig. 6:**
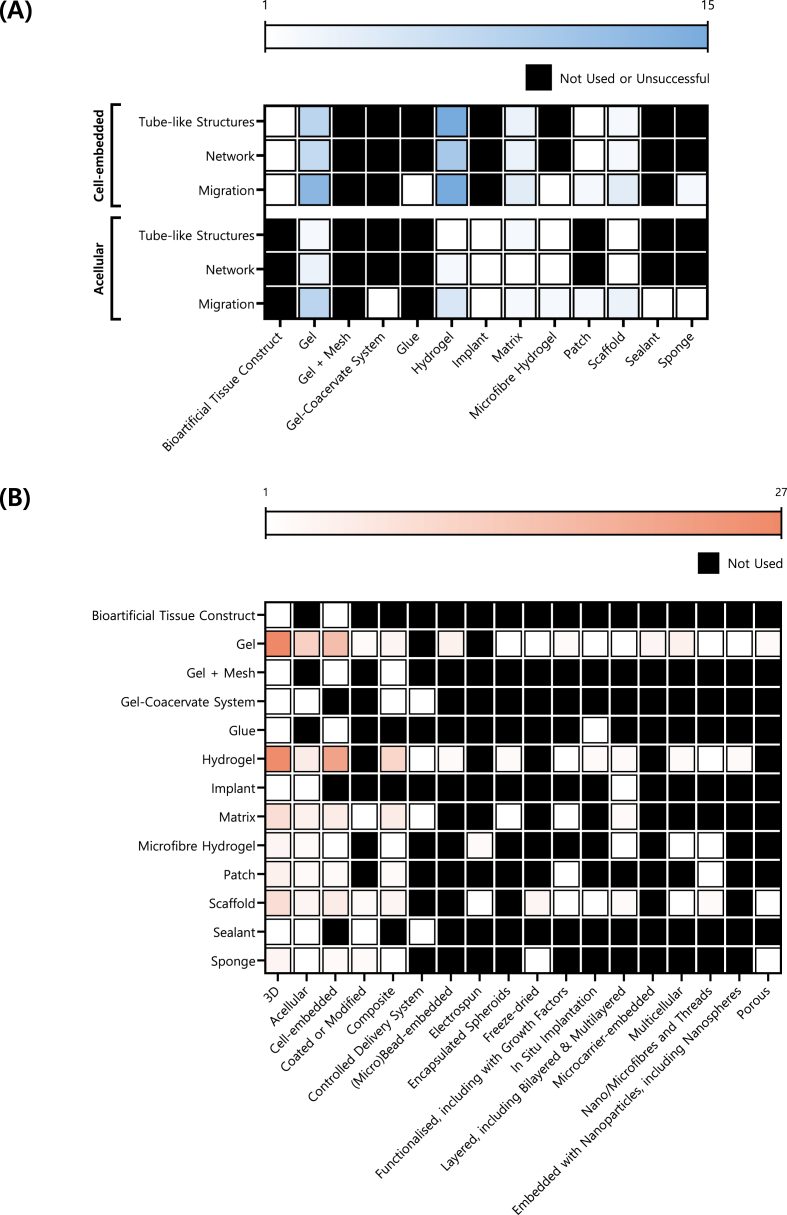
**Tissue Engineering Scaffolds Successfully Supporting Endothelial Formation or Migration Processes (EFMPs), Categorised by Type of Manufactured Object.** Further insights are provided into scaffold or construct design based on (A) the successful use of acellular versus cell-embedded models, and (B) the associated features of manufactured objects, as reported in the studies. Colours indicate the number of studies in which each manufactured object was reported as successful, with a darker colour indicating higher frequency of use. Only features mentioned in at least three studies are shown here; Figure S5 presents all features regardless of the number of studies citing them.

The selection of tissue engineering scaffolds demonstrates varying degrees of success in promoting vascularisation, depending on the scaffold type (*i.e.,* manufactured object type), whether cells were pre‐embedded, and the type of EFMPs evaluated. In the studies analysed, 13 different manufactured object types were identified (Fig. 6A). In most studies (95.06 % of studies), a single type of manufactured object was used, but four studies evaluated two different manufactured objects in each study.

Thirteen different types of manufactured objects were identified, based on the terminology used by the authors to describe their scaffolds. These terms are treated as separate categories to reflect reported differences in material composition, structural properties, and intended function (Fig. 6B). For example, matrices refer to fibrous or porous structures; gels are soft 3D network, semi‐solid materials formed from crosslinked polymers; and hydrogels are a subclass of gels which use water as a medium and are characterised by a high-water content. Composite constructs were also reported, including gel + mesh systems (gels integrated with a structural mesh) and microfibre hydrogels (hydrogels with embedded fibres).

#### 3.5.1 Acellular manufactured objects

Acellular scaffold performance varied across EFMPs (Fig. 6A). Gels were the most frequently tested manufactured objects (8–11 scaffolds) but with varying outcomes. Success rates were relatively low in tube (18.18 %) and network formation (33.33 %), but higher in migration (72.73 %). A similar pattern was observed with microfibre hydrogels (n = 2) and scaffolds (n = 3), with tube and network formation success rates being lower (50.00 %) than in endothelial migration (100.00 %).

However, this pattern was not replicated across all manufactured objects. Matrices showed more consistent success rates (66.67 % in both tube formation and migration; and 50.00 % in network formation); however, only 2–3 scaffolds were used. Hydrogels had greater variations in success rates: 50.00 % in tube formation (n = 2), 66.67 % in network formation (n = 3), and 100 % in migration (n = 5). Some manufactured objects were tested only once, limiting confidence in their success rates. For example, whenever tested, patches (n = 2), and implants, sealants, and sponges (one scaffold of each type) were all 100.00 % successful. On the other hand, gel‐coacervate systems failed in both tube and network formation but succeeded in migration. There were also cases where the manufactured object exhibited greater variability. In general, the acellular scaffold category is characterised by generally modest success rates and limited sample numbers, making it difficult to draw strong conclusions regarding the superiority of any specific scaffold type.

#### 3.5.2 Cell‐embedded manufactured objects

Cell‐embedded scaffolds, particularly hydrogels and gels, consistently showed higher success rates across the EFMPs (Fig. 6A). Hydrogels had a robust performance, with success rates of 92.86 % in tube formation (14 scaffolds), 88.89 % in network formation (n = 9), and 93.33 % in migration (n = 15). Gels also performed well, with 80.00 % success rates in tube formation (n = 10), 77.78 % in network formation (n = 9), and 92.86 % in migration (n = 14). Matrices were also commonly used (n = 5) and yielded moderate success: 60.00 % in tube and network formation and 80.00 % in migration. Patches (in tube and network formation) and hydrogel matrices (in endothelial migration) showed lower success (50.00 %), but were evaluated using only two scaffolds each, limiting interpretability. Other manufactured object types pre‐embedded with cells were tested only once and showed either complete success (*e.g.,* bioartificial tissue constructs [57], glues [131], microfibre hydrogels [76]) or failure (*e.g.,* gel‐mesh composites [82]).

Overall, cell pre‐embedded hydrogels and matrices consistently outperformed their acellular counterparts, especially where larger sample sizes were available. Notably, some scaffold types (*e.g.,* bioartificial tissue constructs) were exclusively tested in cell‐embedded configurations, precluding direct comparison with their acellular counterparts. These results highlight the benefits of pre‐seeding scaffolds with cells for vascularisation, although acellular scaffolds still have value in broader tissue engineering strategies.

#### 3.5.3 Manufactured object features

Thirteen distinct manufactured object types were identified, collectively described using 39 features (Fig. 6B & Figure S5). The most common feature was ‘3D’, mentioned in all 85 scaffolds. Features used to group scaffolds into two primary categories, cell‐embedded (63.53 %) and acellular (36.47 %), were frequently reported. ‘Composite’ was reported in 27 manufactured objects, while other frequent features included layered structures, including ‘bilayered’ and ‘multilayered’ designs (10.58 % of manufactured objects); ‘surface coating or modification’ (9.41 %); and ‘multicellularity’ (9.41 %). These terms suggest a move towards more biomimetic and structurally complex designs.

Less common were ‘functionalisation’, including the incorporation of growth factors; ‘(micro)bead embedded’; and the integration of ‘nano‐ or microfibres and threads’ (each, 7.06 %), indicating an increasing interest in multifunctionality and microarchitectural sophistication.

Advanced fabrication techniques, such as ‘3D printing’ (reported for 1 manufactured object) and ‘bioprinting’ (n = 2), along with properties like ‘magnetic responsiveness’ (n = 2), ‘slow degradation’ (n = 1), and ‘structural stabilisation’ (n = 1), were rare, suggesting that such features remain specialised approaches. Gels and hydrogels were not only very popular (used in 85 and 86 scaffolds, respectively), they were also associated with the greatest variety of features (n = 22 and n = 21, respectively), highlighting their versatility and prominence. Unique features associated with gels and hydrogels included the encapsulation of spheroids (3 manufactured objects); embedding of microcarriers (n = 3); multicellularity (n = 6); and nanoparticle embedding, including nanospheres (n = 3), emphasising their capacity for complex cellular and material interactions, as well as scaffold functionality.

### 3.6 Assays used to evaluate endothelial formation and migration processes (EFMPs)

**Fig. 7:**
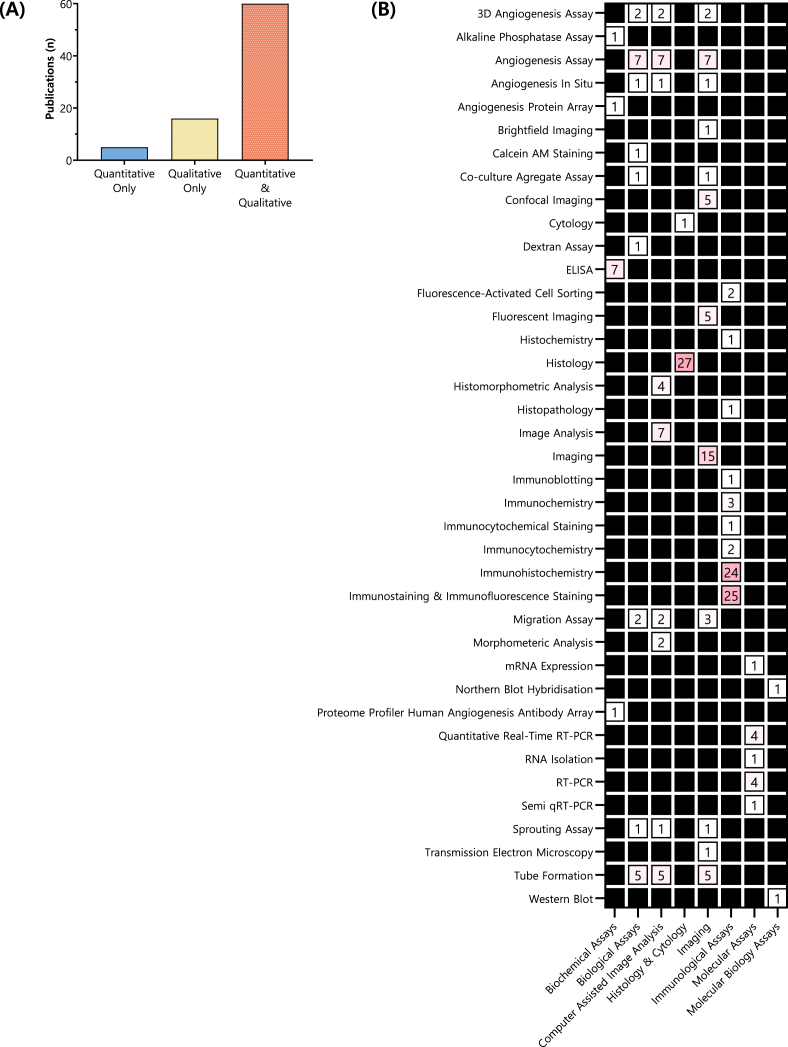
**Assays Used to Evaluate Endothelial Formation or Migration Processes (EFMPs)**. (A) Type of data used to evaluate EFMPs, shown by number of studies using quantitative, qualitative or combined data types. (B) Assays used to evaluate the success of EFMPs, grouped by number of studies in which they were employed. Assays were classified into eight general categories based on the evaluation method.

Across the 81 studies analysed, methods used to assess EFMPs varied by data type (Fig. 7A). Five studies relied solely on quantitative data (6.17 % of the corpus), 16 on qualitative data only (19.75 %), and 60 used both (74.07 %), highlighting the value of combining objective metrics with biological context.

A total of 39 distinct assays were identified and grouped into eight categories (Fig. 7B). Immunological assays were the most frequently employed, featured in 60 studies (74.07 %). Of these, the most frequently used were immunostaining and immunofluorescence staining (n = 25), followed closely by immunohistochemistry (n = 24). Other immunological techniques, such as immunochemistry, immunocytochemistry, and immunoblotting, were used less frequently, appearing in up to three studies each.

Imaging techniques featured in 47 studies, including fluorescent imaging (n = 5), confocal microscopy (n = 5), and functional assays such as angiogenesis assays (n = 7), tube formation assays (n = 5), and migration assays (n = 3), to visualise morphological changes. Computer‐assisted image analysis (n = 31) was often coupled with biological assays (n = 21) and imaging techniques, particularly in the context of standard (n = 7) and three‐dimensional (n = 2) angiogenesis assays, as well as migration assays (n = 2). This combination enabled the quantitative assessment of network parameters [66,68,75,84,91,111,121], including total tube or sprout length and the number of branch points.

Histology was used in 27 studies (Fig. 7B), whereas cytology appeared only once. Molecular (11 studies), biochemical (n = 10), and molecular biology (n = 2) assays were less frequently used across the dataset. Specific techniques within these categories included: Enzyme‐Linked Immunosorbent Assay (ELISA; n = 7), real‐time reverse transcription polymerase chain reaction (RT‐PCR; n = 4), quantitative RT‐PCR (n = 4), Western blot (n = 1), and mRNA expression analysis (n = 1).

### 3.7 Fibrin tissue engineering scaffolds for muscle research

To provide a focused overview of fibrin scaffold applications, muscle was selected as the focus of a sub-analysis because it is a widely studied tissue, with sufficient *in vitro* and *in vivo* studies to enable a meaningful synthesis without being overly broad. Its subtypes (cardiac, skeletal, and smooth) offer diverse applications, and its high metabolic demand makes vascularisation essential, an area where fibrin's pro-angiogenic properties are particularly advantageous for oxygen delivery and function. Moreover, muscle requires strong structural support, making the study of different scaffold designs, including the selection of manufactured objects, especially relevant. This subsection examines the subset of muscle-focused studies in terms of muscle and study types, *in vivo* models, cell lines, and manufactured objects employed.

#### 3.7.1 Muscle types & study distribution

**Fig. 8:**
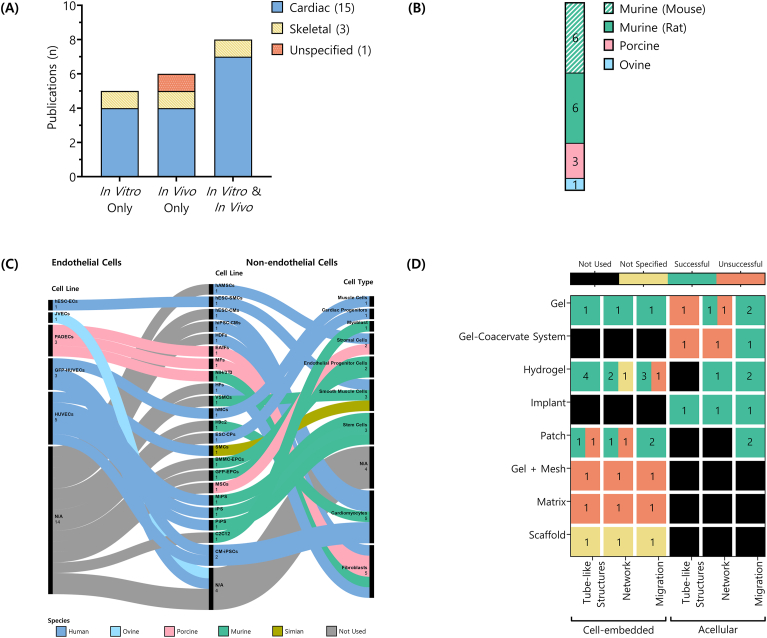
**Fibrin Tissue Engineering Scaffolds for Muscle Research.** (A) Number of studies categorised by study and muscle type. (B) *In vivo* models used. (C) Endothelial (EC) and non-endothelial cell (Non-EC) lines used, classified by species of origin and type. (D) Types of manufactured objects tested with or without pre-embedded cells and their reported association with the outcomes of endothelial formation or migration processes (EFMPs). For each manufactured object, the number of successful, unsuccessful, and unspecified outcomes is shown.

Of the 81 studies included in this SR, 19 focused on muscle tissue engineering: 15 on cardiac muscle, three on skeletal muscle, and one on muscle tissue in general (Fig. 8A). Interestingly, none targeted smooth muscle. Experimental approaches varied, comprising exclusively *in vivo* (6 studies) or *in vitro* (n = 5) models, and a combination of both (n = 8).

#### 3.7.2 *In vivo* models

14 studies incorporated *in vivo* work and used a range of models (Fig. 8B): mice and rats were equally prevalent (used in 6 studies each), followed by swine (n = 3) and sheep (n = 1). While most studies (85.71 %) used a single *in vivo* model, two studies used multiple models: (i) immunodeficient NOD/SCID/γc^−/−^ mice and Yorkshire swine; (ii) Sprague‐Dawley and nude rats. The most utilised strains were Sprague‐Dawley rats and Yorkshire swine (n = 3 each), and C57BL/6 mice (n = 2). Other strains, such as nude rats, Wistar rats, Lewis rats and BALB/c‐nu mice, appeared only once.

#### 3.7.3 Cell lines

Cell lines were heterogeneously used across the 17 studies that included *in vitro* research (Fig. 8C). Many of these studies evaluated different outcomes, with some focusing only on cell migration, others exclusively on tube formation, and a few evaluated both. Of these, nine studies used only non‐endothelial lines, two only endothelial lines, and six used both. Altogether, 25 distinct cell line combinations were reported. For instance, fibroblasts and porcine aortic ECs (PAOECs), cardiomyocytes with either GFP‐HUVECs or HUVECs; and various stem cell‐EC co‐cultures. Five studies used multiple cell line combinations within the same study, often pairing different endothelial or non‐endothelial types independently.

Eight studies used 5 different endothelial lines and, in some cases, in multiple experimental setups (*i.e*., an EC line used in combination with a non‐EC line) per study. These cell lines were predominantly of human origin (6 different studies, in 7 different experimental setups), alongside one porcine and one ovine line (1 study, 1 setup each).

The EC lines used were: HUVECs (3 studies, 3 setups), GFP‐HUVECs (3 studies, 3 setups), PAOECs (1 study, 3 setups), human embryonic stem cell‐derived ECs (1 study, 1 setup), and jugular vein ECs (1 study, 1 setup). Non‐EC lines were more diverse, appearing in 15 studies (22 setups), and included human (7 studies, 9 setups), mouse (3 studies, 5 setups), rat (3 studies, 4 setups), porcine (3 studies, 2 setups), and simian (1 study, 1 setup) sources. While most non‐EC lines were used only once (*e.g.,* MSCs, NIH/3T3, C2C12 myoblasts), cardiomyocytes and fibroblasts were the most frequently used (n = 5, each), followed by smooth muscle cells (n = 3), stem cells (n = 3), stromal cells (n = 2), and cardiomyocyte‐like cells derived from induced pluripotent stem cells (CM‐iPSCs; n = 2).

#### 3.7.4 Impact of manufactured objects on successful endothelial formation and migration processes (EFMPs)

Each of the 19 studies used a single manufactured object (Fig. 8D). Hydrogels were the most common (7 studies), followed by patches (n = 4), gels (n = 3), gel‐coacervate systems, implants, matrices, gel‐mesh composites, and scaffolds (n = 1, each). Scaffolds were described as such by the authors, without specifying further details. Of these manufactured objects, 57.89 % incorporated embedded cells, and the rest were acellular. Testing typically encompassed the three EFMPs (63.16 % of studies), but some evaluated only one (26.31 %) or two (10.53 %) outcomes.

Success rates (Equation (1)) varied based on manufactured object type and cell embedding (Fig. 8D). Pre‐embedded hydrogels and gels achieved 100 % success across all processes, except for gels in endothelial migration (75 %). In contrast, gel‐mesh composites and matrices had no successful outcomes. Patches yielded mixed results, with 100 % success in endothelial migration but only 50 % success in tube and network formation. However, the low number of replicates per scaffold type, often limited to a single study, warrants caution in interpretation. Additionally, three cases did not report the outcome, so this data was excluded from the success rate calculation.

Acellular scaffolds displayed a slightly different trend: all the manufactured objects evaluated (*e.g.,* gels, hydrogels, implants, patches) achieved a 100 % success rate except for gels in tube (0 %) and network formation (50.00 %). Gel‐coacervate systems failed to support tube and network formation (0 % in both cases).

### 3.8 Risk of bias & quality assessment

**Fig. 9:**
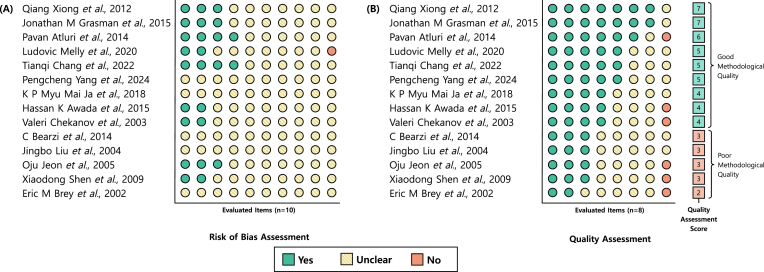
**Risk of Bias and Quality Assessment.** Here, the Risk of Bias and the Quality Assessment of *In Vivo* Muscle Models is shown. Articles were evaluated using 10 SYRCLE and 8 CAMARADES items. For each article, items are grouped by assessment outcome: ‘Yes’, followed by ‘Maybe’, and then ‘No’. Articles are ordered by overall quality assessment score, from highest to lowest. **(A)** Risk of bias (RoB) was evaluated using SYRCLE's RoB tool [42]. All ten items were assessed: (1) Was the allocation sequence generated and applied properly? (2) Were the groups similar at baseline or were they adjusted for confounding factors in the analysis? (3) Was the allocation adequately concealed? (4) Were the animals randomly housed during the experiment? (5) Were the carers and/or investigators blinded to the knowledge of which intervention each animal received during the experiment? (6) Were the animals randomly selected for outcome assessment? (7) Was the outcome researcher blinded? (8) Were incomplete outcome data adequately addressed? (9) Are the study reports free of selective reporting of results? (10) Was the study apparently free of other problems that could result in a high RoB? **(B)** Methodological quality was assessed using a modified CAMARADES checklist [49]. The eight items evaluated were: (1) Sample size calculation, (2) Random allocation to treatment or control, (3) Blinded assessment of outcome, (4) Use of an appropriate animal model, (5) Use of anaesthetic without significant intrinsic neuroprotective activity, (6) Compliance with animal welfare regulations, (7) Peer-reviewed publication, and (8) Statement of potential conflicts of interest.

The RoB and quality of the studies were assessed using SYRCLE's RoB and CAMARADES checklists, respectively. The detailed breakdown, per study, of the individual answers for each of the ten items of SYRCLE’S RoB and of the eight items in the CAMARADES checklist is available in the online repository (please refer to *Data Availability*). To provide a visual overview of these assessments, Fig. 9 presents a graphical summary of the risk of bias and quality assessment evaluations for the corpus subset of 14 muscle studies comprising *in vivo* work.

#### 3.8.1 Risk of bias

SYRCLE's RoB tool (Fig. 9A) revealed limited methodological transparency across the studies. For ≥60 % of the ten items, particularly those related to allocation concealment, random housing, blinding, and selective outcome reporting. In these cases, risk levels were marked as ‘unclear’ across all studies due to insufficient detail for assessment.

Items most often rated as ‘low risk’ were “other sources of bias” (57.14 % of studies), “baseline characteristics” (42.86 %), “random outcome assessment” (28.57 %), “sequence generation” (21.43 %), and “blinding” (21.43 %). Only one study received a ‘high risk’ rating for a single item.

#### 3.8.2 Quality assessment

Study quality was evaluated using a modified CAMARADES checklist (Fig. 9B). Methodological quality varied considerably: of the studies evaluated, 64.29 % were rated as having ‘good quality’ (score ≥4), while 35.71 % were deemed as ‘poor quality’ (score ≤3). The highest score achieved was 7 (14.29 % of studies) and the lowest was 2 (7.14 %), but no study achieved the maximum score of 10. The median score was 4, which was the minimum required to qualify as ‘good methodological’ quality.

While all studies “used an appropriate animal model” and were “peer‐reviewed”, 64.29 % of them reported data on the “use of anaesthetics” and “compliance with animal welfare regulations”. Only 28.57 % reported “random allocation to groups”, and 14.29 % included “sample size calculation” and “blinded outcome assessment”. These items were often marked as ‘unclear’ due to insufficient information. Notably, 42.86 % of studies failed to clearly declare potential conflicts of interest, while 7.14 % were ‘unclear’.

## 4 Discussion

This SR provides a comprehensive synthesis of current evidence on the use of fibrin‐based scaffolds for angiogenesis, including microvasculature formation, in soft tissues, with a particular focus on their capacity to support EFMPs. The findings confirm that fibrin, due to its intrinsic bioactivity and compatibility with (micro)vascular cells, is a promising scaffold material; however, success highly depends on the scaffold design, particularly the specific formulation and manufactured object type. Scaffold composition played a critical role in determining outcomes, a finding aligned with prior evidence emphasising the importance of the cellular context and scaffold design parameters in enabling EFMPs [135].

A key trend that emerged was that tube and network formation outcomes were generally consistent with one another, but these did not necessarily align with those observed for endothelial migration. This difference is likely due to the environment in which the scaffold is immersed: successful migration can be achieved with a broader range of scaffold formulations, suggesting that parameters critical for tube and network formation may not be as restrictive for migration processes. Reflecting these trends, we could identify the most promising strategies for successful EFMPs.

For successful tube and network formation, the most effective strategies involve using commercial fibrinogen, particularly of human origin, and avoiding the addition of other materials. Not using a crosslinker or using *CaCl*_*2*_ generally yielded higher success rates; however, these comparisons are limited by small and unequal group sizes and therefore require cautious interpretation. Crosslinking, typically with *CaCl*_*2*_ or other polymers, is commonly employed to enhance the mechanical strength and reduce the biodegradation rate of fibrin scaffolds [27]. In addition, the inclusion of thrombin as a coagulant and pre‐embedding cells also contributed positively.

In contrast, preferred strategies for endothelial migration were less distinct. Both human and bovine fibrinogen yielded similarly high success rates, and the inclusion of additional materials had little impact. Outcomes were comparable regardless of whether *CaCl*_*2*_ was used as a crosslinker or omitted altogether. Avoiding a crosslinker appeared to improve migration, though this finding is based on limited data and should be interpreted with caution. Pre‐embedding cells had little influence on migration outcomes, most probably because of the fibrin serving as a cue directing cell motility [134]. Moreover, like many other cell types, ECs can migrate in different ways, individually, in chains, or as sheets, enabling them to effectively navigate through and populate the scaffolds [136]. This may also suggest that pre‐embedding is less critical for migration and that cell motility within the fibrin matrix might be more dependent on other factors (*e.g.,* matrix porosity, elasticity or degradability), which may influence cell behaviour and vascularisation [39].

Focusing on the key elements of the scaffold formulation explored here, the origin of fibrin had no noticeable impact on endothelial tube and network formation success, while commercially sourced fibrin outperformed extracted fibrin in supporting endothelial migration. This outcome may be partly due to inconsistent reporting and differences in extraction methods, in addition to many other components incorporated into the scaffolds. However, the use of commercial fibrin introduces considerations regarding batch-to-batch variability, which remains a significant challenge in fibrin scaffold production and continues to hinder direct comparison between studies [38].

Among the scaffolds for which fibrinogen source was reported, human and bovine fibrinogen were the most commonly used, with human fibrinogen being more frequently employed, and were also associated with the highest success rates. Human‐derived fibrinogen consistently promoted enhanced tube and network formation; however, when assessing endothelial migration, the difference between human‐ and bovine‐derived fibrinogen was not as significant. Beyond some structural differences between human- and bovine-derived fibrinogen, one key distinction is the location of the RGD (Arg-Gly-Asp) sequence, crucial for integrin binding and cell adhesion. Although human fibrinogen contains the RGG (Arg-Gly-Gly) analogous sequence, it still appears able to mediate cell interactions [137,138]. While bovine fibrinogen promotes cell migration, it may present some limitations, like increased immunogenicity [139] and faster degradation rates [140] compared to its human counterpart.

Another factor directly impacting the scaffold properties are the concentrations of fibrinogen, thrombin, and the selected crosslinker used, which, in turn, are associated with variable effects on EFMPs. Although it was not possible to draw conclusions based on specific concentrations used in the studies included in this SR due to inconsistent reporting across papers, it is well established in literature that variations in fibrinogen and thrombin concentration impact the final fibre thickness, resulting in different mechanical properties, pore sizes, and degradation rates which significantly influence cell migration, differentiation, and angiogenesis [141].

Fibrinogen and thrombin sources, their addition, and their concentrations directly affect the mechanical properties of the scaffolds [142]. The mechanical strength of scaffolds, such as stiffness, influences cellular processes, as mechanical signals are converted into biochemical signals through cell receptors and integrins [21]. In particular, the mechanical properties of scaffolds influence EC expression of matrix metalloproteinases and growth factors, as well as their differentiation, sprouting, and migration [135]. Migration, especially, is highly sensitive to external stimuli and requires degradation of existing ECM and interactions with surrounding cells [143]. The migration process favours a soft matrix, so the negative impact of thrombin addition on migration might be due to an increase in mechanical strength [141,143,144].

The literature indicates that higher fibrinogen concentrations result in thicker fibrin fibres, and smaller pores and interconnectivities that can limit cell migration [145]. Conversely, high fibrinogen concentrations may be favourable for cell attachment and other cellular processes such as network formation, due to the increased availability of cell‐binding sites with the fibrin matrix [29]. Fibrin interacts with cells through several receptors, particularly with ECs through integrin *α*_*v*_*β*_*3*_, integrin *α*_*5*_*β*_*1*_ [32] and vascular endothelial cadherin [137] which eventually affect cell morphology [146]. This may explain why fibrinogen content has differentially affected tube and network formation compared to cell migration.

On the other hand, the literature shows that increasing thrombin concentration reduces the fibre thickness, resulting in thinner, more tightly packed fibrils [147], which can affect the final pore structure of the scaffold. In this SR, thrombin was the only material chosen as a coagulant and, when used, it positively affected EFMPs. However, thrombin addition induces angiogenic response human ECs through the expression of VEGF [148], as well as cell surface receptors such as protease‐activated receptors (PAR1 and PAR4) [18]. This may further explain why thrombin addition enhanced tube and network formation. Based on the data, the selection of crosslinking agent is more complex: although calcium‐based crosslinkers such as *CaCl*_*2*_ were commonly used to provide structural stability to the scaffold, their contribution to angiogenesis was not always favourable. This may be due to the extracellular concentrations of calcium and chloride interfering with natural cellular processes and osmotic pressure, respectively [144].

As for the inclusion of other materials besides fibrin, coagulants, and crosslinkers, the data reveals that composite scaffolds combining fibrin with polymers or growth factors offered mechanical or biochemical advantages, likely the reason for which these materials were incorporated in the formulation. Nevertheless, these composite scaffolds were not universally superior in supporting EFMPs. Composite scaffolds were commonly used, likely due to the need to improve mechanical properties of fibrin [38]. This suggests that increased complexity of materials does not necessarily translate into improved biological performance. However, composite scaffolds may reflect efforts to tailor scaffold properties to specific soft tissue applications, highlighting the versatility of fibrin as a base material in tissue engineering. For example, the inclusion of collagen in skin scaffolds could help mimic the natural tissue more closely [149] and improve cell adhesion [149]. Similarly, incorporating synthetic polymers like PEG can help modify scaffold degradation rates and other mechanical properties [150,151], suggesting that slower scaffold degradation may promote cell infiltration and enhance pro-angiogenic effects [27].

Pre‐embedding cells also appeared to be beneficial for scaffold performance, particularly in promoting tube and network formation, although it had a lesser impact on EC migration. This could be attributed to pre‐embedded scaffolds regulating vascular formation *in vivo* through cell‐cell and cell‐matrix interactions, as well as interactions with biochemical cues [152]. Biochemical molecules like growth factors are essential regulators of angiogenesis, interacting through specific receptors. This likely explains why several of them were commonly added to cell culture media [153], as reported in this SR. However, their concentrations must be carefully optimised, as excessively high levels of VEGF can lead to deformity in vascular formations [152]. Overall, endothelial formation or migration processes require a delicate balance between degradation, mechanical strength and biochemical signalling [40].

Beyond the scaffold formulation, other factors such as the cell lines used, played a role in the outcomes of endothelial formation and migration processes. Among the non‐EC types used in angiogenic models, one of the most common cell sources used in this SR was adipose tissue. This is likely due to adipose derived cells demonstrated angiogenic potential *via* paracrine secretion of growth factors such as VEGF, as well as their ability to differentiate into ECs [154]. Fibroblasts were the most used cells in this SR, likely because most of the included studies focused on connective tissues, where fibroblasts are the main cell type [155]. Moreover, co‐ culture of fibroblasts with ECs has been shown to promote angiogenesis by supporting EC migration and proliferation [17] by secreting angiogenic factors [156]. Within ECs, the most common source was umbilical cord‐derived cells. This aligns with the existing literature, which identifies HUVECs as the most frequently used, due to their easy isolation, low immunogenicity and suitability as an *in vitro* endothelial model [17,157]. More articles reported the use of commercially sourced ECs, which is beneficial as it reduces donor‐ related variability that could impact experimental outcomes [17].

As demonstrated by the wide range of assays used across the included studies, no single assay emerges as universally ideal for evaluating the various mechanisms involved in angiogenesis, as each offer distinct benefits and limitations. The variety of assays and *in vivo* models used in this SR reflects the complexity of EFMPs [158,159]. Although several scaffold types were reported, it is not surprising that 3D systems were the most frequently mentioned, as cell‐cell and cell‐matrix interactions are closer to the natural physiological environment, regulating cell proliferation, differentiation, and signalling [17]. It has also been reported that 3D *in vitro* assays better mimic angiogenesis than 2D assays, with the expression of angiogenic genes being more pronounced in former [160].

In the studies included in this SR, we identified a wide range of scaffold formulations, with some articles testing multiple combinations per publication, in some cases used as a control. Similarly, the included studies employed a range of experimental approaches, with nearly half of them using more than one study type, primarily a combination of *in vitro* and *in vivo* methods. This methodological diversity highlights both the comprehensive scope of the research and the translational potential of fibrin scaffolds for soft tissue engineering. However, it also presents challenges for direct comparison, reflecting the field's exploratory efforts to optimise fibrin scaffolds for endothelial formation and migration.

The subset of articles focusing on muscle tissue angiogenesis, including microvasculature formation, revealed that there is a predominance of cardiac over skeletal muscle, and none addressing smooth muscle. Interestingly, these two tissue types were addressed with a wide variety of manufactured scaffold types, suggesting a lack of consensus on optimal design for muscle applications. This is a relevant finding, as existing literature suggests that scaffold type, stiffness, and fibrinogen content influence myogenic cell differentiation [161]. The diversity of *in vivo* models and cell lines used, particularly the variety of non‐ECs, highlights the complexity of replicating the muscle microenvironment and the multifactorial nature of EFMPs. While angiogenesis remains a significant clinical challenge in muscle tissue engineering, the literature suggests that pre‐embedding cells within constructs is a promising strategy to facilitate the formation and interconnectedness of endothelial structures [162]. In this SR, the findings confirmed this, particularly in hydrogel and gel‐based scaffolds, which demonstrated the highest success rates across EFMPs. However, the heterogeneity of study designs coupled with the limited number of replicates for each scaffold type limits the generalisation of these findings. Nonetheless, the trends identified reinforce the importance of scaffold‐cell interactions and support the use of precellularised constructs to enhance microvascularisation in muscle tissue engineering.

This SR revealed several limitations in both the included literature and the review process itself. In terms of the corpus, it is important to note that some studies were reported by the same research groups and overlapping researchers (*e.g.,* Nehls [127,129,130], Verseijden [98,103,107], Barreto-Ortiz [76,90], and Borges [111,115] groups). This may have led to over‐representation of certain experimental procedures and research priorities.

A major limitation of the included studies was the inconsistencies and lack of reporting parameters such as the source and concentration of fibrinogen, thrombin, *CaCl*_*2*_, coagulant ratios, scaffold fabrication methods, and cell seeding protocols. Such inconsistencies in reporting critical methodological parameters significantly limit reproducibility; making it harder for independent research groups to reliably replicate findings, validate results, or build on existing work. To address this issue, we developed a “Minimum Information for Fibrin Scaffold Experiments (MIFSE) Checklist” (available in the Supplementary Materials, as Appendix II) to standardise reporting in fibrin scaffold studies. This tool ensures inclusion of critical methodological details and other key parameters, thereby supporting reproducibility and cross-study comparison.

This lack of reporting also affected the SR itself, as it limited the strength and range of conclusions that could be drawn. It restricted the conclusions about direct relationships between critical scaffold parameters and final scaffold properties or cell responses, which could have provided valuable insights for scaffold design. These methodological inconsistencies, coupled with considerable heterogeneity in study designs (Table S3), scaffold formulations (Table S4), and outcome measures, made it impractical to conduct a meta‐analysis, hence making the narrative synthesis the most viable option. Although aggregated success rates were used in this SR, it should be noted that this approach may oversimplify complex parameters and could introduce interpretive bias. While the data suggests trends, it is important to acknowledge the definition of “successful outcomes” used here may vary compared to other studies. An important limitation of this review is that studies involving different soft tissue types were analysed together, despite variations in their architectures and functions. These differences could influence the effects of fibrin concentration, embedding the scaffolds with ECs, or scaffold formulation on angiogenesis, so pooling these studies may therefore obscure tissue-specific outcomes.

A critical limitation of this review is the exclusion of non‐English publications and grey literature, which may have introduced language and publication bias. This could have reduced the comprehensiveness of the review, potentially excluding relevant studies with valuable insights. Furthermore, the database search was deliberately narrow, as we focused exclusively on fibrin-based studies and possibly this could restrict the broader contextual understanding of scaffold-based studies in general. Finally, while efforts were made to minimise bias through multiple independent reviewers and validated tools (*e.g.,* SYRCLE, CAMARADES), the presence of subjective interpretation in assessing outcomes, especially when relying on qualitative measures, could not be fully eliminated.

Future research should prioritise the development of factorial experimental designs to systematically evaluate the interactive effects of scaffold materials, crosslinkers, cell types, and environmental conditions. Standardising scaffold characterisation and reporting practices is essential for improving reproducibility and allowing meaningful comparisons across studies. This includes uniform reporting of fibrinogen source and concentration, cell incorporation techniques, and angiogenesis evaluation methods and metrics. Moreover, refining cell pre‐embedding protocols, such as specifying cell type, density, and culture conditions, will be critical to ensure consistent results and advance clinical translation (Figure S6). Such work will not only support the development of more functional tissue constructs but also inform best practices for scaffold design in *in vitro* and *in vivo* applications.

## 5 Conclusion

This SR highlights that the success of fibrin scaffolds in promoting angiogenesis, including microvascular EFMPs, in soft tissues highly depends on scaffold formulation, fibrinogen origin, cell pre‐embedding, and the selection of coagulant and crosslinker. Human‐derived, commercially sourced fibrinogen combined with thrombin and cell pre‐embedding were associated with the most consistent success in supporting tube and network formation, while effects on endothelial migration varied. These differences reflect the complexity of the endothelial processes, which are highly sensitive to scaffold formulation and design and, in turn, mechanical, biochemical, and structural properties. This SR also highlights the importance of selecting cell lines, *in vivo* models, and assays to evaluate EFMPs. A common finding amongst the included studies was inconsistent reporting of scaffold parameters, manufacturing methods, and characterisation techniques, which limits the ability to draw reliable conclusions. Finally, the insights derived from this SR could guide systematic approaches for developing fibrin scaffolds for *in vitro* microvascular models for regenerative medicine applications, disease modelling and preclinical testing by providing reproducible tissue‐engineered platforms.

## Registration & protocol

The protocol for this SR was registered with PROSPERO [registration number: CRD42025612994] [163] and with OSF [Registration DOI: 10.17605/osf.io/NVFDJ] [164]. It was also documented as a project in OSF [165].

## CRediT authorship contribution statement

**Carla Verónica Fuenteslópez:** Writing – review & editing, Writing – original draft, Visualization, Validation, Supervision, Project administration, Methodology, Investigation, Funding acquisition, Formal analysis, Data curation, Conceptualization. **Simge Bahcevanci:** Writing – review & editing, Writing – original draft, Validation, Project administration, Methodology, Investigation, Funding acquisition, Formal analysis, Data curation, Conceptualization. **Viorica Patrulea:** Writing – review & editing, Investigation, Funding acquisition, Data curation, Conceptualization. **Hua Ye:** Writing – review & editing, Supervision, Funding acquisition.

## Ethics approval & consent to participate

This article is a review article, specifically a systematic review. This research does not report any data associated with patients. This study does not report on or involve any animals, humans, human data, human tissue, or plants.

## Declaration of competing interests

HY is an associate editor for Bioactive Materials and was not involved in the editorial review or the decision to publish this article. All authors declare that there are no competing interests.

## Data Availability

Datasets related to this study can be found at https://github.com/carla-fuenteslopez/soft-tissue-fibrin-sr, hosted at the GitHub repository ‘Soft‐Tissue‐Fibrin‐SR’ and archived at https://zenodo.org/records/16921023 [166]. All data associated with this study is presented in this manuscript, in the Supplementary Materials (https://doi.org/10.1016/j.bioactmat.2025.10.019), and Appendix I. Appendix II: the “Minimum Information for Fibrin Scaffold Experiments (MIFSE) Checklist” is available at https://zenodo.org/records/16921390 [167].”

## Abbreviations

**3D:** Three‐dimensional; **bFGF:** Basic Fibroblast Growth Factor; **CAMARADES:** Collaborative Approach to Meta‐Analysis and Review of Animal Data from Experimental Studies; **EC(s):** Endothelial Cell(s); **ECM:** Extracellular Matrix; **EFMPs:** Endothelial Formation and Migration Processes; **GF/P/AA/E:** Growth Factors, Peptides, Amino Acids, and Enzymes; **GFP:** Green Fluorescent Protein; **HUVECs:** Human Umbilical Vein Endothelial Cells; **Non-EC(s):** Non-Endothelial Cell(s); **OSF:** Open Science Framework; **PAOECs:** Porcine Aortic Endothelial Cells; **PEG:** Polyethylene Glycol; **PROSPERO:** International Prospective Register of Systematic Reviews; **PRISMA:** Preferred Reporting Items for Systematic Reviews and Meta‐Analyses; **RFP:** Red Fluorescent Protein; **RGD**: Arg-Gly-Asp; **RGG**: Arg-Gly-Gly **RoB:** Risk of Bias; **RT‐PCR:** Reverse Transcription Polymerase Chain Reaction; **SR:** Systematic Review; **SYRCLE:** Systematic Review Centre for Laboratory Animal Experimentation; **VEGF:** Vascular Endothelial Growth Factor.

## Glossary

**Angiogenesis:** the formation of new blood vessels from pre-existing ones, involving endothelial cell invasion, migration, tube, and capillary network formation; **Tube formation:** the development of tube-like structures, capillary-like, tubes, capillaries, etc.; **Network formation:** the creation of interconnected tubes, tube-like structures, or capillaries contributing to the formation of a vascular network; **Endothelial cell migration:** the process by which endothelial cells interact with the matrix and orderly move into the extracellular matrix to form an endothelium; **Endothelial Formation or Migration Processes**: a collective term used in this manuscript to refer to tube formation, network formation, and endothelial cell migration; **Fibrin scaffolds**: term used in this manuscript to refer to fibrin-based and fibrinogen-based scaffolds.

## Footnotes

2 https://osf.io/.

3 https://www.crd.york.ac.uk/prospero/.

4 https://pubmed.ncbi.nlm.nih.gov/.

5 https://www.ovid.com/.

6 https://www.scopus.com/.

7 **SYRCLE’s RoB tool's ten Items:** (1) Was the allocation sequence generated and applied properly? (2) Were the groups similar at baseline or were they adjusted for confounding factors in the analysis? (3) Was the allocation adequately concealed? (4) Were the animals randomly housed during the experiment? (5) Were the carers and/or investigators blinded to the knowledge of which intervention each animal received during the experiment? (6) Were the animals randomly selected for outcome assessment? (7) Was the outcome researcher blinded? (8) Were incomplete outcome data adequately addressed? (9) Are the study reports free of selective reporting of results? (10) Was the study apparently free of other problems that could result in a high RoB?.

8 **Modified CAMARADES checklist's eight items:** (1) Sample size calculation, (2) Random allocation to treatment or control, (3) Blinded assessment of outcome, (4) Use of an appropriate animal model, (5) Use of anaesthetic without significant intrinsic neuroprotective activity, (6) Compliance with animal welfare regulations, (7) Peer‐reviewed publication, and (8) Statement of potential conflicts of interest.
