# Supplementary material for: Fibrin scaffolds for angiogenesis in soft tissue models: a systematic review
Source: Bioact Mater. 2025 Dec 4;56:703–25. doi: 10.1016/j.bioactmat.2025.10.019 (PMC12747203; doi:10.1016/j.bioactmat.2025.10.019)

APPENDIX I

Detailed Search Queries Used for each Database, displaying the number of records found on 28 October 2024.

(A) PubMed

**Database Name**	PubMed<br>PubMed includes MEDLINE, PubMed Central (PMC) and the NCBI Bookshelf.
**Limits and Restrictions**	Search terms included Medical Subject Headings (MeSH) terms.
**Search Filters Used**	No filters were applied in the initial search.
**Search Query**	(vascular network OR vascular network formation[MeSH Terms] OR microvascular network[MeSH Terms] OR microvascular network formation[MeSH Terms] OR neovascularization[MeSH Terms] OR angiogenesis OR vasculogenesis OR endothelial network OR endothelial network formation[MeSH Terms] OR microvessel[MeSH Terms] OR microcirculation[MeSH Terms] OR endothelial tube OR tube formation[MeSH Terms] OR tube-like)<br>AND<br>(soft tissue[MeSH Terms] OR muscle[MeSH Terms] OR nerve OR ligament[MeSH Terms] OR tendon[MeSH Terms] OR fascia[MeSH Terms] OR skin[MeSH Terms] OR fibrous tissue[MeSH Terms] OR connective tissue[MeSH Terms] OR synovial membrane[MeSH Terms])<br>AND<br>(scaffold OR construct[MeSH Terms] OR mesh[MeSH Terms] OR matrix OR matrices OR gel OR hydrogel[MeSH Terms] OR tissue engineered product)<br>AND<br>(fibrin[MeSH Terms] OR fibrinogen[MeSH Terms])

NIH National Library of Medicine
National Center for Biotechnology Information

Log in

PubMed®

(soft tissue[MeSH Terms] OR muscle[MeSH Terms] OR nerve OR ligament[Me

Search

Advanced Create alert Create RSS User Guide

Save Email Send to Sort by: Best match Display options

MY NCBI FILTERS

146 results

RESULTS BY YEAR

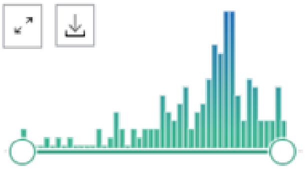

TEXT AVAILABILITY

- Abstract
- Free full text
- Full text

ARTICLE ATTRIBUTE

- Associated data

ARTICLE TYPE

- Books and Documents
- Clinical Trial
- Meta-Analysis
- Randomized Controlled Trial
- Review
- Systematic Review

PUBLICATION DATE

- 1 year
- 5 years
- 10 years

1 Basic components of connective **tissues** and extracellular **matrix**: elastin, fibrillin, fibulins, fibrinogen, fibronectin, laminin, tenascins and thrombospondins.
Halper J, Kjaer M.
Adv Exp Med Biol. 2014;802:31-47. doi: 10.1007/978-94-007-7893-1_3.
PMID: 24443019 Review.
Collagens are the most abundant components of the extracellular **matrix** and many types of **soft tissues.** Elastin is another major component of certain **soft tissues**, such as arterial walls and ligaments. Many other molecules, though lower in quanti ...
Cite Share

2 **Vascular Network** Formation on Macroporous Polydioxanone **Scaffolds.**
Heene S, Thoms S, Kalies S, Wegner N, Peppermüller P, Born N, Walther F, Scheper T, Blume CA.
Tissue Eng Part A. 2021 Oct;27(19-20):1239-1249. doi: 10.1089/ten.TEA.2020.0232. Epub 2021 Feb 24.
PMID: 33397206
In this study, **microvascular network** structures for **tissue engineering** were generated on newly developed macroporous polydioxanone (PDO) **scaffolds.** ...Fibrin-coated **scaffolds** were seeded with recombinant human umbilical vein **endothelia** ...
Cite Share

3 Chitosan-collagen-fibrinogen uncrosslinked **scaffolds** possessing skin regeneration and **vascularization** potential.
Dasgupta S, Gope A, Mukhopadhyay A, Kumar P, Chatterjee J, Barui A.
J Biomed Mater Res A. 2023 May;111(5):725-739. doi: 10.1002/jbm.a.37488. Epub 2022 Dec 27.
PMID: 36573698
Thus constructs, which promotes wound healing and supports **vascularization** has gained priority in **tissue engineering**. In this study, chitosan-collagen-fibrinogen (CCF) **scaffold** was fabricated using freeze-drying method without using any chemical crossl ...
Cite Share

4 Platelets and wound healing.
Nurden AT, Nurden P, Sanchez M, Andia I, Anitua E.
Front Biosci. 2008 May 1;13:3532-48. doi: 10.2741/2947.
PMID: 18508453 Review.
Platelets help prevent **blood** loss at sites of **vascular** injury. To do this, they adhere, aggregate and form a procoagulant surface favoring thrombin generation and fibrin formation. In addition, platelets express and release substances that promote **tissue** repa ...
Cite Share

PubMed Advanced Search Builder

PubMed®

User Guide

Add terms to the query box

All Fields | Enter a search term | ADD

Show Index

Query box

Enter / edit your search query here

Search

History and Search Details

Download | Delete

Search	Actions	Details	Query	Results	Time
#5	···	>	Search: (((soft tissue[MeSH Terms] OR muscle[MeSH Terms] OR nerve OR ligament[MeSH Terms] OR tendon[MeSH Terms] OR fascia[MeSH Terms] OR skin[MeSH Terms] OR fibrous tissue[MeSH Terms] OR connective tissue[MeSH Terms] OR synovial membrane[MeSH Terms]) AND (fibrin[MeSH Terms] OR fibrinogen[MeSH Terms])) AND (scaffold OR construct[MeSH Terms] OR mesh[MeSH Terms] OR matrix OR matrices OR gel OR hydrogel[MeSH Terms] OR tissue engineered product)) AND (vascular network OR vascular network formation[MeSH Terms] OR microvascular network[MeSH Terms] OR microvascular network formation[MeSH Terms] OR neovascularization[MeSH Terms] OR angiogenesis OR vasculogenesis OR endothelial network OR endothelial network formation[MeSH Terms] OR microvessel[MeSH Terms] OR microcirculation[MeSH Terms] OR endothelial tube OR tube formation[MeSH Terms] OR tube-like)	146	09:16:31
#4	···	>	Search: vascular network OR vascular network formation[MeSH Terms] OR microvascular network[MeSH Terms] OR microvascular network formation[MeSH Terms] OR neovascularization[MeSH Terms] OR angiogenesis OR vasculogenesis OR endothelial network OR endothelial network formation[MeSH Terms] OR microvessel[MeSH Terms] OR microcirculation[MeSH Terms] OR endothelial tube OR tube formation[MeSH Terms] OR tube-like	311,145	09:15:30
#3	···	>	Search: scaffold OR construct[MeSH Terms] OR mesh[MeSH Terms] OR matrix OR matrices OR gel OR hydrogel[MeSH Terms] OR tissue engineered product	1,260,602	09:15:05
#2	···	>	Search: fibrin[MeSH Terms] OR fibrinogen[MeSH Terms]	60,910	09:14:43
#1	···	>	Search: soft tissue[MeSH Terms] OR muscle[MeSH Terms] OR nerve OR ligament[MeSH Terms] OR tendon[MeSH Terms] OR fascia[MeSH Terms] OR skin[MeSH Terms] OR fibrous tissue[MeSH Terms] OR connective tissue[MeSH Terms] OR synovial membrane[MeSH Terms]	2,113,429	09:14:13

Showing 1 to 5 of 5 entries

(B) SCOPUS

**Database Name**	Scopus
**Limits and Restrictions**	No limits or restrictions were imposed.
**Search Filters Used**	The following three document types were excluded: Review, Book Chapter, and Book.
**Search Query**	vascular AND network OR vascular AND network AND formation OR microvascular AND network OR microvascular AND network AND formation OR neovascularization OR angiogenesis OR vasculogenesis OR endothelial AND network OR endothelial AND network AND formation OR microvessel OR microcirculation OR endothelial AND tube OR tube AND formation OR tube-like<br><br>AND<br><br>soft AND tissue OR muscle OR nerve OR ligament OR tendon OR fascia OR skin OR fibrous AND tissue OR connective AND tissue OR synovial AND membrane<br><br>AND<br><br>scaffold OR construct OR mesh OR matrix OR matrices OR gel OR hydrogel OR tissue AND engineered AND product<br><br>AND<br><br>fibrin OR fibrinogen

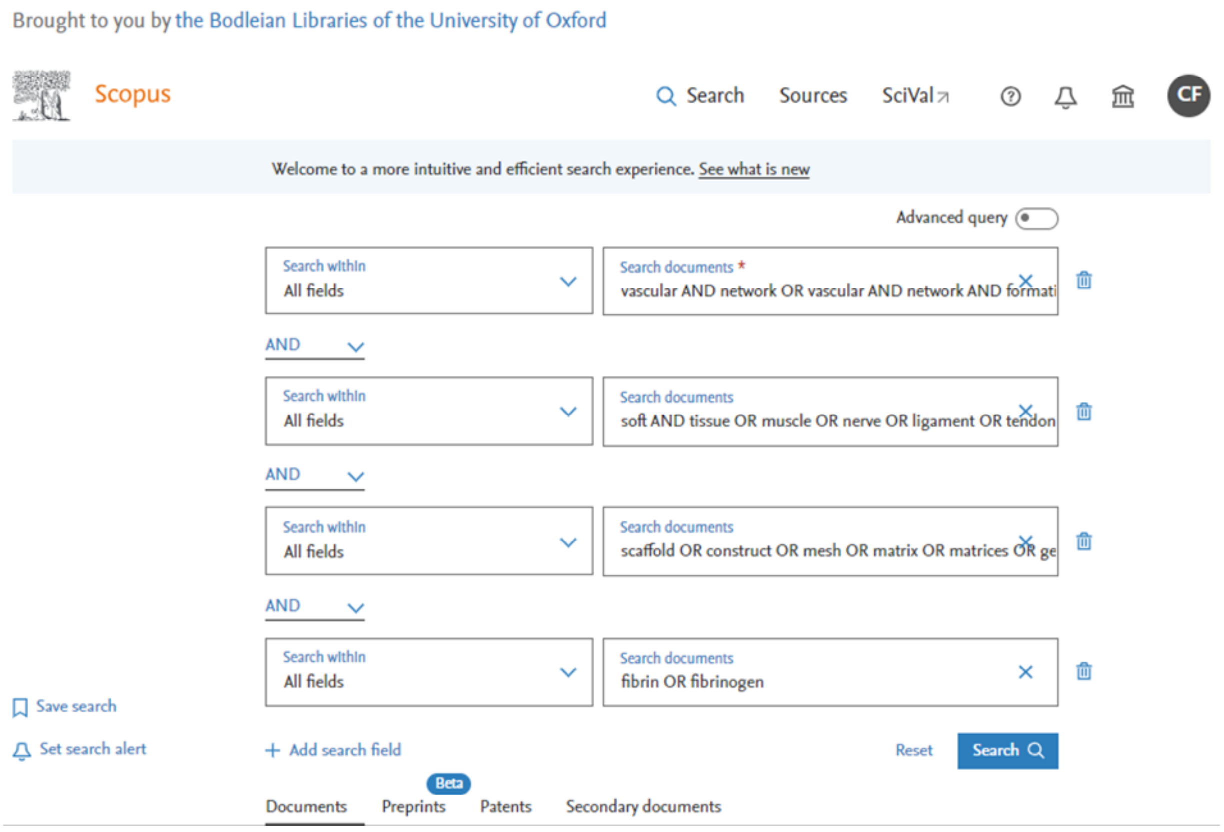

(C) OVID

**Database Name**	OVID
**Limits and Restrictions**	No limits or restrictions were imposed.
**Search Filters Used**	No filters were applied in the initial search.
**Search Query**	vascular network.mp. or vascular network formation/ or microvascular network/ or microvascular network formation/ or neovascularization/ or angiogenesis.mp. or vasculogenesis.mp. or endothelial network.mp. or endothelial network formation/ or microvessel/ or microcirculation/ or endothelial tube.mp. or tube formation/ or tube-like.mp.<br>AND<br>soft tissue/ or muscle/ or nerve.mp. or ligament/ or tendon/ or fascia/ or skin/ or fibrous tissue/ or connective tissue/ or synovial membrane/<br>AND<br>scaffold.mp. or construct/ or mesh/ or matrix.mp. or matrices.mp. or gel.mp. or hydrogel/ or tissue engineered product.mp.<br>AND<br>fibrin/ or fibrinogen/<br><br>[mp=title, book title, abstract, original title, name of substance word, subject heading word, floating sub-heading word, keyword heading word, organism supplementary concept word, protocol supplementary concept word, rare disease supplementary concept word, unique identifier, synonyms, population supplementary concept word, anatomy supplementary concept word]

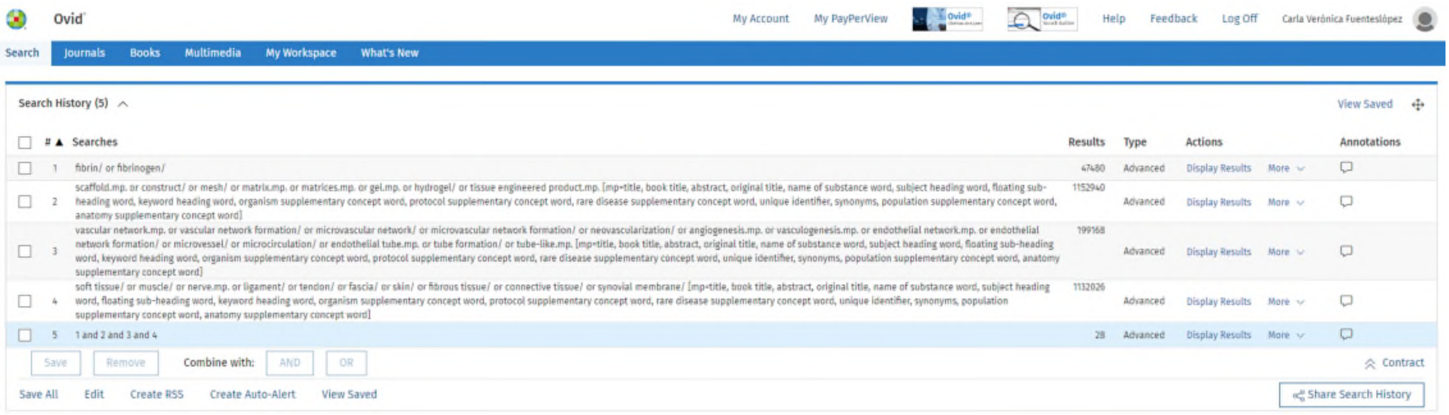

Supplement: Multimedia component 1 [file mmc1.pdf]
