# Supplementary material for: Fibrin scaffolds for angiogenesis in soft tissue models: a systematic review
Source: Bioact Mater. 2025 Dec 4;56:703–25. doi: 10.1016/j.bioactmat.2025.10.019 (PMC12747203; doi:10.1016/j.bioactmat.2025.10.019)
Supplement: Multimedia component 2 [file mmc2.pdf]

Minimum Information for Fibrin Scaffold Experiments (MIFSE) Checklist

Version: 1.0 (August 2025)

This checklist provides key reporting points for fibrin- or fibrinogen-based scaffold studies, including scaffold composition & fabrication, scaffold properties, cell pre-embedding, experimental context, functional & biological assessments, and general reporting standards.

Items in standard typeface are recommended as mandatory, when applicable, while items in *italics* are suggested.

While designed for fibrin scaffolds, the checklist can be applied to other tissue engineering scaffolds, and we encourage its use to improve reproducibility, transparency, and comparability across studies.

1. Scaffold Composition & Fabrication

Fibrinogen

- [ ] Source (*e.g.,* commercial, extracted).
- [ ] If commercially sourced, manufacturer and country of origin.
- [ ] Species of origin (*e.g.,* human, bovine).
- [ ] *Purity.*
- [ ] *Clottability.*
- [ ] Concentration with units (*e.g.,* mg/ml) and stage of the formulation at which this is used (*e.g.,* initial stock, final concentration).
- [ ] Media/buffer/solvent used.
- [ ] *Storage conditions.*

Coagulant(s)

- [ ] Type (*e.g.,* thrombin).
- [ ] Source (*e.g.,* commercial, extracted).
- [ ] If commercially sourced, manufacturer and country of origin.
- [ ] Species of Origin (*e.g.,* human, bovine).
- [ ] Concentration with units (*e.g.,* mg/ml) and stage of the formulation at which this is used (*e.g.,* initial stock, final concentration).
- [ ] Media/buffer/solvent used.
- [ ] *Storage conditions.*

Crosslinker(s)

- [ ] Type (*e.g.,* $CaCl_2$).
- [ ] Source (*e.g.,* commercial, extracted).
- [ ] If commercially sourced, manufacturer and country of origin.
- [ ] Species of Origin (*e.g.,* human, bovine).
- [ ] Concentration with units (*e.g.,* mg/ml) and stage of the formulation at which this is used (*e.g.,* initial stock, final concentration).
- [ ] Media/buffer/solvent used.
- [ ] *Storage conditions.*

Other Materials Used - the following details should be provided for each additional material and if applicable.

- [ ] Type (*e.g.,* Collagen I).
- [ ] Source (*e.g.,* commercial, extracted).
- [ ] If commercially sourced, manufacturer and country of origin.
- [ ] Species of Origin (*e.g.,* human, bovine).
- [ ] Concentration with units (*e.g.,* mg/ml) and stage of the formulation at which this is used (*e.g.,* initial stock, final concentration).
- [ ] Media/buffer/solvent used.
- [ ] *Storage conditions.*

Formulation & Fabrication

- [ ] Ratio of fibrinogen : coagulant : crosslinker : other materials.
- [ ] Method of mixing and polymerisation conditions (*e.g.,* temperature, time, order of addition).
- [ ] *Sterilisation method(s).*
- [ ] *Storage conditions.*

2. Scaffold Design & Properties

- [ ] Manufactured Object Type (*e.g.,* hydrogel, sheet, fibre, composite).
- [ ] *Dimensions and geometry (e.g., thickness, volume, pore size).*
- [ ] *Mechanical properties (e.g., stiffness, tensile strength, rheology).*
- [ ] *Degradation profile (e.g., enzymatic stability, in vitro degradation rate).*
- [ ] *Swelling ratio or water content.*

3. Cell Pre-embedding

- [ ] Specify whether cells were pre-embedded into the scaffold or not.

If cells were pre-embedded:

  - [ ] Cell line(s) used, including type (*e.g.,* primary, immortalised), species (*e.g.,* human, rat), tissue source (*e.g.,* umbilical vein), and name (*e.g.,* HUVEC).
  - [ ] Cell density or seeding concentration.
  - [ ] Passage number.
  - [ ] Encapsulation or embedding method.
  - [ ] *Preconditioning of cells (e.g., differentiation state, growth factor treatment).*

4. Experimental Context

- [ ] Study type (*e.g., in vitro, in vivo*).
- [ ] Cell culture media conditions, including culture media (*e.g.,* DMEM) , media supplements (*e.g.,* FBS, VEGF), antibiotics (*e.g.,* penicillin, streptomycin).
- [ ] Cell culture conditions (*e.g.,* CO_2, temperature, time between media changes).
- [ ] Duration of culture or observation.
- [ ] *In vivo* model used (*e.g.,* species, strain, age, sex).
- [ ] Experimental groups conditions and number of replicates.
- [ ] Controls used and number of replicates.
- [ ] Randomisation or blinding procedures.

5. Functional & Biological Assessments

- [ ] Assays performed (*e.g.,* angiogenesis, cell viability, contractility, histology, perfusion).
- [ ] Quantitative outcomes measured, including units, mean ± SD/SEM, sample size.
- [ ] Time points of measurement.
- [ ] Imaging modalities (*e.g.,* brighfield microscopy, SEM).
- [ ] Image analysis software (*e.g.,* Fiji/ImageJ) and plugins (*e.g.,* Angiogenesis Analyzer) used.
- [ ] Criteria for success or endpoint definition.
- [ ] Statistical methods used (*e.g.,* t-test, 2-way ANOVA).

6. Reporting Standards

- [ ] Ethical approval, including organisation granting approval and registration number/ID.
- [ ] Limitations acknowledged.
- [ ] Conflict of Interests statement.
- [ ] Funding sources.
- [ ] Data and materials availability.

Copyright (c) 2025 C.V. Fuenteslópez, S. Bahcevanci, V. Patrulea and H. Ye.

Please cite this checklist using **DOI: 10.5281/zenodo.16921390**
