# Supplementary material for: Fibrin scaffolds for angiogenesis in soft tissue models: a systematic review
Source: Bioact Mater. 2025 Dec 4;56:703–25. doi: 10.1016/j.bioactmat.2025.10.019 (PMC12747203; doi:10.1016/j.bioactmat.2025.10.019)
Supplement: Multimedia component 3 [file mmc3.pdf]

SUPPLEMENTARY MATERIALS

Fibrin Scaffolds for Angiogenesis in Soft Tissue Models: A Systematic Review

Carla Verónica Fuenteslópez*†[1] | Simge Bahcevanci†[1] | Viorica Patrulea[1,2,3] | Hua Ye*[1]

[1]Institute of Biomedical Engineering, Department of Engineering Science, University of Oxford, Old Road Campus Research Building, Roosevelt Drive, Oxford, OX3 7DQ, United Kingdom

[2]Institute of Pharmaceutical Sciences of Western Switzerland, University of Geneva, Geneva, 1211, Switzerland

[3]School of Pharmaceutical Sciences, University of Geneva, Geneva, 1211, Switzerland

Correspondence

*Corresponding authors: CVF & HY

Email: carla.fuenteslopez@eng.ox.ac.uk and hua.ye@eng.ox.ac.uk

† The authors contributed equally to this work.

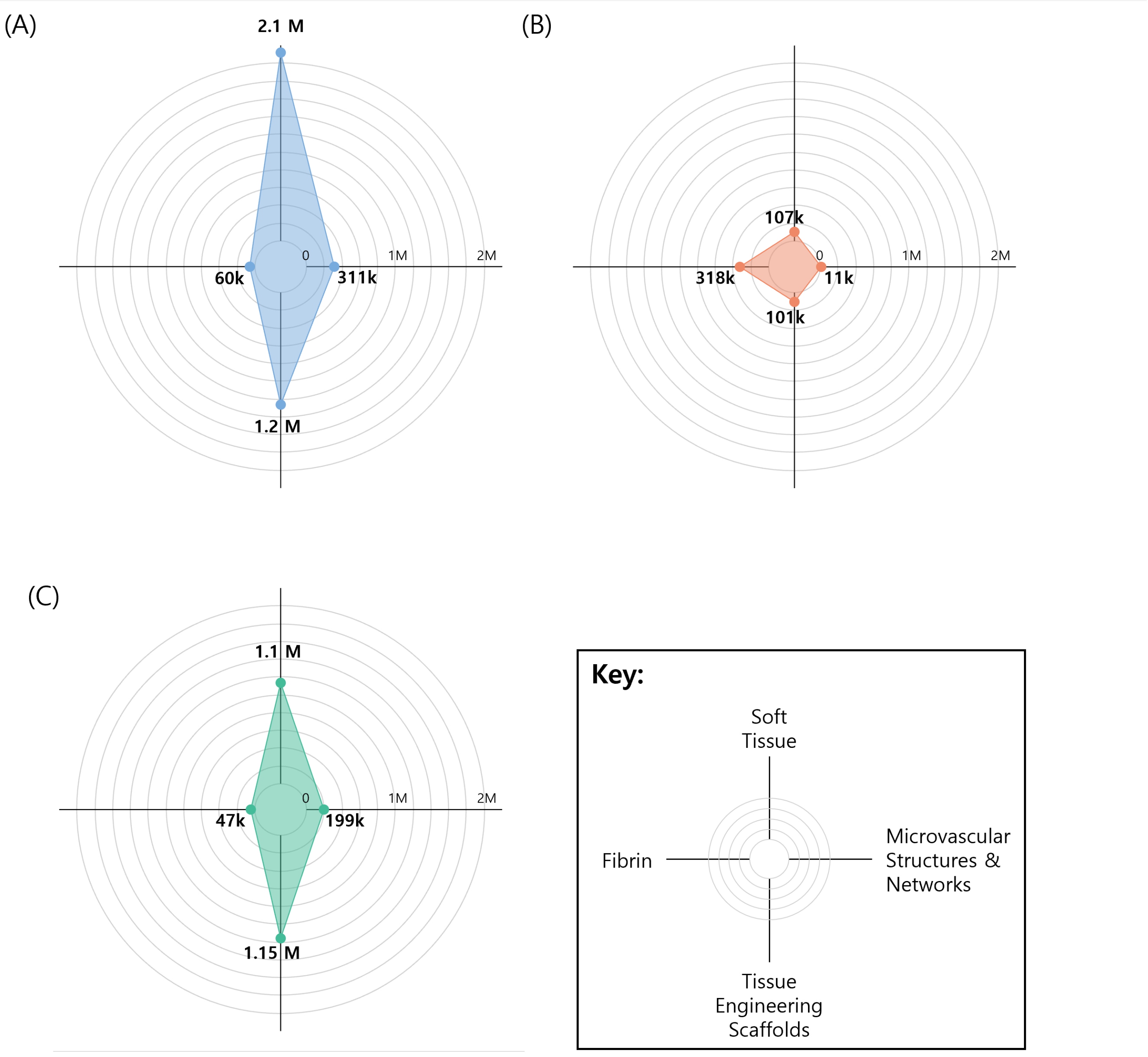

F I G U R E S1 Visual representation of the number of records found in each database when independently searching for each of the four search query components: "Soft Tissue", "Microvascular Structures and/or Network", "Tissue Engineering Scaffolds", and "Fibrin". (A) PubMed, (B) Scopus, and (C) OVID. The full search query used for each database can be found in Appendix I. Each ring represents 200,000 records.

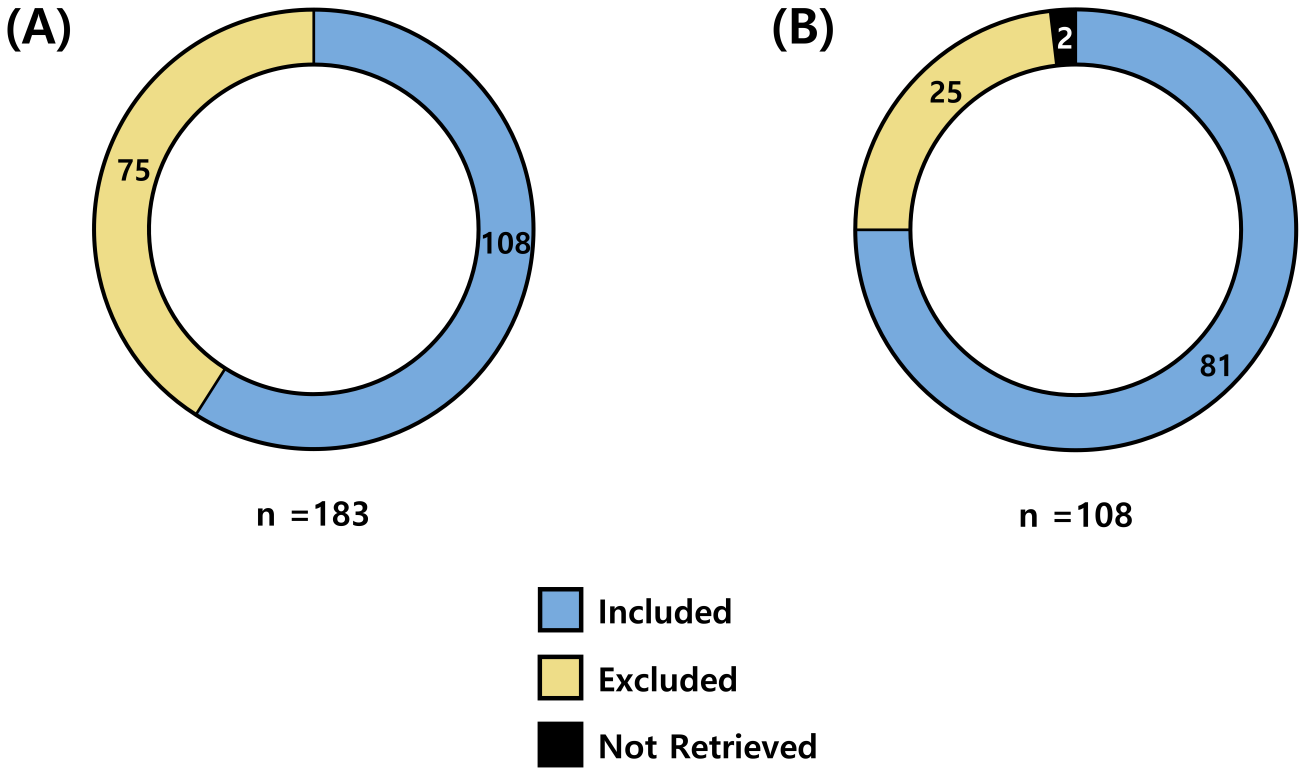

FIGURE S2 **Screening Decisions**, as agreed by the reviewers after independent binary classification of the study based on the inclusion and exclusion criteria. Two rounds of screening were conducted to evaluate the relevance of each record based on (A) title and abstract, and (B) full-text review. Two full-text articles could not be retrieved, so were excluded.

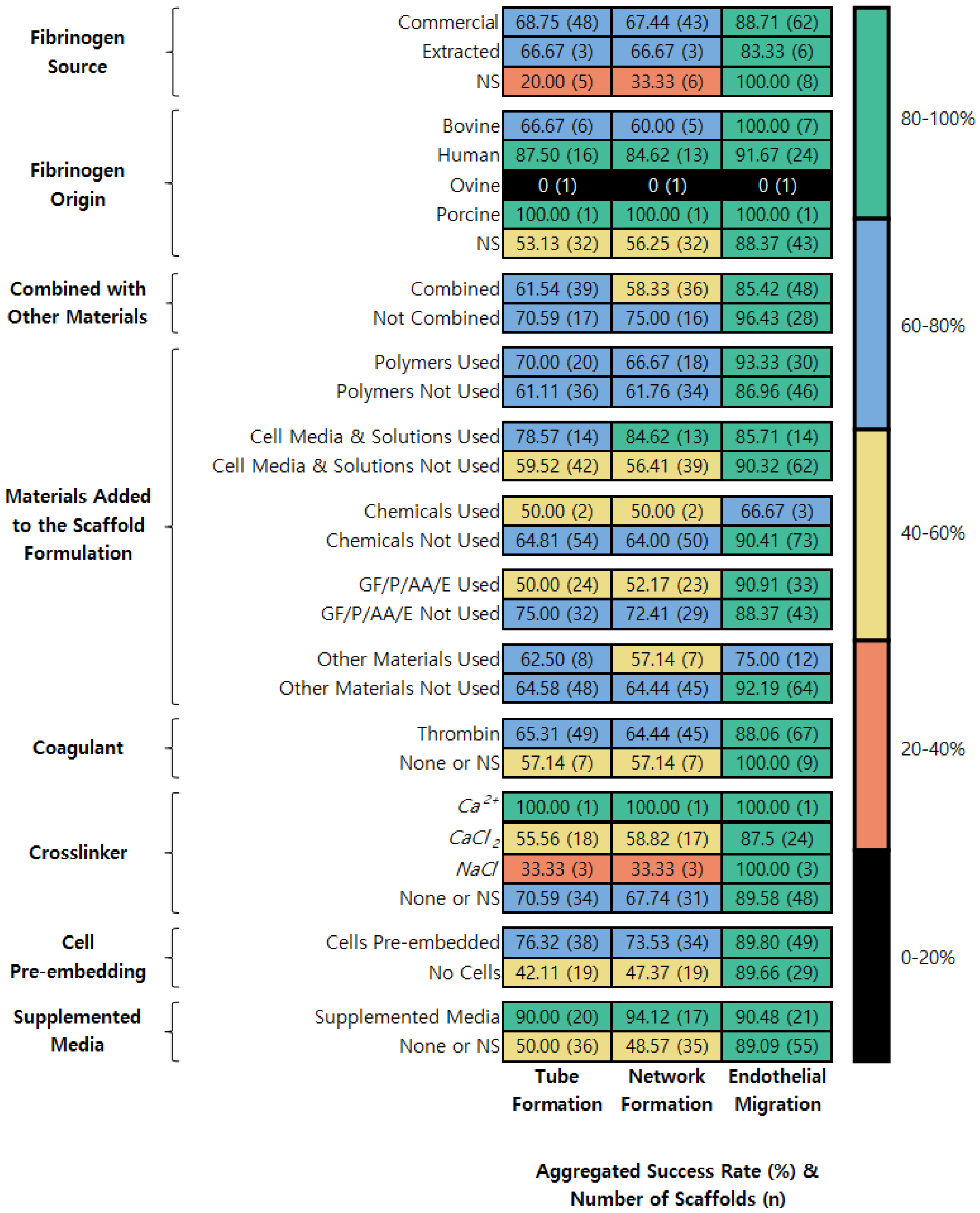

FIGURE S3 Aggregated Success Rates (%) and Number of Scaffolds Used for Success Rate Calculations (n) of Fibrin Scaffold Design Parameters. Each cell presents the percentage of successful scaffolds (aggregated success rate) followed by the number of scaffolds contributing to the calculation. Success rates were calculated as the proportion of successful scaffolds relative to the total number of successful and unsuccessful scaffolds. Only scaffolds for which study authors reported an outcome and included the specified formulation element were considered; scaffolds with no reported outcome or without the relevant element were excluded. The heatmap is colour-coded in five tiers based on the aggregated success rate: green (80–100% success), blue (60–80%), yellow (40–60%), orange (20–40%), and black (0–20%). Reported outcomes include tube formation, network formation, and endothelial cell migration. Scaffold parameters analysed were: fibrinogen source and origin, combination with other materials (and material type), use and type of coagulant and crosslinker, cell pre-embedding, and use of supplemented media. GF/P/AA/E: Growth Factors, Peptides, Amino Acids & Enzymes. NS: Not specified.

.

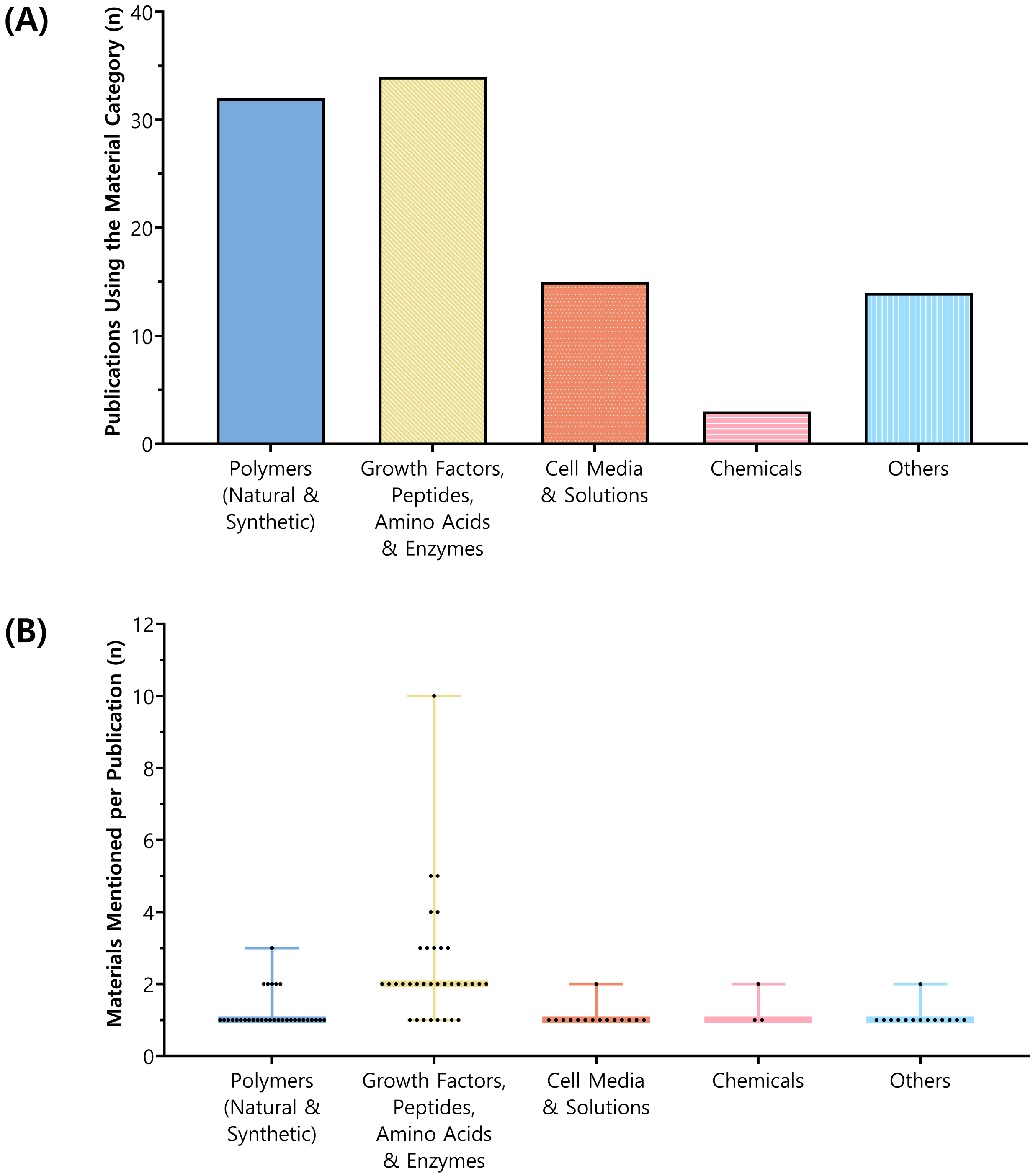

FIGURE S4 Materials Used in Combination with Fibrin, Coagulants, and Crosslinkers. (A) Number of publications using each category of material. (B) Median number of different materials used per scaffold within each category. Error bars indicate the range.

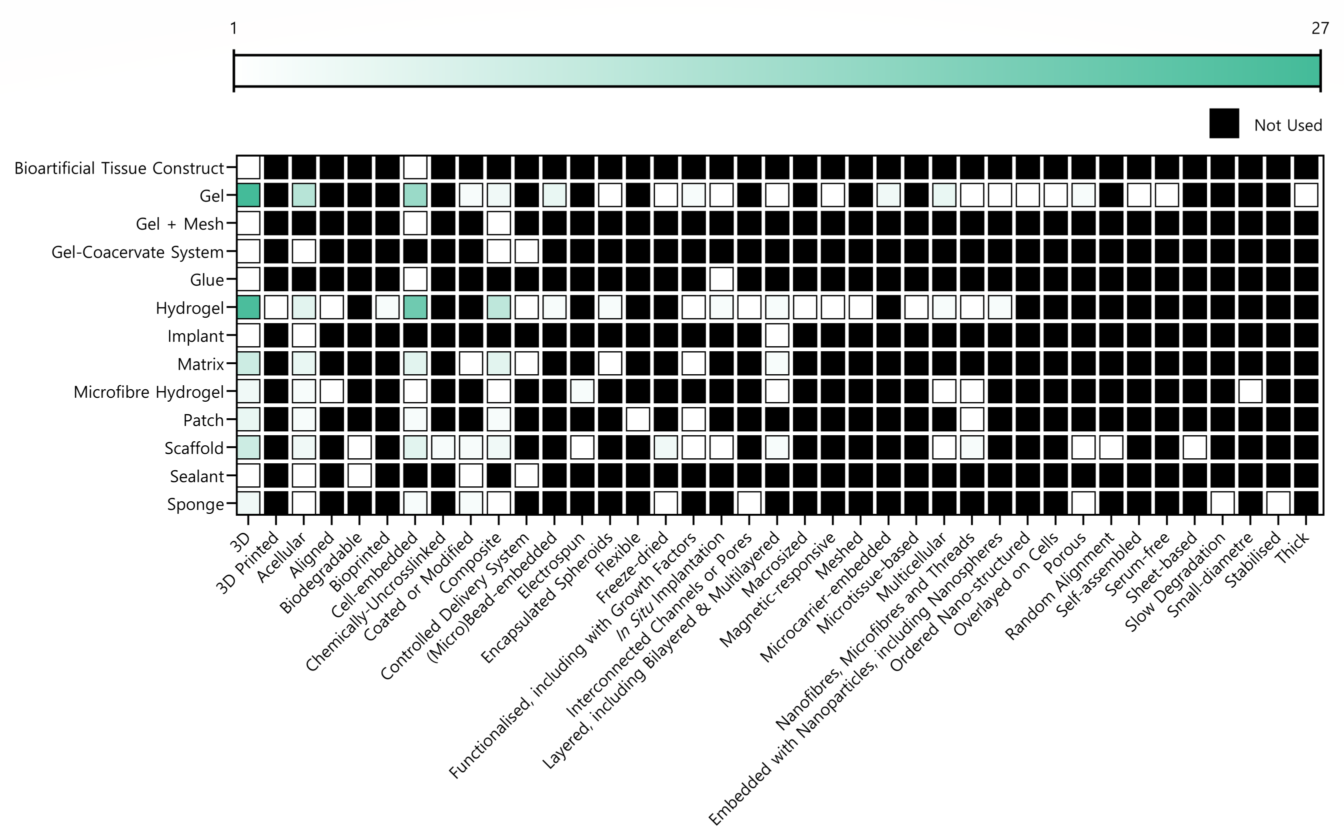

FIGURE S5 **Features Associated with the Manufactured Objects Used in the Corpus**, as described in the studies.

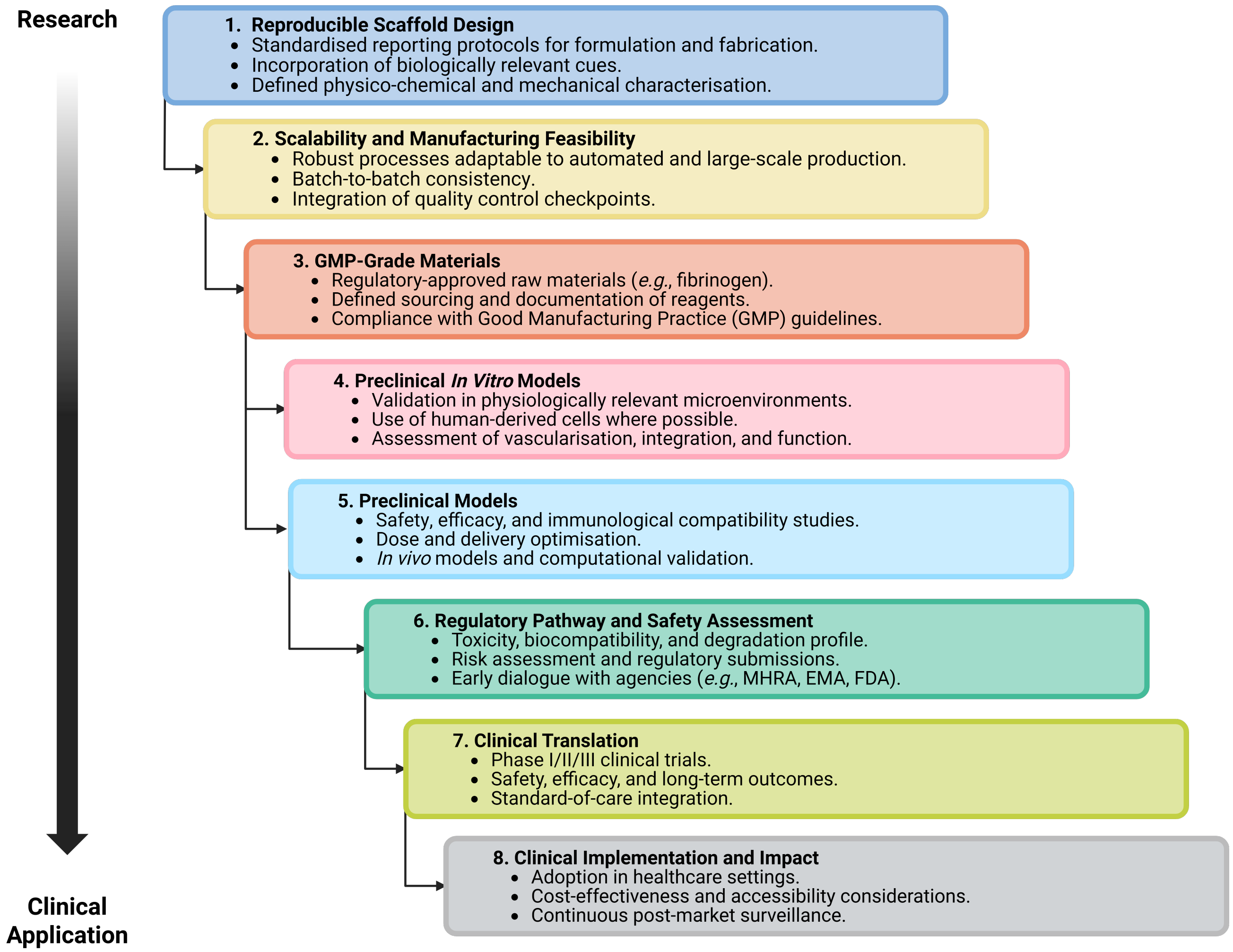

FIGURE S6 Roadmap for Fibrin Scaffolds from Reproducible Scaffold Design to Clinical Application, depicting eight key stages to improve reproducibility, allow for meaningful comparisons across studies, and advance clinical translation.

TABLE S1 Articles Excluded During the 2nd Screening Round (Full-text Screening), by reason for exclusion. [1–27]

Studies Not Retrieved	n
Full-text not available.	2

Reason for Exclusion	n
Retracted publications.	1
Non-peer-reviewed publications.	0
Non-original research articles.	2
Articles not published in English.	0
Studies do not include data and/or outcomes of endothelial formation and migration processes.	10
Studies do not contain fibrin-based scaffolds.	0
Studies do not focus on soft tissue applications.	1
Studies with exclusive use of autologous or allogenic grafts or materials.	11

TABLE S2 Studies Included in the Corpus (*n* = 81), with their corresponding bibliographical data. [28–108]

	Title	Reference	DOI	PMID
1	Fabrication of chitin-fibrin hydrogels to construct the 3D artificial extracellular matrix scaffold for vascular regeneration and cardiac tissue engineering.	Pengcheng Yang *et al.*, 2024	10.1002/jbm.a.37774	39007419
2	Vascularization of PEGylated fibrin hydrogels increases the proliferation of human iPSC-cardiomyocytes.	Ethan J Vanderslice *et al.*, 2024	10.1002/jbm.a.37662	38155509
3	External magnetic field non-invasively stimulates spinal cord regeneration in rat *via* a magnetic-responsive aligned fibrin hydrogel.	Chun-Yi Yang *et al.*, 2023	10.1088/1758-5090/acdbec	37279745
4	Chitosan-collagen-fibrinogen uncrosslinked scaffolds possessing skin regeneration and vascularization potential.	Shalini Dasgupta *et al.*, 2023	10.1002/jbm.a.37488	36573698
5	Fibrin-based cardiac patch containing neuregulin-1 for heart repair after myocardial infarction.	Tianqi Chang *et al.*, 2022	10.1016/j.colsurfb.2022.112936	36265312
6	An encapsulated fibrin-based bioartificial tissue construct with integrated macrovessels, microchannels, and capillary tubes.	Florian Helms *et al.*, 2022	10.1002/bit.28111	35485750
7	The effects of oral mucosa-derived heterotopic fibroblasts on cutaneous wound healing.	Billur Sezgin *et al.*, 2021	10.1016/j.bjps.2021.02.011	33935009
8	Vascular Network Formation on Macroporous Polydioxanone Scaffolds.	Sebastian Heene *et al.*, 2021	10.1089/ten.TEA.2020.0232	33397206
9	Fibrin hydrogels promote scar formation and prevent therapeutic angiogenesis in the heart.	Ludovic Melly *et al.*, 2020	10.1002/term.3118	32841501
10	Engineered skin graft with stromal vascular fraction cells encapsulated in fibrin-collagen hydrogel: A clinical study for diabetic wound healing.	Mohammad Ali Nilforoushzadeh *et al.*, 2020	10.1002/term.3003	31826321
11	Regenerative and durable small-diameter graft as an arterial conduit.	Morgan B Elliotti *et al.*, 2019	10.1073/pnas.1905966116	31182572
12	Effective stacking and transplantation of stem cell sheets using exogenous ROS-producing film for accelerated wound healing.	Min-Ah Koo *et al.*, 2019	10.1016/j.actbio.2019.01.019	30660002
13	Nanofiber-acellular dermal matrix as a bilayer scaffold containing mesenchymal stem cell for healing of full-thickness skin wounds.	Mohamad Javad Mirzaei-Parsa *et al.*, 2019	10.1007/s00441-018-2927-6	30338376
14	Hierarchically aligned fibrin nanofiber hydrogel accelerated axonal regrowth and locomotor function recovery in rat spinal cord injury.	Shenglian Yao *et al.*, 2018	10.2147/IJN.S159356	29844671
15	Angiogenic potential of spheroids from umbilical cord and adipose-derived multipotent mesenchymal stromal cells within fibrin gel.	Anastasiya A Gorkun *et al.* , 2018	10.1088/1748-605X/aac22d	29722292
16	Delivery of Allogeneic Adipose Stem Cells in Polyethylene Glycol-Fibrin Hydrogels as an Adjunct to Meshed Autografts After Sharp Debridement of Deep Partial Thickness Burns.	David M Burmeister *et al.*, 2018	10.1002/sctm.17-0160	29457376
17	Goat tendon collagen-human fibrin hydrogel for comprehensive parametric evaluation of HUVEC microtissue-based angiogenesis.	Rajan Narayan *et al.*, 2018	10.1016/j.colsurfb.2017.12.056	29329074
18	Adipose tissue-derived stem cells in a fibrin implant enhance neovascularization in a peritoneal grafting site: a potential way to improve ovarian tissue transplantation.	Diego D Manavella *et al.*, 2018	10.1093/humrep/dex374	29304240
19	Construction of a vascularized hydrogel for cardiac tissue formation in a porcine model.	Kp Myu Mai Ja *et al.*, 2018	10.1002/term.2634	29266858
20	Fibrinogen-modified sodium alginate as a scaffold material for skin tissue engineering.	Elena V Solovieva *et al.*, 2018	10.1088/1748-605X/aa9089	28972200
21	Experimental study on repairing skin defect by tissue-engineered skin substitute compositely constructed by adipose-derived stem cells and fibrin gel.	Rui-Xi Zeng *et al.*, 2017	No DOI Registered.	28745800
22	Emulating human microcapillaries in a multi-organ-chip platform.	Tobias Hasenberg *et al.*, 2015	10.1016/j.jbiotec.2015.09.038	26435219
23	Rapid release of growth factors regenerates force output in volumetric muscle loss injuries.	Jonathan M Grasman *et al.*, 2015	10.1016/j.biomaterials.2015.08.047	26344363
24	Endothelial Network Formation Within Human Tissue-Engineered Skeletal Muscle.	Dacha Gholobova *et al.*, 2015	10.1089/ten.TEA.2015.0093	26177063
25	Fabrication of 3-dimensional multicellular microvascular structures.	Sebastian F Barreto-Ortiz *et al.*, 2015	10.1096/fj.14-263343	25900808
26	Sequential delivery of angiogenic growth factors improves revascularization and heart function after myocardial infarction.	Hassan K Awada *et al.*, 2015	10.1016/j.jconrel.2015.03.034	25836592
27	Fibrin-based 3D matrices induce angiogenic behavior of adipose-derived stem cells.	Eunna Chung *et al.*, 2015	10.1016/j.actbio.2015.01.012	25600400
28	Long-term functional benefits of human embryonic stem cell-derived cardiac progenitors embedded into a fibrin scaffold.	Valérie Bellamy *et al.*, 2015	10.1016/j.healun.2014.10.008	25534019
29	Mesenchymal stromal cells form vascular tubes when placed in fibrin sealant and accelerate wound healing *in vivo*.	Julio J Mendez *et al.*, 2015	10.1016/j.biomaterials.2014.11.011	25433608
30	Heparin-conjugated poly(lactic-co-glycolic acid) nanospheres enhance large-wound healing by delivering growth factors in platelet-rich plasma.	Wan-Geun La *et al.*, 2015	10.1111/aor.12389	25284020
31	Tissue-engineered, hydrogel-based endothelial progenitor cell therapy robustly revascularizes ischemic myocardium and preserves ventricular function.	Pavan Atluri *et al.*, 2014	10.1016/j.jtcvs.2014.06.038	25129603
32	Mechanisms of vasculogenesis in 3D fibrin matrices mediated by the interaction of adipose-derived stem cells and endothelial cells.	Sabrina Rohringer *et al.*, 2014	10.1007/s10456-014-9439-0	25086616
33	Specific effects of fibrinogen and the γA and γ'-chain fibrinogen variants on angiogenesis and wound healing.	Elim Y L Cheung *et al.*, 2015	10.1089/ten.TEA.2014.0020	24974891
34	Long-lasting fibrin matrices ensure stable and functional angiogenesis by highly tunable, sustained delivery of recombinant $VEGF_{164}$.	Veronica Sacchi *et al.*, 2014	10.1073/pnas.1404605111	24778233
35	PlGF-MMP9-engineered iPS cells supported on a PEG-fibrinogen hydrogel scaffold possess an enhanced capacity to repair damaged myocardium.	Claudia Bearzi *et al.*, 2014	10.1038/cddis.2014.12	24525729
36	Bioengineering dermo-epidermal skin grafts with blood and lymphatic capillaries.	Daniela Marino *et al.*, 2014	10.1126/scitranslmed.3006894	24477001
37	Fibrin gel improves tissue ingrowth and cell differentiation in human immature premolars implanted in rats.	Nisarat Ruangsawasdi *et al.*, 2014	10.1016/j.joen.2013.09.022	24461412
38	*In vitro* vascularization of a combined system based on a 3D printing technique.	Xinru Zhao *et al.*, 2016	10.1002/term.1863	24399638
39	A novel *in vitro* model for microvasculature reveals regulation of circumferential ECM organization by curvature.	Sebastian F Barreto-Ortiz *et al.*, 2013	10.1371/journal.pone.0081061	24278378
40	Angiogenic endothelial cell invasion into fibrin is stimulated by proliferating smooth muscle cells.	Areck A Ucuzian *et al.*, 2013	10.1016/j.mvr.2013.06.012	23886898
41	Mutual effect of subcutaneously transplanted human adipose-derived stem cells and pancreatic islets within fibrin gel.	Suk Ho Bhang *et al.*, 2013	10.1016/j.biomaterials.2013.06.018	23827190
42	Enhanced wound vascularization using a dsASCs seeded FPEG scaffold.	David O Zamora *et al.*, 2013	10.1007/s10456-013-9352-y	23709171
43	Scaffold-based delivery of a clinically relevant anti-angiogenic drug promotes the formation of *in vivo* stable cartilage.	Matteo Centola *et al.*, 2013	10.1089/ten.TEA.2012.0455	23611597
44	An improved freeze-dried PRP-coated biodegradable material suitable for connective tissue regenerative therapy.	Makoto Horimizu *et al.*, 2013	10.1016/j.cryobiol.2013.01.006	23422785
45	Bioprinted amniotic fluid-derived stem cells accelerate healing of large skin wounds.	Aleksander Skardal *et al.*, 2012	10.5966/sctm.2012-0088	23197691
46	Adipose-derived stem cells induce vascular tube formation of outgrowth endothelial cells in a fibrin matrix.	Wolfgang Holnthoner *et al.*, 2015	10.1002/term.1620	23038666
47	Comparing scaffold-free and fibrin-based adipose-derived stromal cell constructs for adipose tissue engineering: an *in vitro* and *in vivo* study.	Femke Verseijden *et al.*, 2012	10.3727/096368912X653129	22840523
48	Bioenergetic and functional consequences of cellular therapy: activation of endogenous cardiovascular progenitor cells.	Qiang Xiong *et al.*, 2012	10.1161/CIRCRESAHA.112.269894	22723295
49	A combined synthetic-fibrin scaffold supports growth and cardiomyogenic commitment of human placental derived stem cells.	Antonella Lisi *et al.*, 2012	10.1371/journal.pone.0034284	22509287
50	Fibrin-embedded adipose derived stem cells enhance skin flap survival.	Matthias A Reichenberger *et al.*, 2012	10.1007/s12015-011-9341-8	22215322
51	Vascularization of prevascularized and non-prevascularized fibrin-based human adipose tissue constructs after implantation in nude mice.	Femke Verseijden *et al.*, 2012	10.1002/term.410	21360688
52	Advantages of hyaluronic acid as a component of fibrin sheet for care of acute wound.	Thapasimuthu V Anilkumar *et al.*, 2011	10.1016/j.biologicals.2011.01.003	21334921
53	Endothelial progenitor cells are integrated in newly formed capillaries and alter adjacent fibrovascular tissue after subcutaneous implantation in a fibrin matrix.	Oliver Bleiziffer *et al.*, 2011	10.1111/j.1582-4934.2010.01247.x	21199325
54	Fibrin acts as biomimetic niche inducing both differentiation and stem cell marker expression of early human endothelial progenitor cells.	Maria Chiara Barsotti *et al.*, 2011	10.1111/j.1365-2184.2010.00715.x	21199008

	Title (cont.)	Reference (cont.)	DOI (cont.)	PMID (cont.)
55	Vascularization of the dermal support enhances wound re-epithelialization by in situ delivery of epidermal keratinocytes.	Liana M Lugo *et al.*, 2011	10.1089/ten.tea.2010.0125	20929281
56	Coronary arteries angiogenesis in ischemic myocardium: biocompatibility and biodegradability of various hydrogels.	Xiaodong Shen *et al.*, 2009	10.1111/j.1525-1594.2009.00815.x	19681839
57	Adult human bone marrow- and adipose tissue-derived stromal cells support the formation of prevascular-like structures from endothelial cells *in vitro.*	Femke Verseijden *et al.*, 2010	10.1089/ten.TEA.2009.0106	19642855
58	The effectiveness of basic fibroblast growth factor in fibrin-based cultured skin substitute *in vivo.*	Shigeaki Inoue *et al.*, 2009	10.1097/BCR.0b013e3181a28e4b	19349876
59	Engineered adipose tissue formation enhanced by basic fibroblast growth factor and a mechanically stable environment.	Seung-Woo Cho *et al.*, 2007	10.3727/000000007783464795	17658132
60	Implantation of VEGF transfected preadipocytes improves vascularization of fibrin implants on the cylinder chorioallantoic membrane (CAM) model.	Nestor Torio-Padron *et al.*, 2007	10.1080/13645700701384116	17573620
61	*In vitro* analysis of the interactions between preadipocytes and endothelial cells in a 3D fibrin matrix.	Jörg Borges *et al.*, 2007	10.1080/13645700600935398	17573618
62	Magnetically-guided self-assembly of fibrin matrices with ordered nano-scale structure for tissue engineering.	Eben Alsberg *et al.*, 2006	10.1089/ten.2006.12.3247	17518638
63	VEGF(165) and bFGF protein-based therapy in a slow release system to improve angiogenesis in a bioartificial dermal substitute *in vitro* and *in vivo.*	I Wilcke *et al.*, 2007	10.1007/s00423-007-0194-1	17404752
64	Platelets, thrombospondin-1 and human dermal fibroblasts cooperate for stimulation of endothelial cell tubulogenesis through VEGF and PAI-1 regulation.	Sabrina Kellouche *et al.*, 2007	10.1016/j.yexcr.2006.10.023	17126831
65	Adipose precursor cells (preadipocytes) induce formation of new vessels in fibrin glue on the newly developed cylinder chorioallantoic membrane model (CAM).	Joerg Borges *et al.*, 2006	10.1080/14017450600761620	16966139
66	Peptide-matrix-mediated gene transfer of an oxygen-insensitive hypoxia-inducible factor-1alpha variant for local induction of angiogenesis.	Diana Trentin *et al.*, 2006	10.1073/pnas.0505964102	16477043
67	Control of basic fibroblast growth factor release from fibrin gel with heparin and concentrations of fibrinogen and thrombin.	Oju Jeon *et al.*, 2005	10.1016/j.jconrel.2005.03.023	16088988
68	Diffusion limits of an *in vitro* thick prevascularized tissue.	Craig K Griffith *et al.*, 2005	10.1089/ten.2005.11.257	15738680
69	Autologous stem cell transplantation for myocardial repair.	Jingbo Liu *et al.*, 2004	10.1152/ajpheart.00019.2004	15277194
70	A novel role for erythropoietin during fibrin-induced wound-healing response.	Zishan A Haroon *et al.*, 2003	10.1016/S0002-9440(10)63459-1	12937140
71	Angiogenic sprouting and capillary lumen formation modeled by human umbilical vein endothelial cells (HUVEC) in fibrin gels: the role of fibroblasts and Angiopoietin-1.	Martin N Nakatsu *et al.*, 2003	10.1016/s0026-2862(03)00045-1	12935768
72	Transplantation of autologous endothelial cells induces angiogenesis.	Valeri Chekanov *et al.*, 2003	10.1046/j.1460-9592.2003.00080.x	12687876
73	Delivery of high dose VEGF plasmid using fibrin carrier does not influence its angiogenic potency.	Alicia Jozkowicz *et al.*, 2003	10.1177/039139880302600211	12653351
74	A technique for quantitative three-dimensional analysis of microvascular structure.	Eric M Brey *et al.*, 2002	10.1006/mvre.2002.2395	11969305
75	Expression of antisense to integrin subunit beta 3 inhibits microvascular endothelial cell capillary tube formation in fibrin.	Susan M Dallabrida *et al.*, 2000	10.1074/jbc.M001446200	10922359
76	Fibrin and collagen differentially regulate human dermal microvascular endothelial cell integrins: stabilization of alphav/beta3 mRNA by fibrin1.	Xiaodong Feng *et al.*, 1999	10.1046/j.1523-1747.1999.00786.x	10594730
77	Contact-dependent inhibition of angiogenesis by cardiac fibroblasts in three-dimensional fibrin gels *in vitro* : implications for microvascular network remodeling and coronary collateral formation.	Volker Nehls *et al.*, 1998	10.1007/s004410051140	9716738
78	Biological molecule-impregnated polyester: an *in vivo* angiogenesis study.	Nancy Fournier *et al.*, 1996	10.1016/0142-9612(96)87645-9	8866027
79	A microcarrier-based cocultivation system for the investigation of factors and cells involved in angiogenesis in three-dimensional fibrin matrices *in vitro.*	Volker Nehls *et al.*, 1995	10.1007/BF01464336	8777732
80	The effect of fibroblasts, vascular smooth muscle cells, and pericytes on sprout formation of endothelial cells in a fibrin gel angiogenesis system.	Volker Nehls *et al.*, 1994	10.1006/mvre.1994.1061	7537351
81	Fibrin gel investment associated with line 1 and line 10 solid tumor growth, angiogenesis, and fibroplasia in guinea pigs. Role of cellular immunity, myofibroblasts, microvascular damage, and infarction in line 1 tumor regression.	Harold F Dvorak *et al.*, 1979	10.1093/jnci/62.6.1459	286118

TABLE S3 Overview of Key Study Details Highlighting the Heterogeneity of the Corpus. For each article, information is provided on the soft tissue type; study type (*in vitro*, *in vivo*, *ex vivo*, or clinical); endothelial cell lines, non-endothelial cell lines and *in vivo* models used; and assays employed, and the type of data reported (quantitative, qualitative, or both) for assessing endothelial formation and migration.

Study Code	Soft Tissue Type	Includes *in vitro* work	Includes *in vivo* work	Includes *ex vivo* work	Includes clinical work	Endothelial Cell Lines Used	Non-Endothelial Cell Lines Used	*In Vivo* Models Used	Assays for Evaluation of Endothelial Formation & Migration Processes	Type of Data on Endothelial Formation and Migration Processes
SR01	Cardiac Muscle	Yes	Yes	No	No	N/A	Human Amniotic Mesenchymal Stromal Cells (hAMSCs)	N/A	mRNA Expression	Quantitative
SR02	Cardiac Muscle	Yes	No	No	No	Calf Pulmonary Artery Endothelial Cells (PAECs)	Bovine (Corneal) Fibroblasts; Bovine (Retinal) Pericytes; Bovine (Pulmonary Artery) Vascular Smooth Muscle Cells (VSMCs)	N/A	Imaging	Quantitative & Qualitative
SR03	Nervous Tissue	Yes	Yes	No	No	Human Endothelial Colony Forming Cells (ECFCs)	Human Vascular Smooth Muscle Cells (vSMCs)	N/A	Confocal Imaging; Immunostaining & Immunofluorescence Staining	Quantitative & Qualitative
SR04	Skin	Yes	Yes	No	No	N/A	N/A	Lewis Rat	Brightfield Imaging; Immunohistochemistry	Quantitative & Qualitative
SR05	Cardiac Muscle	Yes	Yes	No	No	N/A	Human (Subcutaneous) Preadipocytes	White-Leghorn (Eggs) Domestic Chicken	Histology; Histomorphometric Analysis	Quantitative & Qualitative
SR06	Vascular Tissue	Yes	No	No	No	Outgrowth endothelial cells (OECs) from human peripheral blood	Human Adipose Tissue-derived Stem Cells (ASCs); Mouse C2C12 Cells	N/A	ELISA; Image Analysis; Immunoblotting; Immunostaining & Immunofluorescence Staining; Tube Formation	Quantitative & Qualitative
SR07	Skin	Yes	Yes	No	No	Human Umbilical Vein Endothelial Cells (HUVECs)	Human Adipose Tissue-derived Stromal Cells (ASCs); Human Bone Marrow-derived Stromal Cells (BMSCs)	N/A	ELISA; Histomorphometric Analysis; Imaging; Immunocytochemistry	Quantitative & Qualitative
SR08	Microvasculature	Yes	No	No	No	Red Fluorescent Protein-expressing Human Umbilical Vein Endothelial Cells (RFP-HUVECs)	Human Adipose Tissue-derived Stem Cells (ASCs)	N/A	Fluorescent Imaging; Image Analysis	Quantitative & Qualitative
SR09	Cardiac Muscle	No	Yes	No	No	N/A	Human Alveolar Periosteal Cells (HAPCs)	BALB/c-nu/nu Mouse	Cytology; Histology; Immunohistochemistry	Qualitative
SR10	Skin	Yes	No	No	Yes	Canine (External Jugular Vein) Endothelial Cells (ECs)	Canine (Carotid Artery) Smooth Muscle Cells (SMCs)	N/A	Angiogenesis Assay; Co-culture Agregate Assay; Histology; Immunostaining & Immunofluorescence Staining	Quantitative & Qualitative
SR11	Vascular Tissue	Yes	Yes	No	No	N/A	Human Adipose-derived Stromal Cells (hADSCs); Human Umbilical Cord Multipotent Mesenchymal Stromal Cells (hUC MMSCs)	N/A	3D Angiogenesis Assay; Imaging; Immunocytochemical Staining; Morphometeric Analysis; RT-PCR	Quantitative & Qualitative
SR12	Skin	Yes	Yes	No	No	Bovine Aortic Endothelial Cells (BAECs)	Skin Fibroblasts (SF)	N/A	Angiogenesis Assay; Histology; Imaging; RT-PCR	Quantitative & Qualitative
SR13	Skin	Yes	Yes	No	No	Human Embryonic Stem Cell-derived Endothelial Cells (hESC-ECs)	Human Embryonic Stem Cell-derived Smooth Muscle Cells (hESC-SMCs)	Yorkshire Pig Swine; NOD/SCID/γc$^{-/-}$ Mouse	Histology; Imaging; Immunostaining & Immunofluorescence Staining; Tube Formation	Quantitative & Qualitative
SR14	Spinal Cord Tissue	No	Yes	No	No	Human Lymphatic Endothelial Cells (hLECs); Human Dermal Microvascular Endothelial Cells (HDMECs)	Human (Foreskin) Dermal Fibroblasts (DFs)	nu/nu Rat	Image Analysis; Imaging; Immunostaining & Immunofluorescence Staining	Qualitative
SR15	Vascular Tissue	Yes	No	No	No	N/A	N/A	CD-1 Mouse	Histology	Quantitative & Qualitative
SR16	Skin	Yes	Yes	No	No	Human Umbilical Vein Endothelial Cells (HUVECs)	Human Amniotic Fluid-derived Stem Cells (AFSs); Human Bone Marrow-derived Mesenchymal Stem Cells (MSCs)	nu/nu Mouse	Histology; Immunohistochemistry; Migration Assay	Quantitative & Qualitative
SR17	Skin	Yes	Yes	No	No	N/A	Human Adult Dermal Fibroblasts (HDFa)	Swiss Albino Mouse	Histology; Immunohistochemistry	Quantitative & Qualitative
SR18	Ovarian	No	Yes	No	No	N/A	Human Adipose-derived Stromal Cells (ASCs)	Athymic Nude Mouse	ELISA; Histology; Image Analysis; Imaging	Quantitative & Qualitative
SR19	Cardiac Muscle	No	Yes	No	No	N/A	Human Embryonic Stem Cell-derived Cardiomyocytes (hESC-CMs); Human Induced Pluripotent Stem Cell-derived Cardiomyocytes (hiPSC-CMs); Human Dermal Fibroflasts (HDF)	Yorkshire Pig Swine	Histology; Image Analysis; Imaging; Immunostaining & Immunofluorescence Staining; RT-PCR	Quantitative & Qualitative
SR20	Skin	Yes	Yes	No	No	Porcine Aortic Endothelial Cells (PAOECs)	Mouse NIH/3T3 Fibroblasts; Porcine Epicardial Adipose Tissue-derived Fibroblasts; Porcine Myocardial Fibroblasts	N/A	Angiogenesis Assay	Quantitative & Qualitative
SR21	Skin	No	Yes	No	No	N/A	Human Fibroblasts	C57BL/6 Mouse	Immunohistochemistry	Quantitative & Qualitative
SR22	Vascular Tissue	Yes	No	No	No	N/A	Rat Aortic Vascular Smooth Muscle Cells (VSMCs)	Sprague-Dawley Rat	Immunohistochemistry	Quantitative & Qualitative
SR23	Skeletal Muscle	Yes	Yes	No	No	N/A	Porcine Subcutaneous Adipose-derived Stem Cells (ASCs)	Yorkshire Pig Swine	Immunohistochemistry; Western Blot	Quantitative & Qualitative
SR24	Skeletal Muscle	Yes	No	No	No	Human Umbilical Vein Endothelial Cells (HUVECs)	Human Dermal Fibroblasts	N/A	Image Analysis	Quantitative & Qualitative
SR25	Microvasculature	Yes	No	No	No	N/A	Human Bone Marrow-derived Mesenchymal Stem Cells (hMSCs)	BALB/c Nude Mouse	Immunohistochemistry	Qualitative
SR26	Cardiac Muscle	Yes	Yes	Yes	No	Green Fluorescent Protein-expressing Human Umbilical Vein Endothelial Cells (GFP-HUVECs)	Human Adipose-derived Stromal Cells (ASCs)	N/A	Fluorescent Imaging; Imaging; Quantitative Real-Time RT-PCR	Quantitative & Qualitative
SR27	Vascular Tissue	Yes	No	No	No	Green Fluorescent Protein-expressing Human Umbilical Vein Endothelial Cells (GFP-HUVECs)	Human Muscle Cells	N/A	Angiogenesis Assay	Quantitative & Qualitative
SR28	Cardiac Muscle	Yes	No	No	No	N/A	Mouse T17b Embryonal Endothelial Progenitor Cells (eEPCs)	Lewis Rat	Immunohistochemistry	Quantitative & Qualitative
SR29	Skin	Yes	Yes	No	No	N/A	Human Adipose Stromal Vascular Fraction (SVF)-derived Preadipocytes	Athymic Mouse	Immunohistochemistry	Quantitative & Qualitative
SR30	Skin	Yes	Yes	No	No	Human Adipose Stromal Vascular Fraction (SVF)-derived Endothelial Cells	Human Dermal Fibroblasts	N/A	Immunostaining & Immunofluorescence Staining	Quantitative & Qualitative
SR31	Cardiac Muscle	Yes	Yes	No	No	N/A	Human Discarded Burn Skin Adipose-derived Stem Cells (dsASCs)	Rowett Nude Rat	Histology; Immunochemistry	Quantitative & Qualitative
SR32	Vascular Tissue	Yes	No	No	No	N/A	Mouse Green Fluorescent Protein-labelled Adipose-derived Stem Cells (GFP-ADSCs)	GFP Transgenic Mouse; Sprague-Dawley Rat	Immunostaining & Immunofluorescence Staining	Quantitative
SR33	Skin	Yes	Yes	No	No	Human Dermal Microvascular Endothelial Cells (HMEC-1)	N/A	N/A	Fluorescent Imaging	Quantitative & Qualitative
SR34	Soft Tissue (Unspecified)	Yes	Yes	No	No	Human Endothelial Colony-Forming Cells (ECFCs)	Human Vascular Smooth Muscle Cells (vSMCs); Human Placental Pericytes	N/A	Imaging; Immunostaining & Immunofluorescence Staining	Qualitative
SR35	Cardiac Muscle	Yes	Yes	No	No	Human Umbilical Vein Endothelial Cells (HUVECs)	Human Endothelial Progenitor Cells (EPCs) from Peripheral Blood Mononuclear Cell-derived (PBMC)	N/A	Tube Formation	Quantitative & Qualitative
SR36	Skin	Yes	Yes	No	No	Human Dermal Microvascular Endothelial Cells (HDMECs)	N/A	White Yorkshire Pig Swine	Northern Blot Hybridisation; RNA Isolation	Quantitative
SR37	Dental Pulp	No	Yes	No	No	N/A	N/A	Sewall Wright Inbred Strain 2 Guinea Pig	Imaging; Immunostaining & Immunofluorescence Staining	Qualitative
SR38	Vascular Tissue	Yes	No	No	No	N/A	N/A	Sprague-Dawley Nude Crl: NIH-Foxn1^{mu} Rat; Sprague-Dawley RjHan:SD Rat	Immunochemistry	Quantitative & Qualitative
SR39	Vascular Tissue	Yes	No	No	No	N/A	Human Adipose-derived Stem Cells (ASCs)	N/A	Calcein AM Staining; Imaging	Quantitative & Qualitative
SR40	Vascular Tissue	Yes	No	No	No	N/A	Rat H9c2 Cells	C57BL/6 Mouse	Immunohistochemistry	Quantitative & Qualitative
SR41	Skin	Yes	Yes	No	No	N/A	Rat DiI-labelled Fluorescent Adipose-derived Stem Cells (ADSCs)	Lewis Rat	Immunochemistry	Qualitative
SR42	Skin	Yes	Yes	No	No	Human Umbilical Vein Endothelial Cells (HUVECs)	Human Skin Dermal Fibroblasts (sFb); Human Immortalised N-TERT Epidermal Keratinocytes; Human Mesenchymal Stem Cells (MSCs); Human Subcutaneous Adipose-derived Stromal Cells (sadSC)	Unspecified Mouse	Immunostaining & Immunofluorescence Staining	Quantitative & Qualitative
SR43	Skin	Yes	Yes	Yes	No	Human Umbilical Vein Endothelial Cells (HUVECs)	N/A	Swiss Albino Mouse	3D Angiogenesis Assay; Semi qRT-PCR	Quantitative & Qualitative
SR44	Connective Tissue	Yes	Yes	No	No	N/A	N/A	Athymic Mouse	ELISA; Histology; Immunostaining & Immunofluorescence Staining; RT-PCR	Quantitative & Qualitative
SR45	Skin	Yes	Yes	No	No	Human Dermal Microvascular Endothelial Cells (HDMVECs)	Human Preadipocytes	N/A	Immunostaining & Immunofluorescence Staining; [illegible]	Quantitative & Qualitative
SR46	Vascular Tissue	Yes	No	No	No	Human Umbilical Vein Endothelial Cells (HUVECs)	N/A	Crl:CD1 Foxn1^{nu} Nude Mouse; Sprague-Dawley Rat; BALB/c nu/nu Nude Mouse; CB.17 SCID Mouse	ELISA; Histology; Imaging; Immunostaining & Immunofluorescence Staining	Quantitative & Qualitative
SR47	Adipose Tissue	Yes	Yes	No	No	N/A	Human I6 Embryonic Stem Cell (ESC)-derived Cardiac Progenitors	N/A	Histology; Immunohistochemistry	Qualitative
SR48	Cardiac Muscle	Yes	Yes	No	No	Human Microvascular Endothelial Cells (HMECs)	N/A	N/A	Confocal Imaging	Qualitative
SR49	Cardiac Muscle	Yes	No	No	No	Human Umbilical Vein Endothelial Cells (HUVECs); Green Fluorescent Protein-expressing Human Umbilical Vein Endothelial Cells (GFP-HUVECs)	Human Endothelial Progenitor Cells (EPCs) from Peripheral Blood Mononuclear Cell-derived (PBMC); Human Adipose-derived Stem Cells (ASCs)	N/A	Angiogenesis Protein Array; Immunostaining & Immunofluorescence Staining	Quantitative & Qualitative
SR50	Skin	No	Yes	No	No	N/A	Human Adipose-derived Mesenchymal Stromal Cells (ADSCs)	C57/B6 Mouse	ELISA; Fluorescence-Activated Cell Sorting; Histology; Immunohistochemistry; Quantitative Real-Time RT-PCR	Qualitative
SR51	Adipose Tissue	Yes	Yes	No	No	N/A	Human Adipose-derived Stem Cells (hADSCs); Rat Islets	Athymic Mouse	Immunohistochemistry; Proteome Profiler Human Angiogenesis Antibody Array	Quantitative & Qualitative
SR52	Skin	No	Yes	No	No	N/A	Rat Adipose-derived Mesenchymal Stem Cells (ADSCs); Normal Human Epidermal Keratinocytes (NHEK)	Wistar Rat	Histomorphometric Analysis; Immunohistochemistry	Quantitative & Qualitative
SR53	Fibrovascular Tissue	Yes	Yes	No	No	Human Umbilical Vein Endothelial Cells (HUVECs)	Human Embryonic Kidney 293 Cells (293T3)	BALB/c Mouse	Histology; Immunostaining & Immunofluorescence Staining; Immunohistochemistry	Quantitative & Qualitative

Study Code (cont.)	Soft Tissue Type (cont.)	Includes *in vitro* work (cont.)	Includes *in vivo* work (cont.)	Includes *ex vivo* work (cont.)	Includes clinical work (cont.)	Endothelial Cell Lines Used (cont.)	Non-Endothelial Cell Lines Used (cont.)	*In Vivo* Models Used *(cont.)*	Assays for Evaluation of Endothelial Formation & Migration Processes (cont.)	Type of Data on Endothelial Formation and Migration Processes (cont.)
SR54	Vascular Tissue	Yes	No	No	No	Human Endothelial Colony-Forming Cells (ECFCs)	N/A	Fox Chase SCID Beige Mouse	Histology; Imaging; Immunostaining & Immunofluorescence Staining	Quantitative & Qualitative
SR55	Skin	No	Yes	No	No	Human Umbilical Vein Endothelial Cells (HUVECs)	Human Monocytes derived from Peripheral Blood Mononuclear Cell-derived (PBMC)	CD1 nu/nu Mouse	Immunostaining & Immunofluorescence Staining	Quantitative & Qualitative
SR56	Cardiac Muscle	No	Yes	No	No	Human Umbilical Vein Endothelial Cells (HUVECs)	Baboon Smooth Muscle Cells (SMCs)	Sprague-Dawley Rat	Confocal Imaging; ELISA	Quantitative & Qualitative
SR57	Vascular Tissue	Yes	No	No	No	Human Umbilical Vein Endothelial Cells (HUVECs)	N/A	WAG/RijHsd Rat	Angiogenesis Assay; Tube Formation	Quantitative & Qualitative
SR58	Skin	No	Yes	No	No	Calf Calf Pulmonary Artery-derived Endothelial Cells; Bovine Brain Capillary Endothelial Cells (BBE); Bovine Adrenocortical Microvascular Endothelial Cells (ACE)	Bovine Vascular Smooth Muscle Cells (vSMCs); Bovine Corneal Fibroblasts; Bovine Retinal Pericytes	N/A	Angiogenesis *In Situ*	Qualitative
SR59	Adipose Tissue	No	Yes	No	No	N/A	Human Dermal Fibroblasts; Human Epidermal Keratinocytes	Athymic Mouse	Imaging; Immunohistochemistry	Qualitative
SR60	Soft Tissue (Unspecified)	No	Yes	No	No	N/A	Rat Endothelial Progenitor Cells (EPCs) from Bone Marrow Mononuclear Cells; Rat Green Fluorescent Protein-expressing Endothelial Progenitor Cells (GFP-EPCs) from Bone Marrow Mononuclear Cells	Wistar Rat	Histochemistry; Histology; Immunostaining & Immunofluorescence Staining	Quantitative & Qualitative
SR61	Skin	Yes	No	No	No	Sheep Jugular Vein-derived Endothelial Cells	N/A	Unspecified Sheep	Immunostaining & Immunofluorescence Staining; Transmission Electron Microscopy	Quantitative & Qualitative
SR62	Soft Tissue (Unspecified)	Yes	No	No	No	Green Fluorescent Protein-expressing Human Umbilical Vein Endothelial Cells (GFP-HUVECs)	Human Adipose Tissue-derived Mesenchymal Stem Cells (AD-hMSCs)	N/A	Dextran Assay; Fluorescent Imaging; Confocal Imaging; Image Analysis	Quantitative & Qualitative
SR63	Skin	No	Yes	No	No	N/A	N/A	nu/nu Mouse	Histology; Immunohistochemistry; Morphometeric Analysis	Quantitative & Qualitative
SR64	Skin	Yes	No	No	No	N/A	N/A	Fischer 344 Rat	Immunohistochemistry	Quantitative
SR65	Adipose Tissue	No	Yes	No	No	N/A	Human Adipose Tissue-derived Stem Cells (ASCs)	SCID Mouse	Immunohistochemistry	Quantitative & Qualitative
SR66	Skin	Yes	Yes	No	No	N/A	N/A	New Zealand White European Rabbit	Histopathology	Quantitative
SR67	Skeletal Muscle	Yes	Yes	No	No	N/A	Porcine Bone Marrow-derived Mesenchymal Stem Cells (MSCs)	Yorkshire Pig Swine	Histology; Quantitative Real-Time RT-PCR	Quantitative & Qualitative
SR68	Vascular Tissue	Yes	No	No	No	N/A	N/A	New Zealand White European Rabbit	Alkaline Phosphatase Assay	Quantitative & Qualitative
SR69	Cardiac Muscle	Yes	Yes	No	No	Human Umbilical Vein Endothelial Cells (HUVECs)	Rat PC12 Cells	Sprague-Dawley Rat	Immunohistochemistry	Quantitative & Qualitative
SR70	Skin	No	Yes	No	No	Green Fluorescent Protein-expressing Human Umbilical Vein Endothelial Cells (GFP-HUVECs)	N/A	BALB/c-nu Mouse	Immunostaining & Immunofluorescence Staining	Qualitative
SR71	Vascular Tissue	Yes	No	No	No	N/A	N/A	Sprague-Dawley Crl:CD Rat	Histology	Qualitative
SR72	Cardiac Muscle	No	Yes	No	No	N/A	N/A	Sprague-Dawley Rat	Immunocytochemistry; Immunostaining & Immunofluorescence Staining; Confocal Imaging	Quantitative & Qualitative
SR73	Soft Tissue (Unspecified)	Yes	Yes	No	No	N/A	Green Fluorescent Protein-expressing Human Preadipocytes	White-Leghorn (Eggs) Domestic Chicken	Histomorphometric Analysis	Quantitative & Qualitative
SR74	Skeletal Muscle	No	Yes	No	No	N/A	Rat Adipose-derived Stem Cells (ADSCs)	N/A	Histology; Immunostaining & Immunofluorescence Staining	Qualitative
SR75	Vascular Tissue	Yes	No	No	No	Human Dermal Microvascular Endothelial Cells (HMEC-1)	Human Dermal Fibroblasts	N/A	Tube Formation	Quantitative & Qualitative
SR76	Skin	Yes	Yes	No	No	Human Umbilical Vein Endothelial Cells (HUVECs)	Neonatal Mouse Cardiomyocyte-derived iPS Cells (iPS); Mouse MMP9-secreting iPS Cells (MiPS); Mouse PIGF-secreting iPS Cells (PiPS)	NOD-SCID Mouse	Angiogenesis Assay; Fluorescence-Activated Cell Sorting; Histology; Immunostaining & Immunofluorescence Staining; Quantitative Real-Time RT-PCR	Quantitative & Qualitative
SR77	Cardiac Muscle	Yes	No	No	No	N/A	Mouse C2C12 Cells	SCID Nude Hairless Outbred Mouse	Angiogenesis Assay; Histology; Immunohistochemistry	Quantitative & Qualitative
SR78	Vascular Tissue	No	Yes	No	No	N/A	Human Dermis-derived Fibroblasts; Human Oral Mucosa-derived Fibroblasts	Nude Mouse	Histology; Migration Assay	Qualitative
SR79	Vascular Tissue	Yes	No	No	No	Human Umbilical Vein Endothelial Cells (HUVECs); Green Fluorescent Protein-expressing Human Umbilical Vein Endothelial Cells (GFP-HUVECs)	Cardiomyocytes from Human Induced Pluripotent Stem Cells (CM-iPSCs)	N/A	Fluorescent Imaging	Quantitative & Qualitative
SR80	Vascular Tissue	Yes	No	No	No	Human Umbilical Vein Endothelial Cells (HUVECs)	Human Adipose Tissue-derived Stem Cells (ASCs)	Athymic Nude Mouse	Histology; Immunohistochemistry; Immunostaining & Immunofluorescence Staining	Qualitative
SR81	Vascular Tissue	No	Yes	No	No	N/A	Human Neonatal Foreskin Keratinocytes; Human Immortalised HaCaT Keratinocytes; Mouse 3T3-J2 Fibroblasts	Athymic nu/nu Mouse	Histology; Immunohistochemistry	Quantitative & Qualitative

TABLE S4 Overview of Key Scaffold Formulation Details Highlighting the Heterogeneity of the Corpus. For each article, information is provided on the fibrin, coagulant, and crosslinker used (including concentration and formulation stage); the incorporation of additional materials beyond fibrin, coagulants, and crosslinkers, categorised by type (Polymers; Growth Factors, Peptides, Amino Acids & Enzymes; Cell Media & Solutions; Chemicals; and Other Materials) and specifying the number of distinct materials of each type included in the formulation; the type of manufactured object and its associated features; whether cells were pre-embedded within the scaffold or not; and the factors (with concentrations) added to the standard cell medium.

Study Code	Fibrin Concentration and Formulation Stage	Coagulant, with Concentration and Formulation Stage	Crosslinker, with Concentration and Formulation Stage	Were Additional Materials Used in the Formulation?	Number of Different Polymers (Natural & Synthetic) Used	Number of Different Growth Factors, Peptides, Amino Acids & Enzymes Used	Number of Different Cell Media & Solutions Used	Number of Different Chemicals Used	Number of Different Materials Used (Others)	Manufactured Object	Manufactured Object Features	Were Cells Pre-embedded into the Scaffold?	Factors Added to the Standard Cell Medium with Concentrations
SR01	Not Specified	Not Used or Non Specified	Not Used or Non Specified	Yes	1	3	N/A	N/A	N/A	Scaffold	3D; Bilayered; Biodegradable; Randomly Oriented Nanofibres; Cell-embedded	Yes	N/A
SR02	10 mg/ml (Final)	Thrombin (11 µl at 100 U/ml; Stock)	*NaCl* (NS; Not Specified)	Yes	N/A	3	N/A	N/A	1	Gel	3D; Coated; Microcarrier-embedded; Cell-embedded	Yes	N/A
SR03	Not Specified	Thrombin (NS; Not Specified)	Not Used or Non Specified	Yes	1	N/A	N/A	N/A	N/A	Hydrogel	3D; Microfibre-embedded; Cell-embedded	Yes	VEGF (50 ng/ml)
SR04	2 wt% (Stock)	Thrombin (10 U/ml; Stock)	$CaCl_2$ (50 mM; Stock)	No	N/A	N/A	N/A	N/A	N/A	Implant	3D; Bilayered; Acellular	No	N/A
SR05	Not Specified	Thrombin (500 U/ml; Stock)	Not Used or Non Specified	Yes	N/A	4	N/A	N/A	N/A	Matrix	3D; Composite; Cell-embedded	Yes	Aprotinin (200 U/ml); Aprotinin (200 U/ml) + hr-bFGF (50 ng/ml) + hr-VEGF (40 ng/ml)
SR06	Not Specified	Thrombin (NS; Not Specified)	Not Used or Non Specified	Yes	N/A	N/A	1	N/A	1	Hydrogel	3D; Cytodex Bead-embedded; Cell-embedded	Yes	Aprotinin (100 KIU/ml)
SR07	20 mg/ml (Stock)	Thrombin (4 U/ml; Stock)	Not Used or Non Specified	No	N/A	N/A	N/A	N/A	N/A	Hydrogel	Spheroid-embedded; 3D; Cell-embedded	Yes	VEGF (50 ng/ml)
SR08	10 mg/ml (Not Specified)	Thrombin (50 U/ml; Stock)	$CaCl_2$ (150 mM; Stock)	Yes	N/A	2	1	N/A	N/A	Bioartificial Tissue Construct	3D; Cell-embedded	Yes	Aprotinin (100 U/ml) + L-Ascorbic Acid-2-Phosphate (50 µg/ml) + FGF (40 ng/ml) + VEGF (40 ng/ml)
SR09	36.59 or 23.59 mg/ml (Stock)	Thrombin (223 kIU/ml or 144.6 kIU/ml; Stock)	Not Used or Non Specified	Yes	1	N/A	N/A	N/A	N/A	Sponge	3D; Coated; and either Acellular or Cell-embedded	Both	N/A
SR10	2.5 mg/ml (Stock)	Thrombin (2.5 U/ml; Final)	Not Used or Non Specified	Yes	1	N/A	1	N/A	N/A	Hydrogel	3D; Bilayered; Cell-embedded; Cell Aggregate-embedded	Yes	Amphotericin (0.25 µg/ml) + Aprotinin (100 kIU/ml) + Heparan Sulfate (5 U/ml); Amphotericin (0.25 µg/ml) + Aprotinin (100 kIU/ml) + Heparan Sulfate (5 U/ml) + Methocel Solution (20%)
SR11	3 mg/ml (Final)	Thrombin (30 U/ml; Stock)	$CaCl_2$ (100 M; Stock)	Yes	1	N/A	N/A	N/A	N/A	Hydrogel	3D; Encapsulated Spheroids; Cell-embedded	Yes	N/A
SR12	10 mg/ml (Final)	Thrombin (50 U/ml; Final)	Not Used or Non Specified	Yes	N/A	1	N/A	N/A	1	Hydrogel	3D; Embedded Beads; Cell-embedded	Yes	Angiopoietin-1 (1-125 ng/ml) + bFGF (25 ng/ml) + TGF-β (0.5-2 ng/ml) + VEGF (25 ng/ml)
SR13	8 mg/ml (Final)	Thrombin (2 U/ml; Final)	Ca^{2+} (2.5 mmol/L; Final)	Yes	N/A	N/A	N/A	1	N/A	Patch	3D; Cell-embedded	Yes	N/A
SR14	0.02 (Stock)	Thrombin (100 IU/ml; Final)	Not Used or Non Specified	No	N/A	N/A	N/A	N/A	N/A	Hydrogel	3D; Cell-embedded	Yes	N/A
SR15	0.2 mg/ml (Stock)	Thrombin (0.2 ml; Stock)	*NaCl* (0.009; Stock)	Yes	3	2	2	N/A	N/A	Matrix	3D; Composite; Acellular	No	N/A
SR16	Not Specified	Thrombin (20 µl; Stock)	Not Used or Non Specified	Yes	1	N/A	N/A	N/A	N/A	Hydrogel	3D; Bioprinted; Cell-embedded	Yes	N/A
SR17	20 mg/ml (Final)	Thrombin (1250 U/ml; Not Specified)	$CaCl_2$ (275 mM; Not Specified)	Yes	2	N/A	N/A	N/A	N/A	Scaffold	3D; Chemically-Uncrosslinked; Freeze-dried; Composite; and either Acellular or Cell-embedded	Both	N/A
SR18	Not Specified	Not Used or Non Specified	Not Used or Non Specified	No	N/A	N/A	N/A	N/A	N/A	Gel	3D; Spheroid-embedded; Acellular	No	Dexamethasone (1 µM) + Foetal Calf Serum (10%) + Gentamycin (0.5%) + Indomethacin (0.2 mM) + Insulin (0.01 mg/ml) + 3-isobutyl- l-methyl-xanthine (0.5 mM)
SR19	1 mg/ml (Not Specified)	Thrombin (0.3 U/ml; Not Specified)	Not Used or Non Specified	Yes	1	2	N/A	N/A	N/A	Hydrogel	3D; Macrosized; Composite; Cell-embedded	Yes	N/A
SR20	Not Specified	Thrombin (NS; Not Specified)	Not Used or Non Specified	Yes	N/A	10	1	N/A	N/A	Gel	3D; Serum-free; Microcarrier-embedded; Cell-embedded	Yes	Insulin (1 µg/ml) + Penicillin (100 U/ml) + Selenium (1 ng/ml) + Streptomycin (100 g/ml) + Transferrin (1 µg/ml)
SR21	20 mg/ml (Not Specified)	Thrombin (50 IU/ml; Not Specified)	Not Used or Non Specified	Yes	1	2	N/A	N/A	N/A	Gel	3D; Composite; Acellular	No	N/A
SR22	1.5 mg/ml (Final)	Thrombin (0.6 U/ml; Stock)	Not Used or Non Specified	No	N/A	N/A	N/A	N/A	N/A	Hydrogel	3D; Acellular	No	N/A
SR23	2 wt% (Stock)	Thrombin (5 U/ml; Stock)	*CaCl2* (50 mM; Stock)	Yes	1	N/A	N/A	N/A	N/A	Hydrogel	3D; Composite; Cell-embedded; Meshed	Yes	N/A
SR24	Not Specified	Not Used or Non Specified	Not Used or Non Specified	Yes	N/A	N/A	1	N/A	N/A	Gel	3D; Thick; Cytodex Bead-embedded; Cell-embedded	Yes	Aprotinin (0.15 U/mL)
SR25	70 - 110 mg/ml (Stock)	Thrombin (37 U/ml; Not Specified)	$CaCl_2$ (40 mmol; Stock)	No	N/A	N/A	N/A	N/A	N/A	Gel	3D; Layered; Cell Sheet-stacked; Cell-embedded	Yes	N/A
SR26	2.5 mg/ml (Final)	Thrombin (0.2 U/ml; Final)	Not Used or Non Specified	No	N/A	N/A	N/A	N/A	N/A	Hydrogel	3D; Cell-embedded; Co-culture	Yes	Aprotinin (100 KIU/ml)
SR27	6 mg/ml (Stock)	Thrombin (0.32 U/ml; Stock)	Not Used or Non Specified	Yes	N/A	N/A	N/A	N/A	1	Hydrogel	3D; Co-culture; Cell-embedded	Yes	Aprotinin (92.5 µg/ml) + Tranexamic acid (400 µM)
SR28	5 mg/ml (core matrix with cells) surrounded by 25 mg/ml (acellular capsule) (Final)	Thrombin (2.5 U/ml; Stock)	$CaCl_2$ (40 mM ; Stock)	No	N/A	N/A	N/A	N/A	N/A	Gel	3D; Cell-embedded	Yes	N/A
SR29	2.5 mg/ml (Stock)	Thrombin (0.32 U/ml; Stock)	Not Used or Non Specified	Yes	1	2	N/A	N/A	1	Gel	3D; Cell-embedded	Yes	N/A
SR30	1 mg (Not Specified)	Thrombin (0.2 U; Not Specified)	Not Used or Non Specified	Yes	1	N/A	N/A	N/A	N/A	Hydrogel	3D; Multilayered; Cell-embedded; Composite	Yes	N/A
SR31	2.5 mg/ml (Stock)	Thrombin (0.625 U/well; Stock)	Not Used or Non Specified	Yes	1	N/A	N/A	N/A	N/A	Hydrogel	3D; Composite; Cell-embedded	Yes	N/A
SR32	25 mg/ml (Stock)	Thrombin (75 NIH U/ml; Stock)	$CaCl_2$ (4 ml; Stock)	No	N/A	N/A	N/A	N/A	N/A	Gel	3D; Composite; Cell-embedded	Yes	N/A
SR33	3 mg/ml (Stock)	Thrombin (50 U/ml; Stock)	Not Used or Non Specified	Yes	N/A	2	N/A	N/A	N/A	Gel	3D; Microcarrier Bead-embedded; Cell-embedded	Yes	N/A
SR34	50 mg/ml (Stock)	Thrombin (20 IU/ml; Stock)	Not Used or Non Specified	Yes	2	N/A	N/A	N/A	N/A	Microfibre Hydrogel	3D; Multicellular; Multilayered; Cell-embedded	Yes	Aminocaproic Acid (30 mM) + VEGF (50 ng/ml)
SR35	0.01 v/v% (Final)	Not Used or Non Specified	Not Used or Non Specified	No	N/A	N/A	N/A	N/A	N/A	Gel	3D; Cell-embedded	Yes	N/A
SR36	Not Specified	Thrombin (NS; Not Specified)	Not Used or Non Specified	Yes	N/A	2	1	N/A	N/A	Gel	3D; Overlay on Cells	Yes	bFGF (50 ng/ml) + VEGF (100 ng/ml)
SR37	24 mg/ml (Stock)	Thrombin (5 IU/ml; Stock)	Not Used or Non Specified	No	N/A	N/A	N/A	N/A	N/A	Gel	3D; Acellular	No	N/A
SR38	1 mg/ml (Stock)	Thrombin (0.625 U/ml; Stock)	Not Used or Non Specified	Yes	N/A	5	N/A	N/A	N/A	Hydrogel	3D; Factor-decorated; *In Situ;* Acellular	No	N/A
SR39	9.4, 94.3 or 188.6 mg/ml (Stock)	Thrombin (3.3, 33.3 or 66.66 mg/ml; Stock)	$CaCl_2$ (5.9 mg/ml; Not Specified)	Yes	1	N/A	N/A	N/A	N/A	Hydrogel	3D; Composite; Cell-embedded	Yes	N/A
SR40	Not Specified	Thrombin (NS; Not Specified)	Not Used or Non Specified	Yes	N/A	1	1	N/A	N/A	Patch	3D; NRG-1-loaded; Composite; Nanometric Fibrous Structure; Acellular	No	N/A
SR41	11/mg/ml (Stock)	Thrombin (1 kU/ml; Stock)	$CaCl_2$ (40 mM ; Stock)	No	N/A	N/A	N/A	N/A	N/A	Glue	3D; *In Situ;* Cell-embedded	Yes	N/A
SR42	2.5 mg/ml (Stock)	Thrombin (50 U/ml; Stock)	Not Used or Non Specified	Yes	1	N/A	N/A	N/A	N/A	Sponge	3D; Composite; Interconnected Porous Structure; Slow Degradation; Stabilised; Freeze-dried; Cell-embedded	Yes	N/A
SR43	2.5, 5, 10 or 20 mg/ml (Not Specified)	Thrombin (0.2 IU/ml; Not Specified)	Not Used or Non Specified	Yes	1	N/A	N/A	N/A	N/A	Hydrogel	3D; Composite; Microtissue-based; Cell-embedded	Yes	N/A
SR44	2 mg/ml (Not Specified)	Thrombin (4 U/ml; Not Specified)	Not Used or Non Specified	Yes	N/A	N/A	N/A	N/A	1	Hydrogel	3D; Nanosphere-embedded; Acellular	No	N/A
SR45	10 mg/ml (Not Specified)	Thrombin (2 IU/ml; Not Specified)	Not Used or Non Specified	Yes	N/A	5	1	N/A	N/A	Matrix	3D; Bilayered; Cell-embedded; Spheroid-embedded	Yes	N/A
SR46	100 mg/ml (Stock)	Thrombin (500 IU/ml; Stock)	$CaCl_2$ (5.9 mg/ml; Stock)	Yes	N/A	3	N/A	N/A	N/A	Hydrogel	3D; VEGF Controlled Delivery; *In Situ;* Acellular	No	N/A
SR47	10 mg/ml (Final)	Thrombin (100 U/ml; Stock)	Not Used or Non Specified	No	N/A	N/A	N/A	N/A	N/A	Patch	3D; Cell-embedded	Yes	N/A
SR48	40 mg/ml (Stock)	Thrombin (10 U/ml; Final)	$CaCl_2$ (40 mM; Not Specified)	Yes	N/A	N/A	N/A	1	2	Gel	3D; Ordered Nano-structured; Microbead-embedded; Magnetically-guided; Self-assembled; Acellular	No	N/A
SR49	1.5 mg/ml (Not Specified)	Thrombin (0.625 NIH U/ml; Not Specified)	Not Used or Non Specified	No	N/A	N/A	N/A	N/A	N/A	Gel	3D; Co-culture; Cell-embedded; (group 3 & 4)Cytodex Bead-embedded	Yes	Aprotinin (100 KIU/ml)
SR50	60 mg/ml (Stock)	Thrombin (1250 U/ml; Stock)	$CaCl_2$ (275 mM; Stock)	Yes	N/A	2	N/A	N/A	N/A	Gel	3D; Cell-embedded	Yes	N/A
SR51	2.5 - 25 mg/ml (Not Specified)	Thrombin (2 U/ml; Not Specified)	$CaCl_2$ (2.5 nM; Not Specified)	Yes	N/A	1	N/A	N/A	N/A	Gel	3D; Cell-embedded; Co-culture	Yes	N/A
SR52	32 mg/ml (Stock)	Thrombin (25 U/ml; Stock)	$CaCl_2$ (40 mM ; Stock)	Yes	1	N/A	N/A	N/A	N/A	Scaffold	3D; Nanofibrous; Bilayered; Electrospun; Acellular	No	N/A
SR53	10 or 20 mg/ml (Stock)	Thrombin (10 U/ml; Stock)	$CaCl_2$ (0.063 M; Stock)	Yes	N/A	2	N/A	N/A	1	Gel	3D; Peptide-DNA Nanoparticle-embedded; Acellular	No	N/A
SR54	0.1 (Stock)	Thrombin (50 U/ml; Stock)	$CaCl_2$ (7%) or $MgCl_2$ (3%) (0.07; Stock)	Yes	1	N/A	N/A	N/A	N/A	Microfibre Hydrogel	3D; Electrospun; Acellular; Small-diametre; Composite	No	N/A
SR55	4 mg/ml (Stock)	Thrombin (0.5 U/ml; Stock)	Not Used or Non Specified	Yes	1	2	N/A	N/A	1	Gel	3D; Functionalised; Composite; Porous; Freeze-dried; Acellular	No	N/A
SR56	Not Specified	Not Used or Non Specified	Not Used or Non Specified	Yes	N/A	3	N/A	N/A	N/A	Gel-Coacervate System	3D; Composite; Controlled Delivery System; Acellular	No	N/A

Study Code (cont.)	Fibrin Concentration and Formulation Stage (cont.)	Coagulant, with Concentration and Formulation Stage (cont.)	Crosslinker, with Concentration and Formulation Stage (cont.)	Were Additional Materials Used in the Formulation? (cont.)	Number of Different Polymers (Natural & Synthetic) Used (cont.)	Number of Different Growth Factors, Peptides, Amino Acids & Enzymes Used (cont.)	Number of Different Cell Media & Solutions Used (cont.)	Number of Different Chemicals Used (cont.)	Number of Different Materials Used (Others) (cont.)	Manufactured Object (cont.)	Manufactured Object Features (cont.)	Were Cells Pre-embedded into the Scaffold? (cont.)	Factors Added to the Standard Cell Medium with Concentrations (cont.)
SR57	70 - 110 mg/ml (Stock)	Thrombin (NS; Not Specified)	$CaCl_2$ (40 mM ; Stock)	Yes	N/A	1	N/A	N/A	N/A	Matrix	3D; Cell-embedded	Yes	FGF-2 (10 ng/ml) + TNFa (10 ng/ml) + VEGF (25 ng/ml)
SR58	10 mg/ml (Stock)	Thrombin (2 U/ml; Stock)	$CaCl_2$ (2.5 or 5 mM; Stock)	Yes	N/A	3	1	N/A	N/A	Gel	3D; Co-culture; Cell-embedded	Yes	Aprotinin (200 U/ml) + bFGF (30 ng/ml) + VEGF (100 ng/ml)
SR59	2.5 mg/ml (Final)	Thrombin (4 U/ml; Stock)	$CaCl_2$ (NS; Not Specified)	Yes	N/A	2	1	N/A	N/A	Gel	3D; bFGF-treated; Acellular	No	N/A
SR60	Not Specified	Not Used or Non Specified	Not Used or Non Specified	No	N/A	N/A	N/A	N/A	N/A	Gel + Mesh	3D; Cell-embedded; Composite	Yes	N/A
SR61	10 wt% (Final)	Not Used or Non Specified	Not Used or Non Specified	No	N/A	N/A	N/A	N/A	N/A	Matrix	3D; Cell-embedded; *In Situ* Delivery	Yes	N/A
SR62	10 mg/ml (Not Specified)	Not Used or Non Specified	Not Used or Non Specified	Yes	1	N/A	1	N/A	N/A	Scaffold	3D; Macroporous; Freeze-dried; Coated; Cell-embedded; Co-culture	Yes	bFGF (10 or 20 ng/ml) + Human Serum (2%) + Hydrocortisone (20 ng/ml) + VEGF (10 or 20 ng/ml)
SR63	10 mg/ml (Stock)	Thrombin (20 U/ml; Stock)	$CaCl_2$ (50 mM; Stock)	Yes	1	1	N/A	N/A	N/A	Matrix	(1) 3D; Bilayered; Composite (2) 3D; Bilayered; Composite; Sealant Solution-Impregnated (3) 3D; Bilayered; Composite; VEGF and bFGF Growth Factor-embedded; Acellular	No	N/A
SR64	20 mg/ml (Stock)	Thrombin (100 IU/ml; Stock)	Not Used or Non Specified	Yes	N/A	2	N/A	N/A	N/A	Gel	3D; Porous; Acellular	No	N/A
SR65	20 mg/ml (Stock)	Thrombin (1 mg/ml; Stock)	*NaCl* (0.009; Stock)	Yes	N/A	N/A	N/A	N/A	1	Scaffold	3D; Cell-embedded	Yes	N/A
SR66	2 mg/ml (Final)	Thrombin (0.5 U/ml; Final)	$CaCl_2$ (2.5 mM; Final)	Yes	1	N/A	N/A	N/A	N/A	Scaffold	3D; HA-modified; Composite; Sheet-based; Acellular	No	N/A
SR67	10 or 20 mg/ml (Final)	Thrombin (0.8 U/ml; Stock)	Not Used or Non Specified	Yes	N/A	2	N/A	2	N/A	Patch	3D; Composite; Flexible; Acellular	No	N/A
SR68	20 mg/ml (Not Specified)	Thrombin (3 U/ml; Final)	Not Used or Non Specified	Yes	1	2	N/A	N/A	1	Sealant	3D; Degradable; DNA-Delivery; VEGF-encoding; Acellular	No	N/A
SR69	5 mg/ml (Stock)	Thrombin (0.2 ml; Stock)	Not Used or Non Specified	Yes	1	N/A	N/A	N/A	1	Hydrogel	3D; Magnetic-responsive; Aligned; Nanoparticle-embedded; Acellular	No	N/A
SR70	12 µl (Stock)	Thrombin (NS; Not Specified)	Not Used or Non Specified	Yes	2	N/A	N/A	N/A	1	Hydrogel	3D; Composite; Bioprinted; Cell-embedded	Yes	6-Aminocaproic Acid (2mg/ml)
SR71	50 mg/ml (Stock)	Thrombin (50 U/ml; Stock)	Not Used or Non Specified	Yes	N/A	N/A	1	N/A	N/A	Gel	3D; *In Situ;* Acellular	No	N/A
SR72	100 mg/ml (Not Specified)	Thrombin (400 IU/ml; Not Specified)	Not Used or Non Specified	Yes	2	N/A	N/A	N/A	N/A	Microfibre Hydrogel	3D; Hierarchically Aligned; Nanofibre-based; Electrospun; Acellular	No	N/A
SR73	70 - 110 mg/ml (Stock)	Thrombin (500 IU/ml; Not Specified)	Not Used or Non Specified	Yes	N/A	2	1	N/A	N/A	Matrix	3D; Composite; and either Acellular or Cell-embedded	Both	Aprotinin (200 U/ml)
SR74	4 wt% (Stock)	Thrombin (15 U/ml; Stock)	$CaCl_2$ (50 mM; Stock)	Yes	2	N/A	1	N/A	N/A	Hydrogel	3D; Interconnected Channels; 3D Printed; Cell-embedded	Yes	N/A
SR75	0.01 (Stock)	Thrombin (20 U/ml; Stock)	$CaCl_2$ (50 mM; Not Specified)	Yes	N/A	4	N/A	N/A	N/A	Gel	3D; Cell-embedded	Yes	N/A
SR76	2 - 9 mg/ml (Stock)	Thrombin (37.5 U/ml; Final)	$CaCl_2$ (40 mmol; Stock)	Yes	1	N/A	N/A	N/A	N/A	Hydrogel	3D; PIGF-secreting; MMP9-secreting; Composite; Cell-embedded	Yes	N/A
SR77	6 mg/ml (Final)	Thrombin (1 U/ml; Stock)	Not Used or Non Specified	Yes	N/A	1	N/A	N/A	N/A	Gel	3D; HGF-loaded; Extruded Microthread-embedded; Acellular	No	N/A
SR78	Not Specified	Not Used or Non Specified	Not Used or Non Specified	Yes	N/A	1	N/A	N/A	N/A	Gel	3D; Acellular	No	N/A
SR79	70 mg/ml (Stock)	Thrombin (6 U/ml; Final)	$CaCl_2$ (40 mM ; Stock)	Yes	1	N/A	N/A	N/A	N/A	Hydrogel	3D; Cell-embedded; Composite	Yes	EGM-2 Supplements, which are: Ascorbic Acid (0.1%) + hEGF (0.1%) + Foetal Bovine Serum (2%) + hFGF-b (0.4%) + GA-1000 (0.1%) + Heparin (0.1%) + Hydrocortisone (0.04%) + R3-IGF-1 (0.1%) + VEGF (0.1%)
SR80	Not Specified	Thrombin (20 U/ml; Stock)	$CaCl_2$ (11.1 mM; Stock)	Yes	N/A	2	N/A	N/A	N/A	Gel	3D; Cell-embedded; Co-culture	Yes	Dexamethasone (1 µM) + Gentamycin (0.5%) + Indomethacin (0.2 mM) + Insulin (0.01 mg/ml) + 3-isobutyl-I-methyl-xanthine (0.5 mM)
SR81	8 mg/ml (Stock)	Thrombin (2.5 mU/ml; Stock)	Not Used or Non Specified	Yes	1	1	N/A	N/A	N/A	Scaffold	3D; *In Situ;* Angiogenic Factor-infiltrated; Cell-embedded	Yes	N/A

TABLE S5 Fibrinogen, Coagulant, and Crosslinker Formulations Used in the Corpus, by article, outlining the concentration used at the reported formulation step as reported by the authors of the study, as well as the type of coagulant or crosslinker chosen. NS: Not Specified; N: Not Reported; N/A: Non-Applicable.

	Fibrin		Coagulant			Crosslinker		
	Concentration	Stage	Coagulant	Concentration	Stage	Crosslinker	Concentration	Stage
1	20 mg/ml	Final	Thrombin	1250 U/ml	NS	$CaCl_2$	275 mM	NS
2	1.5 mg/ml	Final	Thrombin	0.6 U/ml	Stock	N	N/A	N/A
3	2 wt%	Stock	Thrombin	5 U/ml	Stock	$CaCl_2$	50 mM	Stock
4	NS	NS	N	N/A	N/A	N	N/A	N/A
5	70 - 110 mg/ml	Stock	Thrombin	37 U/ml	NS	$CaCl_2$	40 mmol	Stock
6	2.5 mg/ml	Final	Thrombin	0.2 U/ml	Final	N	N/A	N/A
7	6 mg/ml	Stock	Thrombin	0.32 U/ml	Stock	N	N/A	N/A
8	5 mg/ml (core matrix with cells) surrounded by 25 mg/ml (acellular capsule)	Final	Thrombin	2.5 U/ml	Stock	$CaCl_2$	40 mM	Stock
9	NS	NS	N	N/A	N/A	N	N/A	N/A
10	2.5 mg/ml	Stock	Thrombin	0.32 U/ml	Stock	N	N/A	N/A
11	1 mg	NS	Thrombin	0.2 U	NS	N	N/A	N/A
12	2.5 mg/ml	Stock	Thrombin	0.625 U/well	Stock	N	N/A	N/A
13	25 mg/ml	Stock	Thrombin	75 NIH U/ml	Stock	$CaCl_2$	4 ml	Stock
14	10 mg/ml	Final	Thrombin	11 µl at 100 U/ml	Stock	*NaCl*	NS	NS
15	3 mg/ml	Stock	Thrombin	50 U/ml	Stock	N	N/A	N/A
16	50 mg/ml	Stock	Thrombin	20 IU/ml	Stock	N	N/A	N/A
17	0.01 v/v%	Final	N	N/A	N/A	N	N/A	N/A
18	NS	NS	Thrombin	NS	NS	N	N/A	N/A
19	24 mg/ml	Stock	Thrombin	5 IU/ml	Stock	N	N/A	N/A
20	1 mg/ml	Stock	Thrombin	0.625 U/ml	Stock	N	N/A	N/A
21	9.4, 94.3 or 188.6 mg/ml	Stock	Thrombin	3.3, 33.3 or 66.66 mg/ml	Stock	$CaCl_2$	5.9 mg/ml	NS
22	NS	NS	Thrombin	NS	NS	N	N/A	N/A
23	11/mg/ml	Stock	Thrombin	1 kU/ml	Stock	$CaCl_2$	40 mM	Stock
24	2.5 mg/ml	Stock	Thrombin	50 U/ml	Stock	N	N/A	N/A
25	NS	NS	Thrombin	NS	NS	N	N/A	N/A
26	2.5, 5, 10 or 20 mg/ml	NS	Thrombin	0.2 IU/ml	NS	N	N/A	N/A
27	2 mg/ml	NS	Thrombin	4 U/ml	NS	N	N/A	N/A
28	10 mg/ml	NS	Thrombin	2 IU/ml	NS	N	N/A	N/A
29	100 mg/ml	Stock	Thrombin	500 IU/ml	Stock	$CaCl_2$	5.9 mg/ml	Stock
30	10 mg/ml	Final	Thrombin	100 U/ml	Stock	N	N/A	N/A
31	40 mg/ml	Stock	Thrombin	10 U/ml	Final	$CaCl_2$	40 mM	NS
32	NS	NS	N	N/A	N/A	N	N/A	N/A
33	1.5 mg/ml	NS	Thrombin	0.625 NIH U/ml	NS	N	N/A	N/A
34	2 wt%	Stock	Thrombin	10 U/ml	Stock	$CaCl_2$	50 mM	Stock
35	60 mg/ml	Stock	Thrombin	1250 U/ml	Stock	$CaCl_2$	275 mM	Stock
36	1 mg/ml	NS	Thrombin	0.3 U/ml	NS	N	N/A	N/A
37	NS	NS	Thrombin	500 U/ml	Stock	N	N/A	N/A
38	2.5 - 25 mg/ml	NS	Thrombin	2 U/ml	NS	$CaCl_2$	2.5 nM	NS
39	32 mg/ml	Stock	Thrombin	25 U/ml	Stock	$CaCl_2$	40 mM	Stock
40	10 or 20 mg/ml	Stock	Thrombin	10 U/ml	Stock	$CaCl_2$	0.063 M	Stock
41	NS	NS	Thrombin	NS	NS	N	N/A	N/A
42	10%	Stock	Thrombin	50 U/ml	Stock	$CaCl_2$	7%	Stock
						$MgCl_2$	3%	Stock
43	4 mg/ml	Stock	Thrombin	0.5 U/ml	Stock	N	N/A	N/A
44	NS	NS	N	N/A	N/A	N	N/A	N/A
45	70 - 110 mg/ml	Stock	Thrombin	NS	NS	$CaCl_2$	40 mM	Stock
46	10 mg/ml	Stock	Thrombin	2 U/ml	Stock	$CaCl_2$	2.5 or 5 mM	Stock
47	20 mg/ml	Stock	Thrombin	4 U/ml	Stock	N	N/A	N/A

	Fibrin (cont.)		Coagulant (cont.)			Crosslinker (cont.)		
	**Concentration**	**Stage**	**Coagulant**	**Concentration**	**Stage**	**Crosslinker**	**Concentration**	**Stage**
48	10 mg/ml	NS	Thrombin	50 U/ml	Stock	$CaCl_2$	150 mM	Stock
49	2.5 mg/ml	Final	Thrombin	4 U/ml	Stock	$CaCl_2$	NS	NS
50	36.59 or 23.59 mg/ml	Stock	Thrombin	223 kIU/ml or 144.6 kIU/ml	Stock	N	N/A	N/A
51	NS	NS	N	N/A	N/A	N	N/A	N/A
52	10 wt%	Final	N	N/A	N/A	N	N/A	N/A
53	10 mg/ml	NS	N	N/A	N/A	N	N/A	N/A
54	10 mg/ml	Stock	Thrombin	20 U/ml	Stock	$CaCl_2$	50 mM	Stock
55	20 mg/ml	Stock	Thrombin	100 IU/ml	Stock	N	N/A	N/A
56	20 mg/ml	Stock	Thrombin	1 mg/ml	Stock	*NaCl*	0.90%	Stock
57	2 mg/ml	Final	Thrombin	0.5 U/ml	Final	$CaCl_2$	2.5 mM	Final
58	2.5 mg/ml	Stock	Thrombin	2.5 U/ml	Final	N	N/A	N/A
59	3 mg/ml	Final	Thrombin	30 U/ml	Stock	$CaCl_2$	100 M	Stock
60	10 or 20 mg/ml	Final	Thrombin	0.8 U/ml	Stock	N	N/A	N/A
61	20 mg/ml	NS	Thrombin	3 U/ml	Final	N	N/A	N/A
62	5 mg/ml	Stock	Thrombin	0.2 ml	Stock	N	N/A	N/A
63	12 µl	Stock	Thrombin	NS	NS	N	N/A	N/A
64	NS	NS	Thrombin	NS	NS	N	N/A	N/A
65	50 mg/ml	Stock	Thrombin	50 U/ml	Stock	N	N/A	N/A
66	20 mg/ml	NS	Thrombin	50 IU/ml	NS	N	N/A	N/A
67	100 mg/ml	NS	Thrombin	400 IU/ml	NS	N	N/A	N/A
68	70 - 110 mg/ml	Stock	Thrombin	500 IU/ml	NS	N	N/A	N/A
69	4 wt%	Stock	Thrombin	15 U/ml	Stock	$CaCl_2$	50 mM	Stock
70	10 mg/ml	Final	Thrombin	50 U/ml	Final	N	N/A	N/A
71	8 mg/ml	Final	Thrombin	2 U/ml	Final	Ca^{2+}	2.5 mmol/L	Final
72	1%	Stock	Thrombin	20 U/ml	Stock	$CaCl_2$	50 mM	NS
73	2 - 9 mg/ml	Stock	Thrombin	37.5 U/ml	Final	$CaCl_2$	40 mmol	Stock
74	2%	Stock	Thrombin	100 IU/ml	Final	N	N/A	N/A
75	6 mg/ml	Final	Thrombin	1 U/ml	Stock	N	N/A	N/A
76	NS	NS	N	N/A	N/A	N	N/A	N/A
77	70 mg/ml	Stock	Thrombin	6 U/ml	Final	$CaCl_2$	40 mM	Stock
78	0.2 mg/ml	Stock	Thrombin	0.2 ml	Stock	*NaCl*	0.90%	Stock
79	NS	NS	Thrombin	20 U/ml	Stock	$CaCl_2$	11.1 mM	Stock
80	NS	NS	Thrombin	20 µl	Stock	N	N/A	N/A
81	8 mg/ml	Stock	Thrombin	2.5 mU/ml	Stock	N	N/A	N/A

TABLE S6 Polymers, both natural and synthetic, used in the fibrin scaffolds by frequency of use in the studies included in the corpus.

Polymers (Natural & Synthetic)	n
Collagen	7
Polyethylene Oxide (PEO)	4
Alginate	3
Fibronectin	3
Heparin	3
Polyethylene Glycol (PEG)	3
Succinimidyl Glutarate-modified Polyethylene Glycol	3
Hyaluronic Acid	2
Alginate (Chemically Modified)	1
Chitosan	1
Dacron	1
Hyaluronic Acid	1
Oxidised Dextran	1
Poly Lactic - *co* - Glycolic Acid (PLGA)	1
Polycaprolactone (PCL)	1
Polydimethylsiloxane (PDMS)	1
Polydioxanone (PDO)	1
Quaternized β-Chitin Derivative	1
s-IPN PEtU-PDMS	1

TABLE S7 **Growth Factors, Peptides, Amino Acids and Enzymes used in the fibrin scaffolds** by frequency of use in the studies included in the corpus.

Growth Factors, Peptides, Amino Acids & Enzymes	n
Aprotinin	19
Basic Fibroblast Growth Factor (bFGF)	11
Vascular Endothelial Growth Factor (VEGF)	8
Factor XIII	6
Fibroblast growth factor 2 (FGF-2)	3
Factor XIIIa	2
Glycine	2
Human Recombinant Basic Fibroblast Growth Factor (hr-bFGF)	2
Plasminogen	2
Platelet-derived Growth Factor-BB (PDGF-BB)	2
Transforming Growth Factor β1 (TGF-β1)	2
α-Interferon	1
Bovine Serum Albumin (BSA)	1
Epidermal Growth Factor (EGF)	1
Fibroblast Growth Factor 1 (FGF-1)	1
Hematopoietic Cytokine Erythropoietin	1
Hepatocyte Growth Factor (HGF)	1
(Human) Factor XIIIa	1
Human Recombinant Vascular Endothelial Growth Factor (hr-VEGF)	1
Human Serum Albumin (HSA)	1
Insulin	1
Keratinocyte Growth Factor (KGF)	1
L- Arginine	1
L- Lysine	1
Neuregulin-1 (NRG-1)	1
Penicillin	1
Platelet Lysate	1
Platelet-derived Growth Factor (PDGF)	1
Selenium	1
Streptomycin	1
Thrombospondin-1 (TSP-1)	1
Transferrin	1
Transglutaminase	1
Vascular Endothelial Growth Factor Isoform 164 ($VEGF_{164}$)	1
Vascular Endothelial Growth Factor Isoform 165 ($VEGF_{165}$)	1

TABLE S8 Cell Media & Solutions used in the fibrin scaffolds by frequency of use in the studies included in the corpus.

Cell Media & Solutions	n
Dulbecco's Modified Eagle Medium (DMEM)	2
Foetal Bovine Serum (FBS)	2
M199	2
NaCl	2
Endothelial Basal Medium (EBM)	1
Endothelial Cell Growth Medium 2 (EGM-2)	1
Endothelial Cell Growth Supplement	1
Foetal Calf Serum (FCS)	1
Hank's Balanced Salt Solution (HBSS)	1
HEPES	1
Serum	1
Tris-Buffered Saline (TBS) Solution	1

TABLE S9 Chemicals used in the fibrin scaffolds by frequency of use in the studies included in the corpus.

Chemicals	n
ε-Aminocaproic Acid	1
Tranexamic Acid	1
Trisodium Citrate	1
Triton-X	1

TABLE S10 Other materials used in the fibrin scaffolds by frequency of use in the studies included in the corpus.

Others	n
Cytodex-3 Microcarriers	2
Matrigel	2
Bevacizumab	1
Cytodex Beads	1
Deoxyribonucleic acid (DNA)	1
Fe_3O_4 Magnetic Nanoparticles	1
Heparin-Conjugated PLGA Nanospheres	1
Lithium Phthalocyanine Crystals	1
Plasmids	1
Plasmin Inhibitor Aprotinin Solution	1
Thrombin Coated Tosyl-Activated Superparamagnetic Beads	1
Tosyl-Activated Superparamagnetic Beads	1

TABLE S11 **Composition of Modified Cell Media Used for Fibrin Scaffolds**, by the number of scaffolds in which a given combination was used.

Factors Used to Supplement the Cell Medium	n
Aprotinin (100 kIU/ml)	3
Aprotinin (200 U/ml)**	2
VEGF (50 ng/ml)	2
6-Aminocaproic Acid (2mg/ml)	1
Aminocaproic Acid (30 mM) + VEGF (50 ng/ml)	1
Amphotericin (0.25 μg/ml) + Aprotinin (100 kIU/ml) + Heparan Sulfate (5 U/ml)*	1
Amphotericin (0.25 μg/ml) + Aprotinin (100 kIU/ml) + Heparan Sulfate (5 U/ml) + Methocel Solution (20%)*	1
Angiopoietin-1 (1-125 ng/ml) + bFGF (25 ng/ml) + TGF-β (0.5-2 ng/ml) + VEGF (25 ng/ml)	1
Aprotinin (0.15 U/ml)	1
Aprotinin (100 U/ml) + L-Ascorbic Acid-2-Phosphate (50 μg/ml) + FGF (40 ng/ml) + VEGF (40 ng/ml)	1
Aprotinin (200 U/ml) + bFGF (30 ng/ml) + VEGF (100 ng/ml)	1
Aprotinin (200 U/ml) + hr-bFGF (50 ng/ml) + hr-VEGF (40 ng/ml)**	1
Aprotinin (92.5 μg/ml) + Tranexamic acid (400 μM)	1
Ascorbic Acid (0.1%) + hEGF (0.1%) + Foetal Bovine Serum (2%) + hFGF-b (0.4%) + GA-1000 (0.1%) + Heparin (0.1%) + Hydrocortisone (0.04%) + R3-IGF-1 (0.1%) + VEGF (0.1%)	1
Dexamethasone (1 μM) + Foetal Calf Serum (10%) + Gentamycin (0.5%) + Indomethacin (0.2 mM) + Insulin (0.01 mg/ml) + 3-isobutyl- I-methyl-xanthine (0.5 mM)	1
Dexamethasone (1 μM) + Gentamycin (0.5%) + Indomethacin (0.2 mM) + Insulin (0.01 mg/ml) + 3-isobutyl-I-methyl-xanthine (0.5 mM)	1
bFGF (10 or 20 ng/ml) + Human Serum (2%) + Hydrocortisone (20 ng/ml) + VEGF (10 or 20 ng/ml)	1
bFGF (50 ng/ml) + VEGF (100 ng/ml)	1
FGF-2 (10 ng/ml) + TNFa (10 ng/ml) + VEGF (25 ng/ml)	1
Insulin (1 μg/ml) + Penicillin (100 U/ml) + Selenium (1 ng/ml) + Streptomycin (100 g/ml) + Transferrin (1 μg/ml)	1

** Used in the same publication.*

*** Used in the same publication.*

References

[1] Deng Pan et al. "A Feasibility Study Transplanting Macrophages to a Segmental Nerve Injury". In: *Muscle & Nerve* 68 (6 2023), pp. 894–900. ISSN: 1097-4598. DOI: 10.1002/mus.27977.

[2] Matthias A. Reichenberger et al. "ADSCs in a Fibrin Matrix Enhance Nerve Regeneration After Epineural Suturing in a Rat Model". In: *Microsurgery* 36 (6 2016), pp. 491–500. ISSN: 1098-2752. DOI: 10.1002/micr.30018.

[3] I. U. Allan et al. "An *In Vitro* Evaluation of Fibrinogen and Gelatin Containing Cryogels as Dermal Regeneration Scaffolds". In: *Biomaterials Science* 4 (6 2016), pp. 1007–1014. ISSN: 2047-4849. DOI: 10.1039/c6bm00133e.

[4] Kevin J. Cronin et al. "The Role of Biological Extracellular Matrix Scaffolds in Vascularized Three-Dimensional Tissue Growth *In Vivo*". In: *Journal of Biomedical Materials Research - Part B Applied Biomaterials* 82 (1 2007), pp. 122–128. ISSN: 15524981. DOI: 10.1002/jbm.b.30713.

[5] Victor V. Nikolaychik et al. "Biodesign of a Skeletal Muscle Flap as a Model for Cardiac Assistance". In: *Artificial Organs* 24 (2 2000), pp. 137–147. ISSN: 0160564X. DOI: 10.1046/j.1525-1594.2000.024002137.x.

[6] Amandine F.G. Godier-Furnémont et al. "Composite Scaffold Provides a Cell Delivery Platform for Cardiovascular Repair". In: *Proceedings of the National Academy of Sciences of the United States of America* 108 (19 2011), pp. 7974–7979. ISSN: 00278424. DOI: 10.1073/pnas.1104619108.

[7] Ivan Hadad et al. "Development of a Porcine Delayed Wound-Healing Model and Its Use in Testing a Novel Cell-Based Therapy". In: *International Journal of Radiation Oncology Biology Physics* 78 (3 2010), pp. 888–896. ISSN: 03603016. DOI: 10.1016/j.ijrobp.2010.05.002.

[8] Cheuk-Kwan Sun et al. "Direct Implantation Versus Platelet-Rich Fibrin-embedded Adipose-derived Mesenchymal Stem Cells in Treating Rat Acute Myocardial Infarction". In: *International Journal of Cardiology* 173 (3 2014), pp. 410–423. ISSN: 18741754. DOI: 10.1016/j.ijcard.2014.03.015.

[9] Deliang Shen et al. "Effects of Matrix Metalloproteinases on the Performance of Platelet Fibrin Gel Spiked With Cardiac Stem Cells in Heart Repair". In: *Stem Cells Translational Medicine* 5 (6 2016), pp. 793–803. ISSN: 2157-6564. DOI: 10.5966/sctm.2015-0194.

[10] Eunna Chung et al. "Evaluation of Gold Nanotracers to Track Adipose-derived Stem Cells in a PEGylated Fibrin Gel for Dermal Tissue Engineering Applications". In: *International Journal of Nanomedicine* 8 (1 2013), pp. 325–336. ISSN: 11769114. DOI: 10.2147/IJN.S36711.

[11] H.F. Dvorak et al. "Fibrin Containing Gels Induce Angiogenesis. Implications for Tumor Stroma Generation and Wound Healing". In: *Laboratory Investigation* 57 (6 1987), pp. 673–686. URL: https://pubmed.ncbi.nlm.nih.gov/2447383/.

[12] Kihoon Nam et al. "Fibrin Hydrogels Fortified with FGF-7/10 and Laminin-1 Peptides Promote Regeneration of Irradiated Salivary Glands". In: *Acta Biomaterialia* 172 (2023), pp. 147–158. ISSN: 1742-7061. DOI: 10.1016/j.actbio.2023.10.013.

[13] Rok Humar et al. "Formation of New Blood Vessels in the Heart can be Studied in Cell Cultures". In: *ALTEX* 24 (2007), pp. 35–8. URL: https://pubmed.ncbi.nlm.nih.gov/19835053/.

[14] Ian Woods et al. "Harnessing Topographical & Biochemical Cues to Enhance Elastogenesis by Paediatric Cells for Cardiovascular Tissue Engineering Applications". In: *Biochemical and Biophysical Research Communications* 512 (2 2019), pp. 156–162. ISSN: 0006-291X. DOI: 10.1016/j.bbrc.2019.03.026.

[15] Motoharu Hojo et al. "Induction of Vascular Endothelial Growth Factor by Fibrin as a Dermal Substrate for Cultured Skin Substitute". In: *Plastic and Reconstructive Surgery* 111 (5 2003), pp. 1638–1645. ISSN: 00321052. DOI: 10.1097/01.PRS.0000053842.90564.26.

[16] Michelle McLuckie et al. "Lipoconstruct Surface Topography Grating Size Influences Vascularization Onset in the Dorsal Skinfold Chamber Model". In: *Acta Biomaterialia* 106 (2020), pp. 136–144. ISSN: 1742-7061. DOI: 10.1016/j.actbio.2020.01.050.

[17] F. D. Bookholt et al. "Mathematical Modelling of Angiogenesis Using Continuous Cell-based Models". In: *Biomechanics and Modeling in Mechanobiology* 15 (6 2016), pp. 1577–1600. ISSN: 16177940. DOI: 10.1007/s10237-016-0784-3.

[18] Nobuaki Hiraoka et al. "Matrix Metalloproteinases Regulate Neovascularization by Acting as Pericellular Fibrinolysins". In: *Cell* 95 (3 1998), pp. 365–77. ISSN: 0092-8674. DOI: 10.1016/s0092-8674(00)81768-7.

[19] Su Hyun Jung et al. "Nematic Fibrin Fibers Enabling Vascularized Thrombus Implants Facilitate Scarless Cutaneous Wound Healing". In: *Advanced Materials* 35 (25 2023), p. 2211149. ISSN: 15214095. DOI: 10.1002/adma.202211149.

[20] S Rohr et al. "Quantitative Image Analysis of Angiogenesis in Rats Implanted with a Fibrin Gel Chamber". In: *Nouvelle Revue Francaise d'Hematologie* 34 (4 1992), pp. 287–94. URL: https://pubmed.ncbi.nlm.nih.gov/1280355/.

[21] R. Montesano, P. Mouron, and L. Orci. "Vascular Outgrowths from Tissue Explants Embedded in Fibrin or Collagen Gels: A Simple *In Vitro* Model of Angiogenesis". In: *Cell Biology International Reports* 9 (10 1985), pp. 869–875. ISSN: 03091651. URL: https://pubmed.ncbi.nlm.nih.gov/2415260/.

[22] F. Paulhe et al. "Vascular Smooth Muscle Cell Spreading onto Fibrinogen Is Regulated by Calpains and Phospholipase C". In: *Biochemical and Biophysical Research Communications* 288 (4 2001), pp. 875–881. ISSN: 0006-291X. DOI: 10.1006/bbrc.2001.5859.

[23] A Gigante et al. "Platelet Rich Fibrin Matrix Effects on Skeletal Muscle Lesions: An Experimental Study". In: *Jounral of Biological Regulators & Homeostatic Agents* 26 (3 2012), pp. 475–84. URL: https://pubmed.ncbi.nlm.nih.gov/23034267/.

[24] Kassandra S. Thomson et al. "Prevascularized Microtemplated Fibrin Scaffolds for Cardiac Tissue Engineering Applications". In: *Tissue Engineering Part A* 19 (7-8 2013), pp. 967–977. ISSN: 1937335X. DOI: 10.1089/ten.tea.2012.0286.

[25] Michael Müller et al. "Printing Thermoresponsive Reverse Molds for the Creation of Patterned Two-component Hydrogels for 3D Cell Culture". In: *Journal of Visualized Experiments* (77 2013), e50632. ISSN: 1940-087X. DOI: 10.3791/50632.

[26] Isabel Sánchez-Muñoz et al. "The Use of Adipose Mesenchymal Stem Cells and Human Umbilical Vascular Endothelial Cells on a Fibrin Matrix for Endothelialized Skin Substitute". In: *Tissue Engineering Part A* 21 (1-2 2015), pp. 214–23. ISSN: 1937335X. DOI: 10.1089/ten.tea.2013.0626.

[27] Wenbin Nan et al. "Umbilical Cord Mesenchymal Stem Cells Combined With a Collagenfibrin Double-layered Membrane Accelerates Wound Healing". In: *Wounds* 27 (5 2015), pp. 134–40. URL: https://pubmed.ncbi.nlm.nih.gov/25965183/.

[28] Pengcheng Yang et al. "Fabrication of Chitin-Fibrin Hydrogels to Construct the 3D Artificial Extracellular Matrix Scaffold for Vascular Regeneration and Cardiac Tissue Engineering". In: *Journal of Biomedical Materials Research Part A* 112 (12 2024), pp. 2257–2272. ISSN: 1552-4965. DOI: 10.1002/jbm.a.37774.

[29] Ethan J. Vanderslice, Staunton G.H. Golding, and Jeffrey G. Jacot. "Vascularization of PEGylated Fibrin Hydrogels Increases the Proliferation of Human iPSC-Cardiomyocytes". In: *Journal of Biomedical Materials Research - Part A* 112 (4 2024), pp. 625–634. ISSN: 15524965. DOI: 10.1002/jbm.a.37662.

[30] Chun Yi Yang et al. "External Magnetic Field Non-invasively Stimulates Spinal Cord Regeneration in Rat *via* a Magnetic-responsive Aligned Fibrin Hydrogel". In: *Biofabrication* 15 (3 2023), p. 035022. ISSN: 1758-5090. DOI: 10.1088/1758-5090/acdbec.

[31] Shalini Dasgupta et al. "Chitosan-Collagen-Fibrinogen Uncrosslinked Scaffolds Possessing Skin Regeneration and Vascularization Potential". In: *Journal of Biomedical Materials Research. Part A* 111 (5 2023), pp. 725–739. ISSN: 1552-4965. DOI: 10.1002/jbm.a.37488.

[32] Tianqi Chang et al. "Fibrin-based Cardiac Patch Containing Neuregulin-1 for Heart Repair After Myocardial Infarction". In: *Colloids and Surfaces B: Biointerfaces* 220 (2022). ISSN: 18734367. DOI: 10.1016/j.colsurfb.2022.112936.

[33] Florian Helms et al. "An Encapsulated Fibrin-based Bioartificial Tissue Construct with Integrated Macrovessels, Microchannels, and Capillary Tubes". In: *Biotechnology and Bioengineering* 119 (8 2022), pp. 2239–2249. ISSN: 1097-0290. DOI: 10.1002/bit.28111.

[34] Billur Sezgin et al. "The Effects of Oral Mucosa-derived Heterotopic Fibroblasts on Cutaneous Wound Healing". In: *Journal of Plastic, Reconstructive & Aesthetic Surgery* 74 (10 2021), pp. 2751–2758. ISSN: 1748-6815. DOI: 10.1016/j.bjps.2021.02.011.

[35] Sebastian Heene et al. "Vascular Network Formation on Macroporous Polydioxanone Scaffolds". In: *Tissue Engineering Part A* 27 (19-20 2021), pp. 1239–1249. ISSN: 1937-335X. DOI: 10.1089/ten.tea.2020.0232.

[36] Ludovic Melly et al. "Fibrin Hydrogels Promote Scar Formation and Prevent Therapeutic Angiogenesis in the Heart". In: *Journal of Tissue Engineering and Regenerative Medicine* 14 (10 2020), pp. 1513–1523. ISSN: 19327005. DOI: 10.1002/term.3118.

[37] Mohammad Ali Nilforoushzadeh et al. "Engineered Skin Graft with Stromal Vascular Fraction Cells Encapsulated in Fibrin–Collagen Hydrogel: A Clinical Study for Diabetic Wound Healing". In: *Journal of Tissue Engineering and Regenerative Medicine* 14 (3 2020), pp. 424–440. ISSN: 19327005. DOI: 10.1002/term.3003.

[38] Morgan B. Elliott et al. "Regenerative and Durable Small-diameter Graft as an Arterial Conduit". In: *Proceedings of the National Academy of Sciences of the United States of America* 116 (26 2019), pp. 12710–12719. ISSN: 1091-6490. DOI: 10.1073/pnas.1905966116.

[39] Min Ah Koo et al. "Effective Stacking and Transplantation of Stem Cell Sheets Using Exogenous ROS-Producing Film for Accelerated Wound Healing". In: *Acta Biomaterialia* 95 (2019), pp. 418–426. ISSN: 18787568. DOI: 10.1016/j.actbio.2019.01.019.

[40] Mohamad Javad Mirzaei-Parsa et al. "Nanofiber-Acellular Dermal Matrix as a Bilayer Scaffold Containing Mesenchymal Stem Cell for Healing of Full-Thickness Skin Wounds". In: *Cell and Tissue Research* 375 (3 2019), pp. 709–721. ISSN: 1432-0878. DOI: 10.1007/s00441-018-2927-6.

[41] Shenglian Yao et al. "Hierarchically Aligned Fibrin Nanofiber Hydrogel Accelerated Axonal Regrowth and Locomotor Function Recovery in Rat Spinal Cord Injury". In: *International Journal of Nanomedicine* 13 (2018), p. 2883. ISSN: 11782013. DOI: 10.2147/ijn.s159356.

[42] A. A. Gorkun et al. "Angiogenic Potential of Spheroids from Umbilical Cord and Adipose-derived Multipotent Mesenchymal Stromal Cells within Fibrin Gel". In: *Biomedical Materials* 13 (4 2018). ISSN: 1748-605X. DOI: 10.1088/1748-605X/aac22d.

[43] David M. Burmeister et al. "Delivery of Allogeneic Adipose Stem Cells in Polyethylene Glycol-Fibrin Hydrogels as an Adjunct to Meshed Autografts After Sharp Debridement of Deep Partial Thickness Burns". In: *Stem Cells Translational Medicine* 7 (4 2018), pp. 360–372. ISSN: 21576580. DOI: 10.1002/sctm.17-0160.

[44] Rajan Narayan et al. "Goat Tendon Collagen-Human Fibrin Hydrogel for Comprehensive Parametric Evaluation of HUVEC Microtissue-Based Angiogenesis". In: *Colloids and Surfaces B: Biointerfaces* 163 (2018), pp. 291–300. ISSN: 1873-4367. DOI: 10.1016/j.colsurfb.2017.12.056.

[45] D. D. Manavella et al. "Adipose Tissue-derived Stem Cells in a Fibrin Implant Enhance Neovascularization in a Peritoneal Grafting Site: A Potential Way to Improve Ovarian Tissue Transplantation". In: *Human Reproduction* 33 (2 2018), pp. 270–279. ISSN: 0268-1161. DOI: 10.1093/humrep/dex374.

[46] K. P. Myu Mai Ja et al. "Construction of a Vascularized Hydrogel for Cardiac Tissue Formation in a Porcine Model". In: *Journal of Tissue Engineering and Regenerative Medicine* 12 (4 2018), e2029–e2038. ISSN: 1932-7005. DOI: 10.1002/term.2634.

[47] Elena V. Solovieva et al. "Fibrinogen-modified Sodium Alginate as a Scaffold Material for Skin Tissue Engineering". In: *Biomedical Materials* 13 (2 2018). ISSN: 1748-605X. DOI: 10.1088/1748-605X/aa9089.

[48] R.-X Zeng et al. "Experimental Study on Repairing Skin Defect by Tissue-Engineered Skin Substitute Compositely Constructed by Adipose-derived Stem Cells and Fibrin Gel". In: *European Review for Medical and Pharmacological Sciences* (21 2017), p. 1. URL: https://pubmed.ncbi.nlm.nih.gov/28745800/.

[49] Tobias Hasenberg et al. "Emulating Human Microcapillaries in a Multi-Organ-Chip Platform". In: *Journal of Biotechnology* 216 (2015), pp. 1–10. ISSN: 18734863. DOI: 10.1016/j.jbiotec.2015.09.038.

[50] Jonathan M. Grasman et al. "Rapid Release of Growth Factors Regenerates Force Output in Volumetric Muscle Loss Injuries". In: *Biomaterials* 72 (2015), p. 49. ISSN: 18785905. DOI: 10.1016/j.biomaterials.2015.08.047.

[51] Dacha Gholobova et al. "Endothelial Network Formation Within Human Tissue-Engineered Skeletal Muscle". In: *Tissue Engineering Part A* 21 (19-20 2015), pp. 2548–2558. ISSN: 1937-335X. DOI: 10.1089/ten.tea.2015.0093.

[52] Sebastian F. Barreto-Ortiz et al. "Fabrication of 3-dimensional Multicellular Microvascular Structures". In: *The FASEB Journal* 29 (8 2015), pp. 3302–3314. ISSN: 15306860. DOI: 10.1096/fj.14-263343.

[53] Hassan K. Awada, Noah R. Johnson, and Yadong Wang. "Sequential Delivery of Angiogenic Growth Factors Improves Revascularization and Heart Function After Myocardial Infarction". In: *Journal of Controlled Release* 207 (2015), pp. 7–17. ISSN: 1873-4995. DOI: 10.1016/j.jconrel.2015.03.034.

[54] Eunna Chung et al. "Fibrin-based 3D Matrices Induce Angiogenic Behavior of Adipose-derived Stem Cells". In: *Acta Biomaterialia* 17 (2015), pp. 78–88. ISSN: 18787568. DOI: 10.1016/j.actbio.2015.01.012.

[55] Valérie Bellamy et al. "Long-term Functional Benefits of Human Embryonic Stem Cell-derived Cardiac Progenitors Embedded into a Fibrin Scaffold". In: *The Journal of Heart and Lung Transplantation* 34 (9 2015), pp. 1198–1207. ISSN: 1557-3117. DOI: 10.1016/j.healun.2014.10.008.

[56] Julio J. Mendez et al. "Mesenchymal Stromal Cells Form Vascular Tubes when Placed in Fibrin Sealant and Accelerate Wound Healing *In Vivo*". In: *Biomaterials* 40 (2015), pp. 61–71. ISSN: 1878-5905. DOI: 10.1016/j.biomaterials.2014.11.011.

[57] Wan Geun La and Hee Seok Yang. "Heparin-Conjugated Poly(Lactic-Co-Glycolic Acid) Nanospheres Enhance Large-Wound Healing by Delivering Growth Factors in Platelet-Rich Plasma". In: *Artificial Organs* 39 (4 2015), pp. 388–394. ISSN: 1525-1594. DOI: 10.1111/aor.12389.

[58] Pavan Atluri et al. "Tissue-engineered, Hydrogel-based Endothelial Progenitor Cell Therapy Robustly Revascularizes Ischemic Myocardium and Preserves Ventricular Function". In: *The Journal of Thoracic and Cardiovascular Surgery* 148 (3 2014), pp. 1090–1098. ISSN: 1097-685X. DOI: 10.1016/j.jtcvs.2014.06.038.

[59] Sabrina Rohringer et al. "Mechanisms of Vasculogenesis in 3D Fibrin Matrices Mediated by the Interaction of Adipose-derived Stem Cells and Endothelial Cells". In: *Angiogenesis* 17 (4 2014), pp. 921–933. ISSN: 1573-7209. DOI: 10.1007/s10456-014-9439-0.

[60] Elim Y.L. Cheung et al. "Specific Effects of Fibrinogen and the γA and γ'-Chain Fibrinogen Variants on Angiogenesis and Wound Healing". In: *Tissue Engineering Part A* 21 (1-2 2015), pp. 106–114. ISSN: 1937-335X. DOI: 10.1089/ten.tea.2014.0020.

[61] Veronica Sacchi et al. "Long-lasting Fibrin Matrices Ensure Stable and Functional Angiogenesis by Highly Tunable, Sustained Delivery of Recombinant $VEGF_{164}$". In: *Proceedings of the National Academy of Sciences of the United States of America* 111 (19 2014), pp. 6952–6957. ISSN: 1091-6490. DOI: 10.1073/pnas.1404605111.

[62] C. Bearzi et al. "PlGF–MMP9-Engineered iPS Cells Supported on a PEG–fibrinogen Hydrogel Scaffold Possess an Enhanced Capacity to Repair Damaged Myocardium". In: *Cell Death & Disease* 5 (2 2014), e1053. ISSN: 20414889. DOI: 10.1038/cddis.2014.12.

[63] Daniela Marino et al. "Bioengineering Dermo-Epidermal Skin Grafts with Blood and Lymphatic Capillaries". In: *Science Translational Medicine* 6 (221 2014). ISSN: 1946-6242. DOI: 10.1126/scitranslmed.3006894.

[64] Nisarat Ruangsawasdi, Matthias Zehnder, and Franz E. Weber. "Fibrin Gel Improves Tissue Ingrowth and Cell Differentiation in Human Immature Premolars Implanted in Rats". In: *Journal of Endodontics* 40 (2 2014), pp. 246–250. ISSN: 0099-2399. DOI: 10.1016/j.joen.2013.09.022.

[65] Xinru Zhao et al. "*In Vitro* Vascularization of a Combined System Based on a 3D Printing Technique". In: *Journal of Tissue Engineering and Regenerative Medicine* 10 (10 2016), pp. 833–842. ISSN: 19327005. DOI: 10.1002/term.1863.

[66] Sebastian F. Barreto-Ortiz et al. "A Novel *In Vitro* Model for Microvasculature Reveals Regulation of Circumferential ECM Organization by Curvature". In: *PLoS One* 8 (11 2013). ISSN: 1932-6203. DOI: 10.1371/journal.pone.0081061.

[67] Areck A. Ucuzian et al. "Angiogenic Endothelial Cell Invasion into Fibrin is Stimulated by Proliferating Smooth Muscle Cells". In: *Microvascular Research* 90 (2013), pp. 40–47. ISSN: 1095-9319. DOI: 10.1016/j.mvr.2013.06.012.

[68] Suk Ho Bhang et al. "Mutual Effect of Subcutaneously Transplanted Human Adipose-derived Stem Cells and Pancreatic Islets Within Fibrin Gel". In: *Biomaterials* 34 (30 2013), pp. 7247–7256. ISSN: 1878-5905. DOI: 10.1016/j.biomaterials.2013.06.018.

[69] David O. Zamora et al. "Enhanced Wound Vascularization Using a dsASCs Seeded FPEG Scaffold". In: *Angiogenesis* 16 (4 2013), pp. 745–757. ISSN: 09696970. DOI: 10.1007/s10456-013-9352-y.

[70] Matteo Centola et al. "Scaffold-Based Delivery of a Clinically Relevant Anti-Angiogenic Drug Promotes the Formation of *In Vivo* Stable Cartilage". In: *Tissue Engineering Part A* 19 (17-18 2013), pp. 1960–1971. ISSN: 1937-335X. DOI: 10.1089/ten.tea.2012.0455.

[71] Makoto Horimizu et al. "An Improved Freeze-Dried PRP-Coated Biodegradable Material Suitable for Connective Tissue Regenerative Therapy". In: *Cryobiology* 66 (3 2013), pp. 223–232. ISSN: 1090-2392. DOI: 10.1016/j.cryobiol.2013.01.006.

[72] Aleksander Skardal et al. "Bioprinted Amniotic Fluid-derived Stem Cells Accelerate Healing of Large Skin Wounds". In: *Stem Cells Translational Medicine* 1 (11 2012), pp. 792–802. ISSN: 2157-6564. DOI: 10.5966/sctm.2012-0088.

[73] Wolfgang Holnthoner et al. "Adipose-derived Stem Cells Induce Vascular Tube Formation of Outgrowth Endothelial Cells in a Fibrin Matrix". In: *Journal of Tissue Engineering and Regenerative Medicine* 9 (2 2015), pp. 127–136. ISSN: 1932-7005. DOI: 10.1002/term.1620.

[74] Femke Verseijden et al. "Comparing Scaffold-free and Fibrin-based Adipose-derived Stromal Cell Constructs for Adipose Tissue Engineering: An *In Vitro* and *In Vivo* Study". In: *Cell Transplantation* 21 (10 2012), pp. 2283–2297. ISSN: 1555-3892. DOI: 10.3727/096368912x653129.

[75] Qiang Xiong et al. "Bioenergetic and Functional Consequences of Cellular Therapy: Activation of Endogenous Cardiovascular Progenitor Cells". In: *Circulation Research* 111 (4 2012), pp. 455–468. ISSN: 1524-4571. DOI: 10.1161/circresaha.112.269894.

[76] Antonella Lisi et al. "A Combined Synthetic-Fibrin Scaffold Supports Growth and Cardiomyogenic Commitment of Human Placental Derived Stem Cells". In: *PLoS One* 7 (4 2012). ISSN: 1932-6203. DOI: 10.1371/journal.pone.0034284.

[77] Matthias A. Reichenberger et al. "Fibrin-Embedded Adipose Derived Stem Cells Enhance Skin Flap Survival". In: *Stem Cell Reviews and Reports* 8 (3 2012), pp. 844–853. ISSN: 15508943. DOI: 10.1007/s12015-011-9341-8.

[78] T. V. Anilkumar et al. "Advantages of Hyaluronic Acid as a Component of Fibrin Sheet for Care of Acute Wound". In: *Biologicals* 39 (2 2011), pp. 81–88. ISSN: 1045-1056. DOI: 10.1016/j.biologicals.2011.01.003.

[79] Oliver Bleiziffer et al. "Endothelial Progenitor Cells are Integrated in Newly Formed Capillaries and Alter Adjacent Fibrovascular Tissue After Subcutaneous Implantation in a Fibrin Matrix". In: *Journal of Cellular and Molecular Medicine* 15 (11 2011), pp. 2452–2461. ISSN: 15821838. DOI: 10.1111/j.1582-4934.2010.01247.x.

[80] Femke Verseijden et al. "Vascularization of Prevascularized and Non-Prevascularized Fibrin-based Human Adipose Tissue Constructs After Implantation in Nude Mice". In: *Journal of Tissue Engineering and Regenerative Medicine* 6 (3 2012), pp. 169–178. ISSN: 1932-7005. DOI: 10.1002/term.410.

[81] M. C. Barsotti et al. "Fibrin Acts as Biomimetic Niche Inducing Both Differentiation and Stem Cell Marker Expression of Early Human Endothelial Progenitor Cells". In: *Cell Proliferation* 44 (1 2011), pp. 33–48. ISSN: 09607722. DOI: 10.1111/j.1365-2184.2010.00715.x.

[82] Liana M. Lugo, Pedro Lei, and Stelios T. Andreadis. "Vascularization of the Dermal Support Enhances Wound Re-Epithelialization by *In Situ* Delivery of Epidermal Keratinocytes". In: *Tissue Engineering Part A* 17 (5-6 2011), pp. 665–675. ISSN: 1937-335X. DOI: 10.1089/ten.tea.2010.0125.

[83] Xiaodong Shen, Kuniyoshi Tanaka, and Atsushi Takamori. "Coronary Arteries Angiogenesis in Ischemic Myocardium: Biocompatibility and Biodegradability of Various Hydrogels". In: *Artificial Organs* 33 (10 2009), pp. 781–787. ISSN: 1525-1594. DOI: 10.1111/J.1525-1594.2009.00815.x.

[84] Femke Verseijden et al. "Adult Human Bone Marrow- and Adipose Tissue-derived Stromal Cells Support the Formation of Prevascular-like Structures from Endothelial Cells *In Vitro*". In: *Tissue Engineering Part A* 16 (1 2010), pp. 101–114. ISSN: 1937-335X. DOI: 10.1089/ten.tea.2009.0106.

[85] Shigeaki Inoue et al. "The Effectiveness of Basic Fibroblast Growth Factor in Fibrin-based Cultured Skin Substitute *In Vivo*". In: *Journal of Burn Care & Research* 30 (3 2009), pp. 514–519. ISSN: 1559-047X. DOI: 10.1097/BCR.0b013e3181a28e4b.

[86] Seung-Woo Cho et al. "Engineered Adipose Tissue Formation Enhanced by Basic Fibroblast Growth Factor and a Mechanically Stable Environment". In: *Cell Transplantation* 16 (2007), pp. 421–434. ISSN: 1555-3892. DOI: 10.3727/000000007783464795.

[87] Nestor Torio-Padron et al. "Implantation of VEGF Transfected Preadipocytes Improves Vascularization of Fibrin Implants on the Cylinder Chorioallantoic Membrane (CAM) Model". In: *Minimally Invasive Therapy & Allied Technologies* 16 (3 2007), pp. 155–162. ISSN: 1364-5706. DOI: 10.1080/13645700701384116.

[88] Jörg Borges et al. "*In Vitro* Analysis of the Interactions Between Preadipocytes and Endothelial Cells in a 3D Fibrin Matrix". In: *Minimally Invasive Therapy & Allied Technologies* 16 (3 2007), pp. 141–148. ISSN: 1364-5706. DOI: 10.1080/13645700600935398.

[89] Eben Alsberg et al. "Magnetically-guided Self-assembly of Fibrin Matrices with Ordered Nano-scale Structure for Tissue Engineering". In: *Tissue Engineering* 12 (11 2006), pp. 3247–3256. ISSN: 1076-3279. DOI: 10.1089/ten.2006.12.3247.

[90] I. Wilcke et al. "VEGF^{165} and bFGF Protein-based Therapy in a Slow Release System to Improve Angiogenesis in a Bioartificial Dermal Substitute *In Vitro* and *In Vivo*". In: *Langenbeck's Archives of Surgery* 392 (3 2007), pp. 305–314. ISSN: 1435-2443. DOI: 10.1007/s00423-007-0194-1.

[91] Sabrina Kellouche et al. "Platelets, Thrombospondin-1 and Human Dermal Fibroblasts Cooperate for Stimulation of Endothelial Cell Tubulogenesis through VEGF and PAI-1 Regulation". In: *Experimental Cell Research* 313 (3 2007), pp. 486–499. ISSN: 0014-4827. DOI: 10.1016/j.yexcr.2006.10.023.

[92] Joerg Borges et al. "Adipose Precursor Cells (Preadipocytes) Induce Formation of New Vessels in Fibrin Glue on the Newly Developed Cylinder Chorioallantoic Membrane Model (CAM)". In: *Minimally Invasive Therapy & Allied Technologies* 15 (4 2006), pp. 246–252. ISSN: 1364-5706. DOI: 10.1080/14017450600761620.

[93] Diana Trentin et al. "Peptide-Matrix-mediated Gene Transfer of an Oxygen-insensitive Hypoxia-inducible Factor-1α Variant for Local Induction of Angiogenesis". In: *Proceedings of the National Academy of Sciences of the United States of America* 103 (8 2006), pp. 2506–2511. ISSN: 0027-8424. DOI: 10.1073/pnas.0505964102.

[94] Oju Jeon et al. "Control of Basic Fibroblast Growth Factor Release from Fibrin Gel with Heparin and Concentrations of Fibrinogen and Thrombin". In: *Journal of Controlled Release* 105 (3 2005), pp. 249–259. ISSN: 0168-3659. DOI: 10.1016/j.jconrel.2005.03.023.

[95] Craig K. Griffith et al. "Diffusion Limits of an *In Vitro* Thick Prevascularized Tissue". In: *Tissue Engineering* 11 (1-2 2005), pp. 257–266. ISSN: 1076-3279. DOI: 10.1089/ten.2005.11.257.

[96] Jingbo Liu et al. "Autologous Stem Cell Transplantation for Myocardial Repair". In: *American Journal of Physiology - Heart and Circulatory Physiology* 287 (2 56-2 2004), pp. 501–511. ISSN: 03636135. DOI: 10.1152/ajpheart.00019.2004.

[97] Zishan A. Haroon et al. "A Novel Role for Erythropoietin During Fibrin-Induced Wound-Healing Response ". In: *The American Journal of Pathology* 163 (3 2003), pp. 993–1000. ISSN: 0002-9440. DOI: 10.1016/S0002-9440(10)63459-1.

[98] Martin N. Nakatsu et al. "Angiogenic Sprouting and Capillary Lumen Formation Modeled by Human Umbilical Vein Endothelial Cells (HUVEC) in Fibrin Gels: The Role of Fibroblasts and Angiopoietin-1". In: *Microvascular Research* 66 (2 2003), pp. 102–112. ISSN: 00262862. DOI: 10.1016/s0026-2862(03)00045-1.

[99] Valeri Chekanov et al. "Transplantation of Autologous Endothelial Cells Induces Angiogenesis". In: *Pacing and Clinical Electrophysiology* 26 (1P2 2003), pp. 496–499. ISSN: 0147-8389. DOI: 10.1046/j.1460-9592.2003.00080.x.

[100] Alicja Jozkowicz et al. "Delivery of High Dose VEGF Plasmid Using Fibrin Carrier does not Influence its Angiogenic Potency". In: *International Journal of Artificial Organs* 26 (2 2003), pp. 161–169. ISSN: 0391-3988. DOI: 10.1177/039139880302600211.

[101] Eric M. Brey et al. "A Technique for Quantitative Three-dimensional Analysis of Microvascular Structure". In: *Microvascular Research* 63 (3 2002), pp. 279–294. ISSN: 00262862. DOI: 10.1006/mvre.2002.2395.

[102] Susan M. Dallabrida, Michelle A. De Sousa, and David H. Farrell. "Expression of Antisense to Integrin Subunit β_3 Inhibits Microvascular Endothelial Cell Capillary Tube Formation in Fibrin". In: *Journal of Biological Chemistry* 275 (41 2000), pp. 32281–32288. ISSN: 00219258. DOI: 10.1074/jbc.M001446200.

[103] Xiaodong Feng et al. "Fibrin and Collagen Differentially Regulate Human Dermal Microvascular Endothelial Cell Integrins: Stabilization of αv/β3 mRNA by Fibrin". In: *Journal of Investigative Dermatology* 113 (6 1999), pp. 913–919. ISSN: 0022202X. DOI: 10.1046/j.1523-1747.1999.00786.x.

[104] Volker Nehls et al. "Contact-dependent Inhibition of Angiogenesis by Cardiac Fibroblasts in Three-dimensional Fibrin Gels *In Vitro*: Implications for Microvascular Network Remodeling and Coronary Collateral Formation". In: *Cell and Tissue Research* 293 (3 1998), pp. 479–488. ISSN: 0302-766X. DOI: 10.1007/s004410051140.

[105] Nancy Fournier and Charles J. Doillon. "Biological Molecule-impregnated Polyester: An *In Vivo* Angiogenesis Study". In: *Biomaterials* 17 (17 1996), pp. 1659–1665. ISSN: 01429612. DOI: 10.1016/0142-9612(96)87645-9.

[106] Volker Nehls and Detlev Drenckhahn. "A Microcarrier-based Cocultivation System for the Investigation of Factors and Cells Involved in Angiogenesis in Three-dimensional Fibrin Matrices *In Vitro*". In: *Histochemistry and Cell Biology* 104 (6 1995), pp. 459–466. ISSN: 0948-6143. DOI: 10.1007/BF01464336.

[107] Volker Nehls, Elmar Schuchardt, and Detlev Drenckhahn. "The Effect of Fibroblasts, Vascular Smooth Muscle Cells, and Pericytes on Sprout Formation of Endothelial Cells in a Fibrin Gel Angiogenesis System". In: *Microvascular Research* 48 (3 1994), pp. 349–363. ISSN: 0026-2862. DOI: 10.1006/mvre.1994.1061.

[108] H F Dvorak et al. "Fibrin Gel Investment Associated with Line 1 And Line 10 Solid Tumor Growth, Angiogenesis, and Fibroplasia in Guinea Pigs. Role Of Cellular Immunity, Myofibroblasts, Microvascular Damage, and Infarction in Line 1 Tumor Regression". In: *Journal of the National Cancer Institute* 62 (6 1979), pp. 1459–72. URL: https://pubmed.ncbi.nlm.nih.gov/286118/.
